# Single-cell transcriptome reveals *Staphylococcus aureus* modulating fibroblast differentiation in the bone-implant interface

**DOI:** 10.1186/s10020-023-00632-7

**Published:** 2023-03-16

**Authors:** Jinlong Yu, Boyong Wang, Feiyang Zhang, Zun Ren, Feng Jiang, Musha Hamushan, Mingzhang Li, Geyong Guo, Hao Shen

**Affiliations:** Department of Orthopedics, Shanghai Sixth People’s Hospital, No. 600, Yi Shan Road, Shanghai, 200233 China

**Keywords:** *Staphylococcus aureus*, scRNA-seq, Fibroblast, Implant-associated infection

## Abstract

**Background:**

This study aimed to delineate the cell heterogeneity in the bone-implant interface and investigate the fibroblast responses to implant-associated *S. aureus* infection.

**Methods:**

Single-cell RNA sequencing of human periprosthetic tissues from patients with periprosthetic joint infection (PJI, n = 3) and patients with aseptic loosening (AL, n = 2) was performed. Cell type identities and gene expression profiles were analyzed to depict the single-cell landscape in the periprosthetic environment. In addition, 11 publicly available human scRNA-seq datasets were downloaded from GSE datasets and integrated with the in-house sequencing data to identify disease-specific fibroblast subtypes. Furthermore, fibroblast pseudotime trajectory analysis and Single-cell regulatory network inference and clustering (SCENIC) analysis were combined to identify transcription regulators responsible for fibroblast differentiation. Immunofluorescence was performed on the sequenced samples to validate the protein expression of the differentially expressed transcription regulators.

**Results:**

Eight major cell types were identified in the human bone-implant interface by analyzing 36,466 cells. Meta-analysis of fibroblasts scRNA-seq data found fibroblasts in the bone-implant interface express a high level of *CTHRC1*. We also found fibroblasts could differentiate into pro-inflammatory and matrix-producing phenotypes, each primarily presented in the PJI and AL groups, respectively. Furthermore, *NPAS2* and *TFEC* which are activated in PJI samples were suggested to induce pro-inflammatory polarization in fibroblasts, whereas *HMX1*, *SOX5*, *SOX9*, *ZIC1*, *ETS2*, and *FOXO1* are matrix-producing regulators. Meanwhile, we conducted a CMap analysis and identified forskolin as a potential regulator for fibroblast differentiation toward matrix-producing phenotypes.

**Conclusions:**

In this study, we discovered the existence of *CTHRC1*^+^ fibroblast in the bone-implant interface. Moreover, we revealed a bipolar mode of fibroblast differentiation and put forward the hypothesis that infection could modulate fibroblast toward a pro-inflammatory phenotype through *NPAS2* and *TFEC*.

**Supplementary Information:**

The online version contains supplementary material available at 10.1186/s10020-023-00632-7.

## Background

Orthopedic implants are routinely used for fixation of fractures, correction of deformities and joint replacements. The most commonly used orthopedic implants include titanium alloy, chromium-cobalt-molybd, PMMA, etc. (Filipović et al. 2020). Orthopedic implant-associated infection (IAI) is a devastating complication after orthopedic surgery. The treatment usually involves debridement, implant removal, and long-term antibiotic therapy. Nonetheless, the prognosis of orthopedic IAI is often unsatisfactory because of its propensity to cause chronic and relapsing infections, and even worse, amputations (Masters et al. 2022; Amin Yavari et al. 2020; Depypere et al. 2020; Abram et al. 1469).

Host responses to implant can be generally classified into four types (Long 2008), three of which are highly dependent on implant materials. The remaining one, which occurs in a wide range of biomaterials, involves the formation of a fibrous capsule at the implant-bone interface (Filipović et al. 2020; Pagán and Ramakrishnan 2018; Chung et al. 2020). It was this fibrous encapsulation (also known as pseudomembrane/pseudocapsule) that provides a microenvironment of depressed immunity for bacterial colonization and biofilm formation (Alhasan et al. 2022). Anderson et al. (2008) suggested that the fibrous capsule forms following a sequential host response: injury, blood–material interactions, provisional matrix formation, acute inflammation, chronic inflammation, granulation tissue development, foreign body reaction, and finally developed a fibrous capsule. Despite all of the above-mentioned findings, our current understanding of detailed tissue-implant responses remains largely inadequate. The scRNA-seq technique is thus an appropriate method to study the tissue-implant interface in depth (Arciola et al. 2018). A recent review by Feng et al. (2023) summarized the current single-cell omics used in musculoskeletal disorders and we found no research on bone-implant interface.

According to epidemiology studies of our groups and others, the most frequently isolated pathogen in IAI is methicillin-resistant *Staphylococcus aureus* (MRSA) and methicillin-sensitive *Staphylococcus aureus* (MSSA) (Depypere et al. 2020; Guo et al. 2017; Patel 2023). Over the last decades, there has been intensive research regarding *S. aureus* implant infections which mainly focused on pathogen virulences and their interactions with immune cells (Matsumoto et al. 2021; He et al. 2022, 2019; Yamada et al. 2020; Heim et al. 2020). Nevertheless, few studies on fibroblast response to *S. aureus* infection were undertaken. It is reported that stromal cells such as fibroblasts play equally important roles in infectious diseases (Davidson et al. 2021; John et al. 2021; Iwanaga et al. 2021). Therefore, detailed and comprehensive studies on fibroblast biological responses toward *S. aureus* infection are required for a better understanding of IAI pathogenesis.

In the present study, we collected and sequenced periprosthetic samples from patients with *S. aureus* periprosthetic joint infections (PJI, n = 3) and patients with periprosthetic aseptic loosening (AL, n = 2). We for the first time depicted the complex cell populations in the bone-implant interface. Single-cell trajectory analysis highlights transcription regulators critical for fibroblast functional differentiation. Taken together, our findings, with a focus on fibroblast, enrich our understanding of the cellular and molecular response in IAI occurrence and could provide novel insights into the pathogenesis of IAI.

## Results

### Human bone-implant interface consists of eight major cell types

**Fig. 1:**
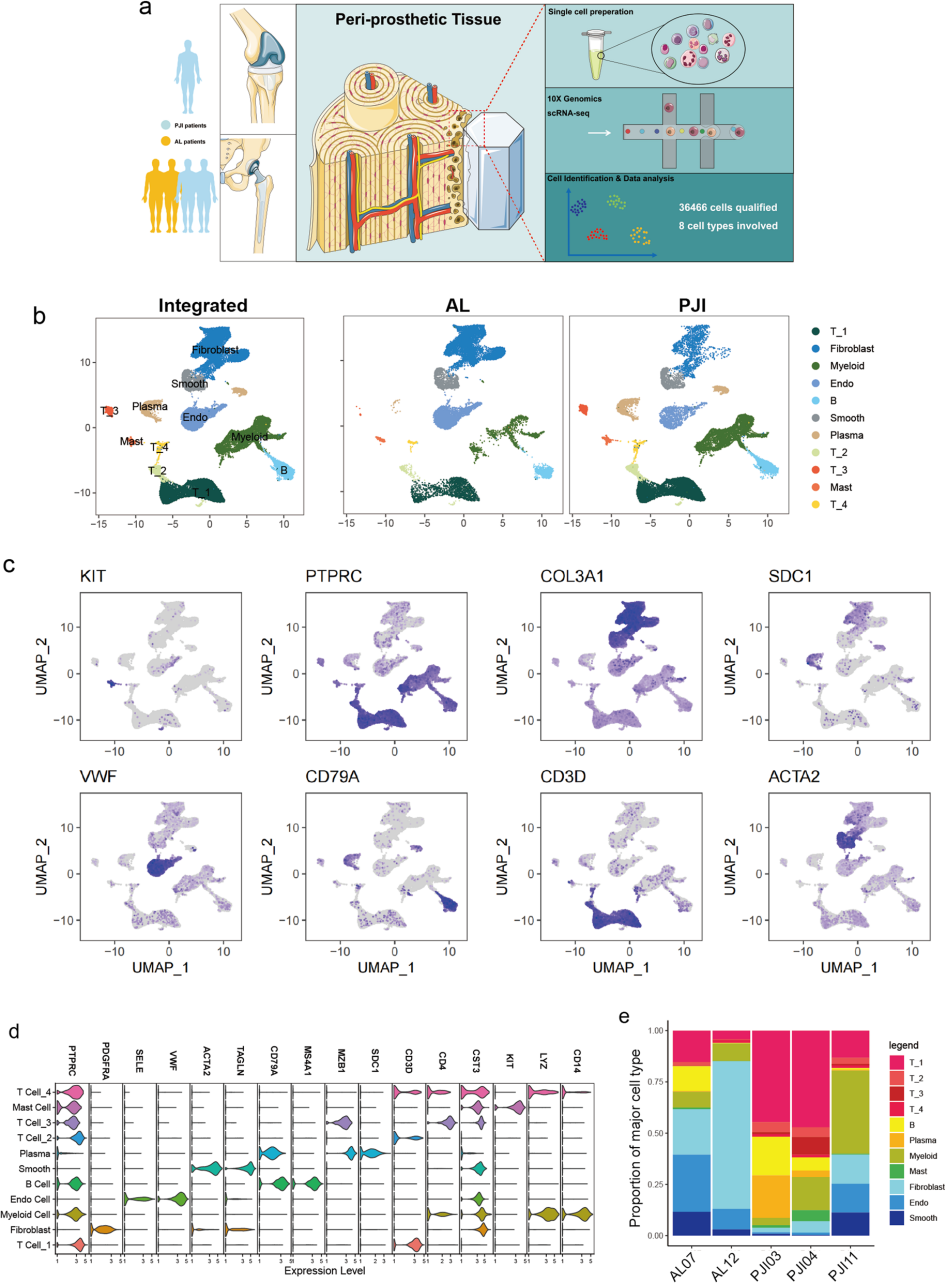
Single-cell RNA sequencing reveals cell heterogeneity in the periprosthetic environment. **a** Schematic overview showing study workflow from sampling to single-cell sequencing and data analysis. **b** UMAP visualization of total cells from the periprosthetic tissue of patients with AL and PJI, single cells are colored by cluster annotation. **c** Colored UMAP plot showing marker genes for each type of cell. **d** Violin plots showing the selected canonical marker genes across the annotated cell clusters. Expression levels are normalized and log transformed. **e** Stacked bar plot showing proportions of annotated cell clusters in each sample

Five fresh peri-implant tissues were collected from three patients with PJI and two patients with AL for scRNA-seq as illustrated in Fig. 1a, clinical characteristics and implant characteristics were presented in Additional file 1: Table S1. It is noteworthy that PJI11 was collected from patient with acute infection while PJI03, PJI04 were derived from chronic infection patients. After a series of quality control procedures (Additional file 2: Fig. S1a, b), a total of 36,466 cells were qualified for subsequent analysis with 21,982 and 14,484 cells from PJI and AL, respectively. Eight major cell types were manually annotated according to canonical cell marker genes and eleven cell clusters were confirmed with dimension reduction and unsupervised clustering (Fig. 1b, Additional file 3: Fig S2a). Specifically, the following cells were identified by their distinct marker genes: fibroblasts (PDGFRA, COL3A1), endothelial cells (SELE, VWF), smooth muscle cells (ACTA2, TAGLN), B cells (CD79A, MS4A1), T1-4 cells (CD3D, CD4), plasma cells (MZB1, SDC1), mast cells (CST3, KIT), myeloid cells (LYZ, CD14) (Fig. 1c, d). Gene ontology enrichment analysis revealed their corresponding functions in immune modulation and tissue construction (Additional file 2: Fig. S1d).

Compared with the PJI group, the AL group exhibited a higher proportion of stromal cells including fibroblasts (43.45 vs. 7.53%), endothelial cells (20.27 vs. 5.54%), and smooth muscle cells (7.92 vs. 4.33%), whereas the PJI group consists of a higher percentage of immune cells such as T cells (43.26 vs. 12.5%) and myeloid cells (20.68% vs 8.06%). It is noteworthy that plasma cells are specifically presented in the PJI group and rarely detected in the AL group (7.87% vs. 0.19%) (Fig. 1e, Additional file 2: Fig. S1c).

### Fibroblasts, T_1 cells and myeloid cells are the major modulators in the bone-implant interface

**Fig. 2:**
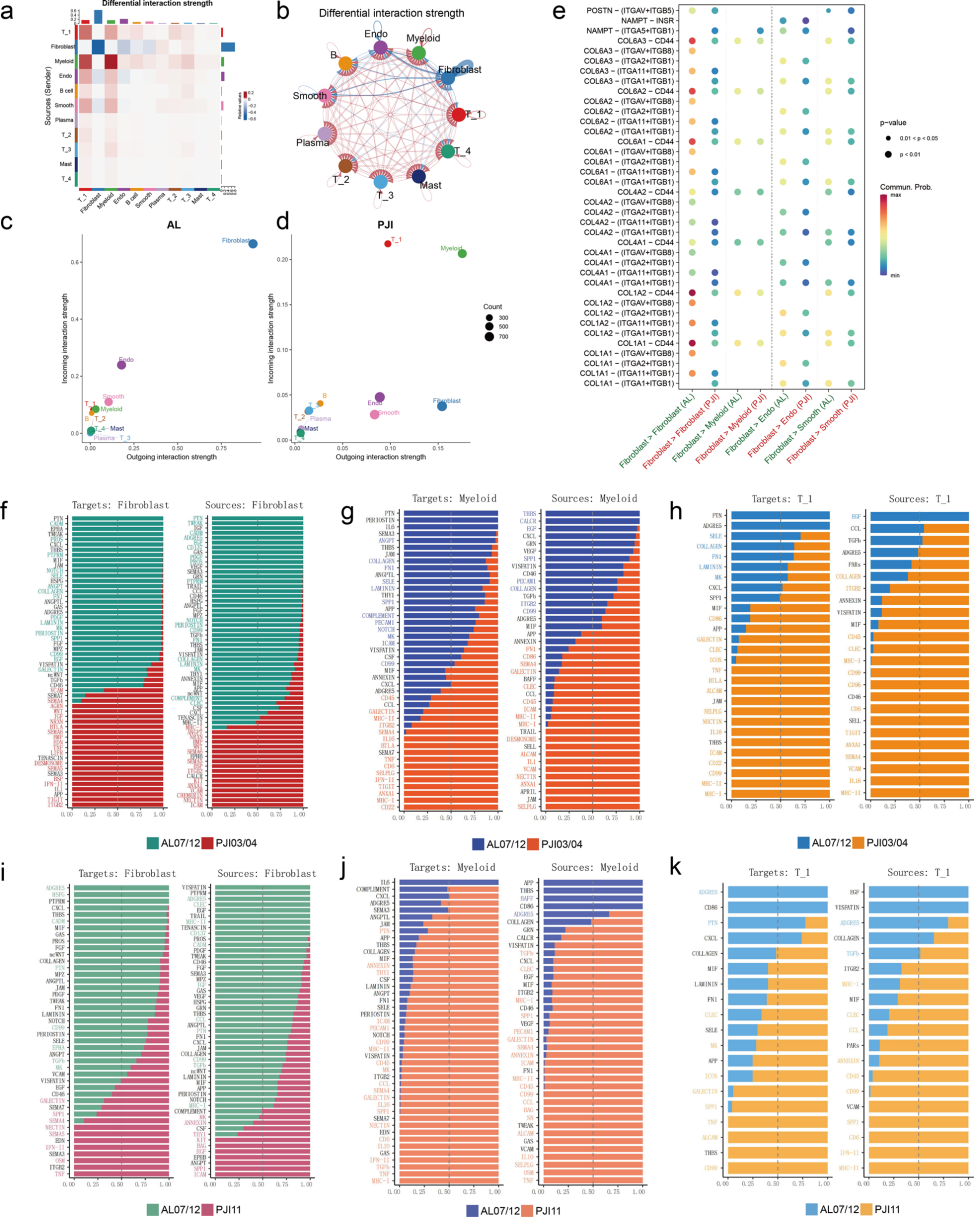
Cell–cell interaction analysis reveals differences in the major mediators between AL and PJI. **a**, **b** Heatmap and circos plot shows differential interaction strength relative to PJI, red indicates increased interaction strength and blue indicates decreased strength in PJI. **c**, **d** Scatter plot shows incoming interaction strength (y-axis) and outcoming interaction strength (x-axis) for each cell cluster in AL (**c**) and PJI (**d**) samples. **e** The dotted heatmap shows major differentially expressed ligand-receptor pairs in AL and PJI groups. **f**–**k** Bar plots list the relative strength of pathways from and target to Fibroblasts (**f**, **i**)/Myeloid cells (**g**, **j**)/T_1 cells (**h**, **k**)

A comparison of cell–cell interactions between PJI and AL groups showed stronger crosstalks among immune cells, but significantly downregulated fibroblasts–fibroblasts communication in the PJI group (Fig. 2a, b, Additional file 4: Fig. S3a–f). By computing the strengths of incoming and outgoing signals, we found that fibroblasts play a major cell–cell communication role in an aseptic environment (Fig. 2c, Additional file 4: Fig. S3j, k). In contrast, immune cells such as myeloid and T_1 cells contributed more frequently to cell communications in an infected environment (Fig. 2d, Additional file 4: Fig. S3g–i). Moreover, receptor-ligand enrichment indicated that downregulated communications in PJI mainly concentrated in the collagen pathway and PTN pathway which are strongly associated with fibroblast functions (Fig. 2e, f, i). By analyzing immune cell-dependent intercellular communications, much higher communication strength relating to myeloid was observed in the acute PJI group (Fig. 2g, j). The upregulated pathways are predominantly responsible for chemokines (IL10, IL16) and antigen processing (MHC-I, MHC-II). On the contrary, the chronic PJI group mainly dependent on T_1 cell interaction (Fig. 2h, k).

**Fig. 3:**
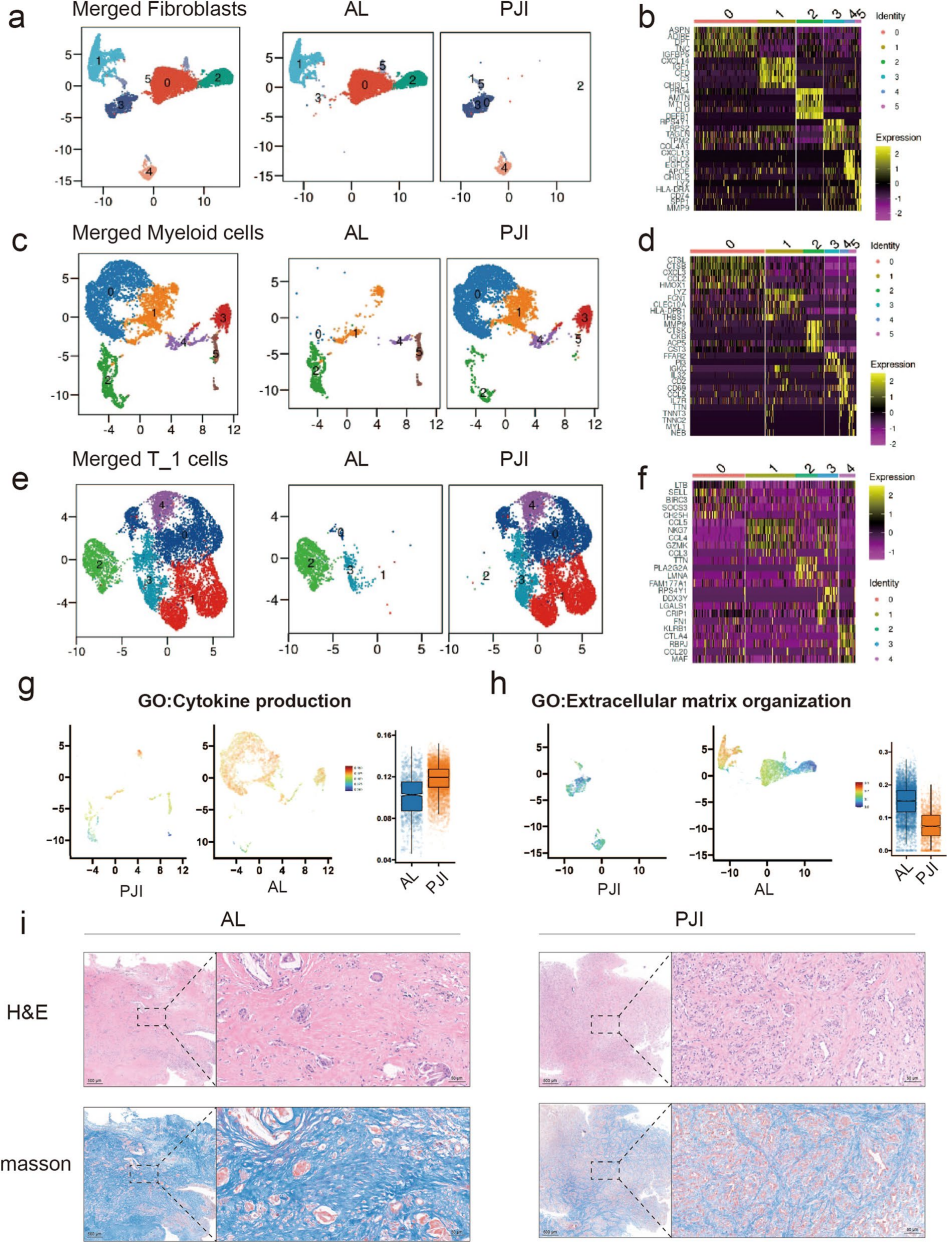
Fibroblast, myeloid cells and T_1 cells subtype analysis revealed cell functional differences. **a**, **c**, **d** UMAP visualization of fibroblasts (**a**)/myeloid cells (**c**)/T_1 cells **d** from the periprosthetic tissue of patients with AL and PJI, cells are colored by subtypes. **b**, **d**, **f** Heatmap displayed the highly expressed marker genes for each fibroblast (**b**)/myeloid cell (**d**)/T_1 cell (**f**) subclusters. **g** AUCell quantification of Gene ontology term for Cytokine production in myeloid cells. **h** AUCell quantification of Gene ontology term for extracellular matrix organization in fibroblasts. **i** Images of HE and Masson staining results for AL and PJI samples

Because fibroblast, myeloid cells and T_1 cells were identified as the major regulators, we decided to further compare their differences between AL and PJI groups. Fibroblasts/myeloid cells were classified into 6 subtypes and T_1 cells were classified into 5 subtypes according to their transcriptome heterogeneity (Fig. 3a, c, e Additional file 3: Fig. S2b–d), Fibro_0,1,2, Myeloid_2,5,4 and T_1 subtype 2 were mainly presented in the AL group while Fibro_3,4, Myeloid_0,1,3,4 and T_1 subtype 0,1,3,4 predominantly existed in the PJI groups. The representative marker for each cell subtype were provided in Fig. 3b, d, f. The upregulated genes of fibroblasts in the PJI group were mainly enriched in inflammatory pathways and the downregulated genes were responsible for extracellular matrix production and copper ion detoxification (Fig. 3h, Additional file 5: Fig. S4a, b). Myeloid cells in the PJI group were upregulated in immune-modulating pathways (Fig. 3g, Additional file 5: Fig. S4c, d). T_1 cells in the PJI groups were upregulated in lymphocyte/leukocyte activation (Additional file 5: Fig. S4e, f). H&E staining results revealed that the PJI samples have more immune cell infiltration. Masson staining showed that samples from the AL group exhibited a higher level of collagen deposition (Fig. 3i).

### Fibroblasts in the bone-implant interface are mainly *CTHRC1*^+^

**Fig. 4:**
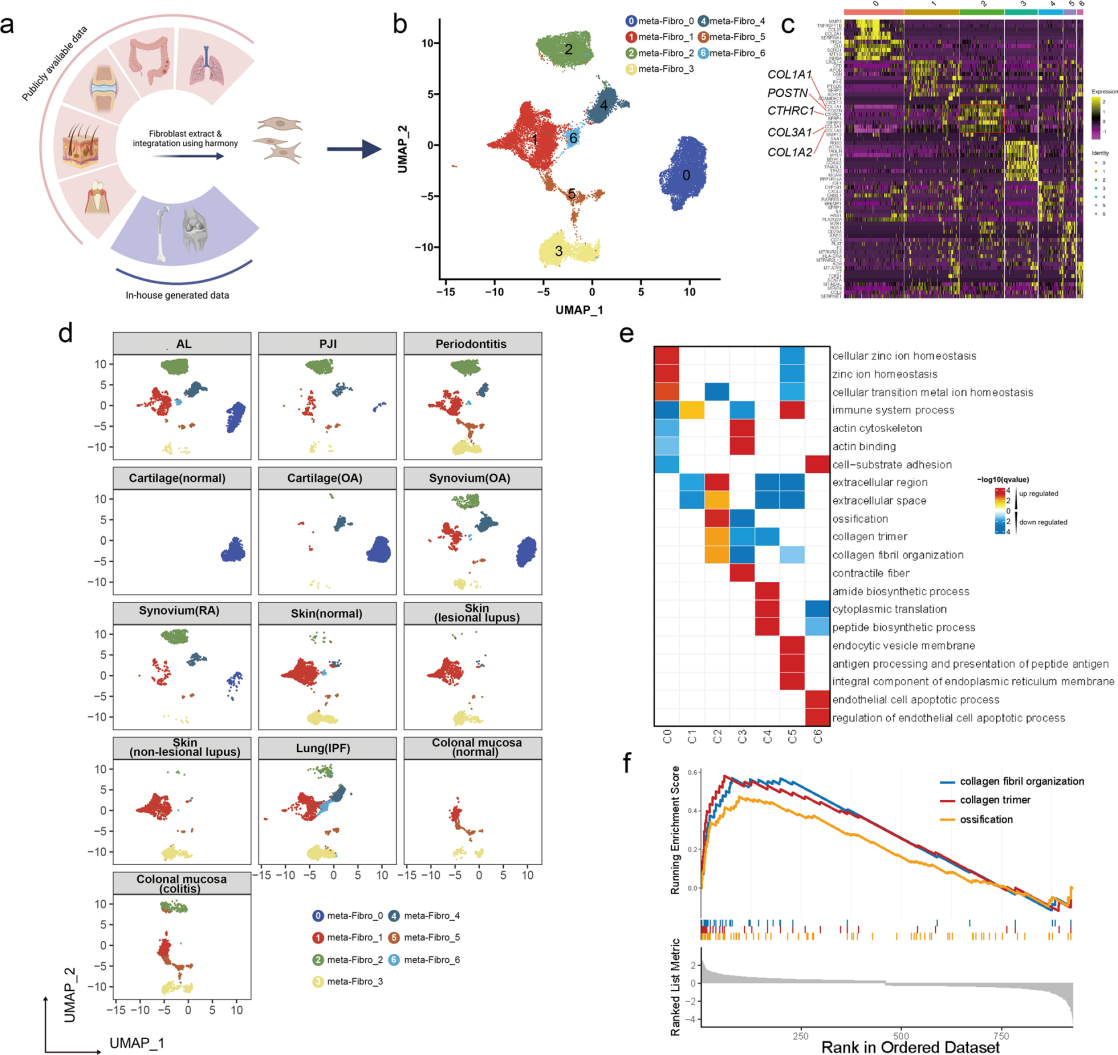
Meta-analysis of human fibroblast scRNA-seq data. **a** Illustration of the selected scRNA-seq data from GEO publicly available database. scRNA-seq derived from different organs were acquired from the GEO database and integrated with the in-house generated scRNA-seq data, then fibroblast scRNA-seq was extracted for further meta-analysis. **(b)** UMAP visualization of the fibroblast, cells are colored according to cell meta-clusters. **c** Heatmap displayed the highly expressed marker genes for each fibroblast meta-clusters. **d** UMAP visualization of the fibroblast in each sample. **e** The heatmap shows the GSEA enrichment result for each of the fibroblast cell meta-clusters. GO:BP databases were used in this analysis. **f** GSEA results shows three GO terms (ossification, collagen trimer, collagen fibril organization) are enriched in the fibroblast meta-cluster 2

**Fig. 5:**
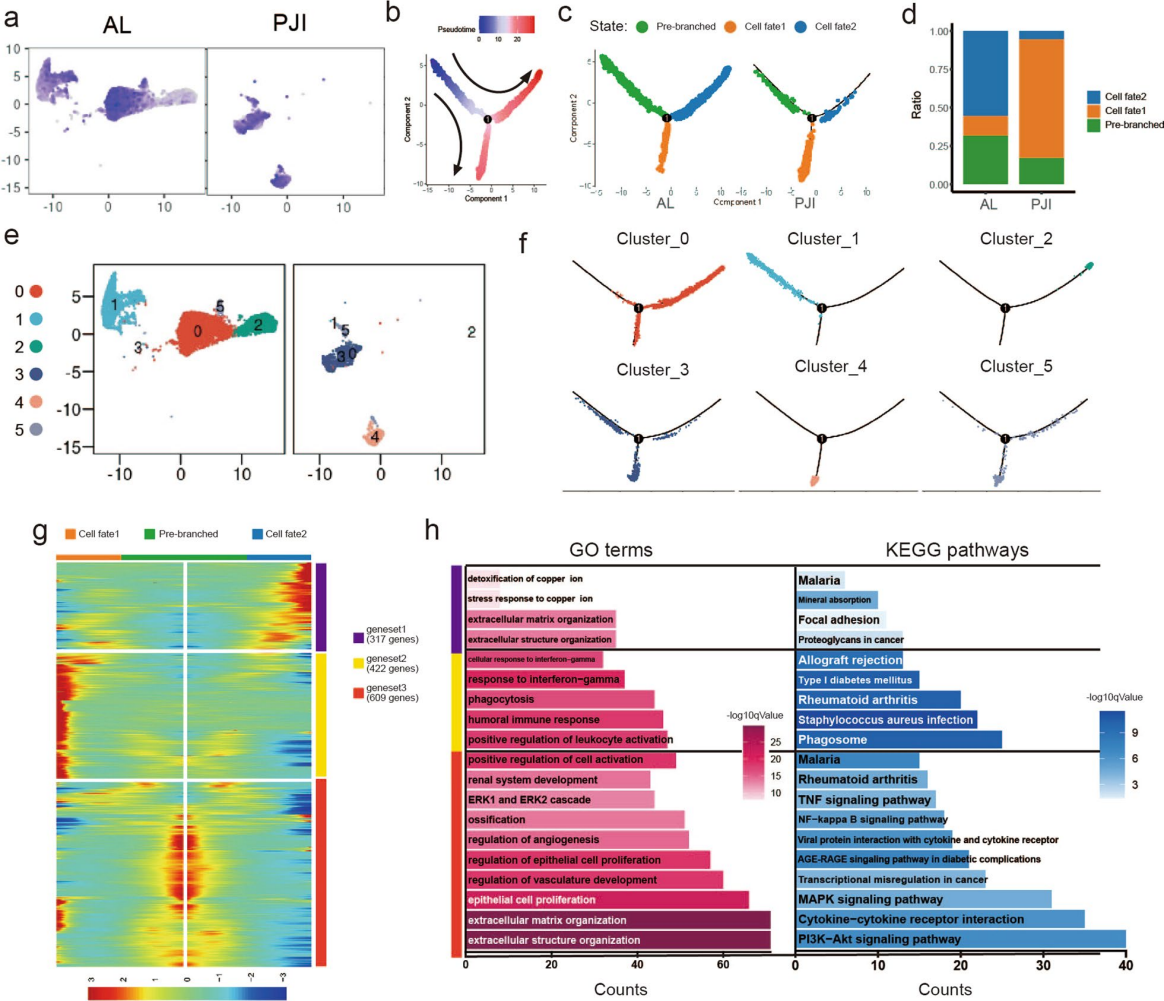
In-depth fibroblast trajectory analysis reveals a bipolar mode of differentiation. **a** UMAP visualization of the fibroblast, cells are colored according to *CTHRC1* expression level. **b** Pseudotime trajectory analysis shows fibroblasts differentiation, cells are colored by pseudotime. **c** Trajectory plots show fibroblasts differentiation in AL and PJI groups, cells are colored by cell states. **d** Stacked bar plot showing proportions of each cell state in AL and PJI groups. **e** UMAP visualization of the fibroblast, cells are colored according to subclusters*.* **f** Fibroblast subclusters are projected to pseudotime trajectory, cells are colored by subclusters. **g** Heatmap revealed pseudotime-dependent differentially expressed gene clusters for cell fate1, cell fate2, and pre-branched cells. **h** Bar plots show the enrichment result of gene ontology and KEGG pathway for each of the gene clusters identified in (**g**), both are colored with log10qvalues

By integrating the publicly available datasets with our in-house generated fibroblast expression profiles as illustrated in Fig. 4a, we divided all of the analyzed fibroblasts into seven meta-clusters (Fig. 4b). Among the seven meta-clusters, we noticed fibroblast meta-cluster2 overexpresses collagen-related genes (*COL1A, COL1A2, COL3A1, CTHRC1*) (Fig. 4c, Additional file 6: Fig. S5a–e). This *CTHRC1*^+^ fibroblast was observed in AL, PJI, periodontitis, and synovium of osteoarthritis and rheumatoid arthritis(Fig. 4d). Gene ontology (GO) analysis and GSEA enrichment both showed that the overexpressed genes in *CTHRC1*^+^ fibroblasts were enriched in ossification and collagen organization (Fig. 4e-f). Comparing the expression pattern of *CTHRC1* in PJI and AL samples, we found both groups were composed of a large portion of *CTHRC1* + fibroblasts (81.5% in AL vs 86.7% in PJI). Hence, we believe that *CTHRC1* + fibroblast is the major type of fibroblast in the human bone-implant interface. (Fig. 5a).

### Bipolar differentiation of fibroblast in the bone-implant interface

To further investigate fibroblast responses to infection in the bone-implant interface, we carried out a single-cell pseudotime trajectory analysis for fibroblasts in the AL and PJI groups, the result showed that fibroblasts could be classified into two continuous cell lineages: cell fate1/2 **(**Fig. 5b, c). The PJI group comprised many more cells that underwent fate1 than the AL group (77.48% vs. 12.82%), whereas the AL group mainly comprised cells in fate2 and pre-branched cells (Fig. 5d). Fibroblast subcluster1, 2, 4 were at pre-branched state, cell fate2, and cell fate1 respectively. Other subclusters were at intermediate state (Fig. 5e, f). We further classified pseudotime-dependent genes into 3 gene sets (geneset1–geneset3) according to their mode of expression (Fig. 5g, Additional file 7: Table S2). Geneset1 is composed of 317 genes and is responsible for cell fate2 regulation, GO term enrichment results showed its relation to extracellular matrix organization and stress response to copper ions (Fig. 5h). Geneset2 consists of 422 genes that contributed to fate1 differentiation. Its KEGG enrichment results were enriched in *Staphylococcus* infection and the phagosome pathway, GO term enrichment suggested its immune modulation functions such as positive regulation of leukocyte activation. Genset3 comprised 609 genes involved in extracellular structure organization, regulation of vascular development, epithelial cell proliferation, and ossification. Given the above findings, we termed cell fate1, cell fate2, and pre-branched cells as inflammatory, matrix-producing, and pre-branched fibroblasts, respectively.

### Identification of key driver transcription factors determining fibroblast differentiation

**Fig. 6:**
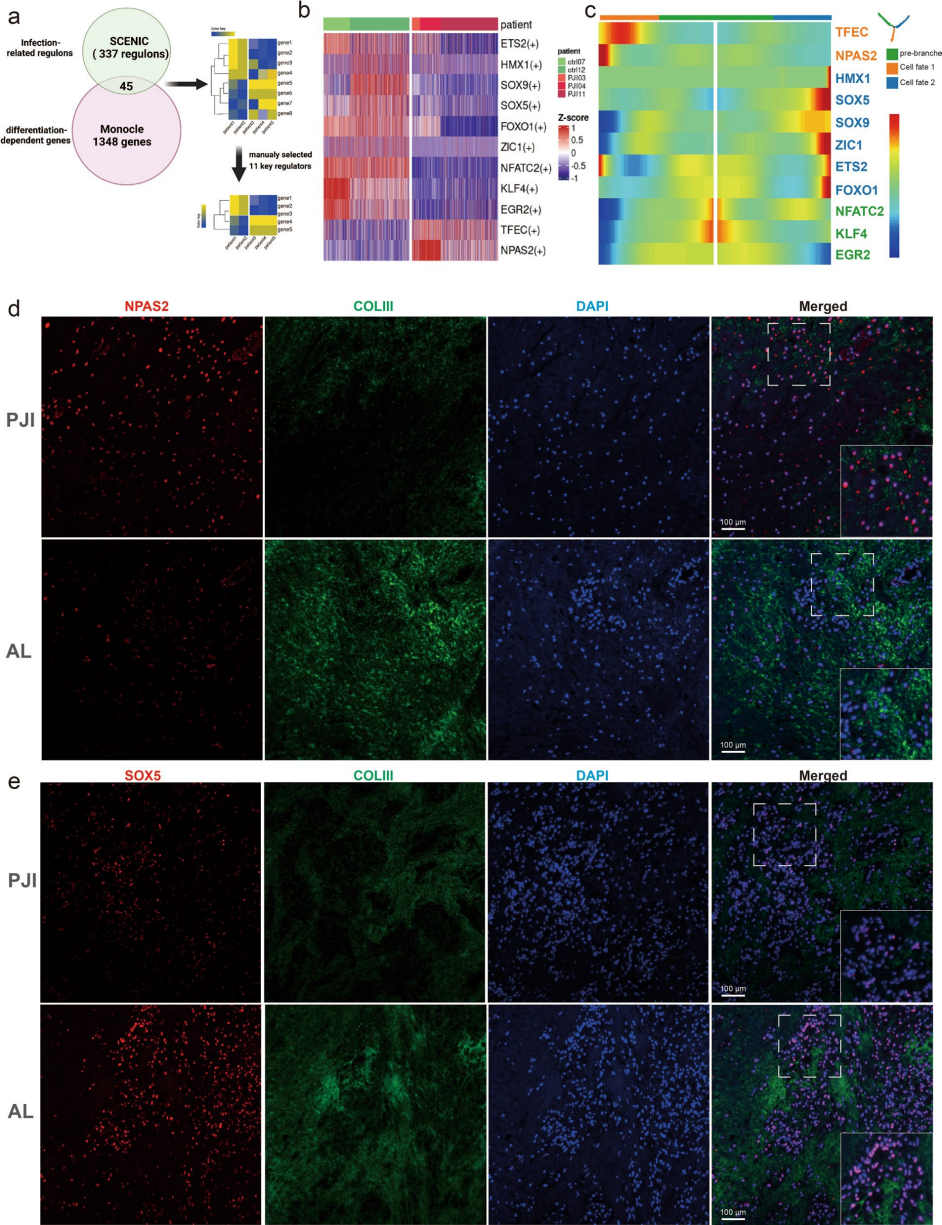
Identification for both pseudotime-dependent and disease-dependent regulators. **a** Workflow for identification of 11 key regulators. SCENIC analysis result in 337 regulons with significant differences between PJI and AL groups then were intersected with genes critical for fibroblast differentiation (1348 genes identified during pseudotime analysis), resulting in 45 transcription factors. We further removed 34 transcription regulators whose expression levels were not consistent among individuals with the same diagnosis. **b** SCENIC analysis result for the selected eleven regulons visualized with heatmap. **c** Heatmap shows the eleven transcription factor expression changes along pseudotime differentiation. Immunofluorescent assays display NPAS2 **d** SOX5 **e** expression in the AL and PJI groups, sections were labeled with anti-collagen III (green) which shows the distribution of collagen

To identify crucial regulators responsible for infection-induced fibroblast differentiation, we performed regulatory network analysis (SCENIC) for a total number of 388 regulons. The resultant 337 regulons with significant differences between PJI and AL groups were selected and intersected with genes critical for fibroblast differentiation (1348 genes identified during pseudotime analysis), thus resulting in 45 transcription factors. To exclude the effects of over-representation of a dataset due to high cell number contribution, we removed 34 transcription regulators whose expression levels were not consistent among individuals with the same diagnosis (Fig. 6a). The AUCell scores for each of the 11 regulons were shown in Fig. 6b, a distinct difference could be observed between PJI and AL. Furthermore, We found that inflammatory fibroblasts are strongly correlated with *TFEC* and *NPAS2*, whereas matrix-producing fibroblasts are mainly modulated by *HMX1, SOX5, SOX9, ZIC1, ETS2,* and *FOXO1*. The remaining TFs (*NFATC2, KLF4*, and *EGR2*) are responsible for cell differentiation in the pre-branched state (Fig. 6c). By selecting three representative TFs in each type of cell fates (NPAS2, SOX5, NFATC2), we verified the above-mentioned differences at the protein level using immunofluorescence (Fig. 6d, e, Additional file 8: Fig. S6). The results showed that NPAS2 was highly expressed in the PJI group while SOX5 and NFATC2 were mainly expressed in the AL group.

### Forskolin is a potential treatment strategy by targeting fibroblast differentiation

**Fig. 7:**
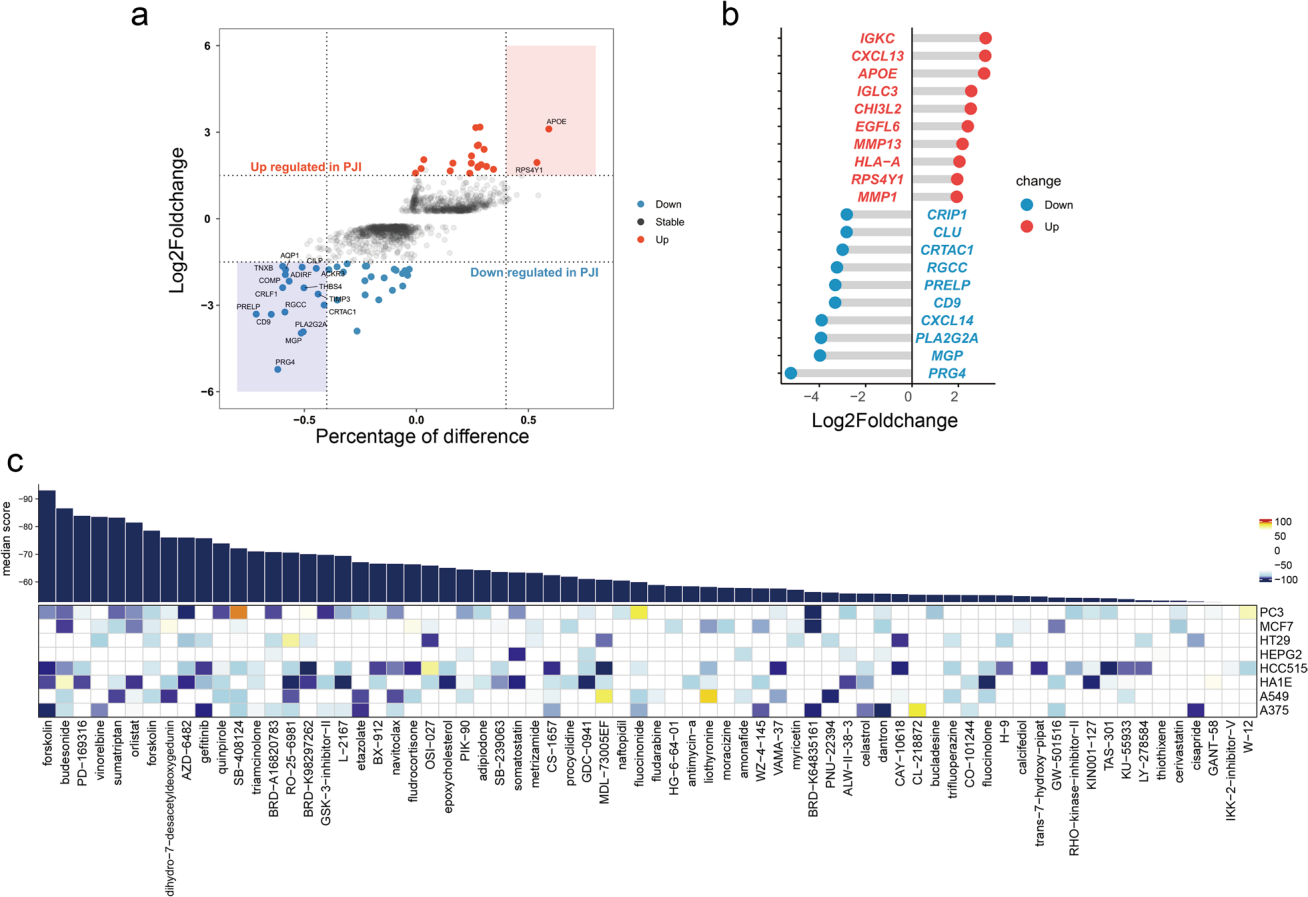
CMap analysis screening for potential fibroblast differentiation regulators. **a** Scatter plot showing differentially expressed genes between PJI and AL group. The most significant hits are highlighted with color rectangles. **b** Lolipop displays the top ten upregulated and downregulated genes in the PJI group. **c **The heatmap shows correlation scores for each compound (columns) when treated to a specific cell line (rows). The bar plot displays the average scores for each tested compound

To identify potential compounds for regulating fibroblast differentiation, we carried out a CMap analysis. Differentially expressed genes (DEGs) of fibroblasts in the PJI group were filtered by setting the adjusted p-value < 0.01 and log2Foldchange at ± 1.4. A total number of 24 genes were upregulated and 45 were downregulated (Fig. 7a, Additional file 9: Table S3). The most upregulated genes in the PJI group were *IGKC, CXCL13, APOE*, and *IGLC3* whereas the most downregulated genes were *PRG4*, *MGP*, *PLA2G2A*, and *CXCL14* (Fig. 7b). The DEGs were used as an input for CMap analysis, the top 70 compound hits are shown in Fig. 7c. Only forskolin, an adenylyl cyclase activator, has an estimated score < − 90.

## Discussion

Orthopedic implants have been used clinically for decades. Due to the existence of foreign materials, a variety of complications may be encountered following orthopedic procedures (Schwartz et al. 2020). This study focused on the most common complications after arthroplasty: PJI and AL. At present, research on the environment around medical implants is still in the exploratory stage. In 2021, Cherry et al. (2021) published a single-cell atlas regarding response to biomaterials by adopting the mouse volumetric muscle loss (VML) model. This is the first report depicting foreign body reactions using scRNA-seq technology. In contrast to their findings, B cells and plasma cells, which were observed in our cell atlas, were absent in the previous VML model. This discrepancy could be caused by a variety of factors: species (human/mouse), postoperative period (months/weeks), sampling site (bone/muscle-implant interface), and material (alloy/polycaprolactone). It is worth noting that, by comparing data from PJI04 and PJI03, we did not observe much difference in cell composition caused by implant material (PMMA v.s. titanium alloy coated with HA) and sampling site (knee v.s. hip). According to our observation, late chronic infections (PJI03 & PJI04) mainly consists of T_1 cells while early acute infection (PJI11) is composed of Myeloid cells and Fibroblast. Thus, we hypothesize the infection stage might contribute significantly to the cell heterogeneity rather than materials, coating and sampling site.

Through a meta-analysis of fibroblast transcriptome across various disease states, we identified that fibroblasts in the bone-implant interface were mainly composed of *CTHRC1*^+^ fibroblasts. It was not the first report of *CTHRC1*^+^ fibroblast, Adrián et.al. demonstrated it as a novel regulator of the healing scar process and a prospective target for myocardial infarction treatment (Ruiz-Villalba et al. 2020). The preferential occurrence of *CTHRC1*^+^ fibroblast in inflammatory bone-related diseases (AL, PJI, osteoarthritis, rheumatoid arthritis, and periodontitis) and its presence in the AL and PJI samples lead us to hypothesize its role in bone-implant osseointegration.

**Fig. 8:**
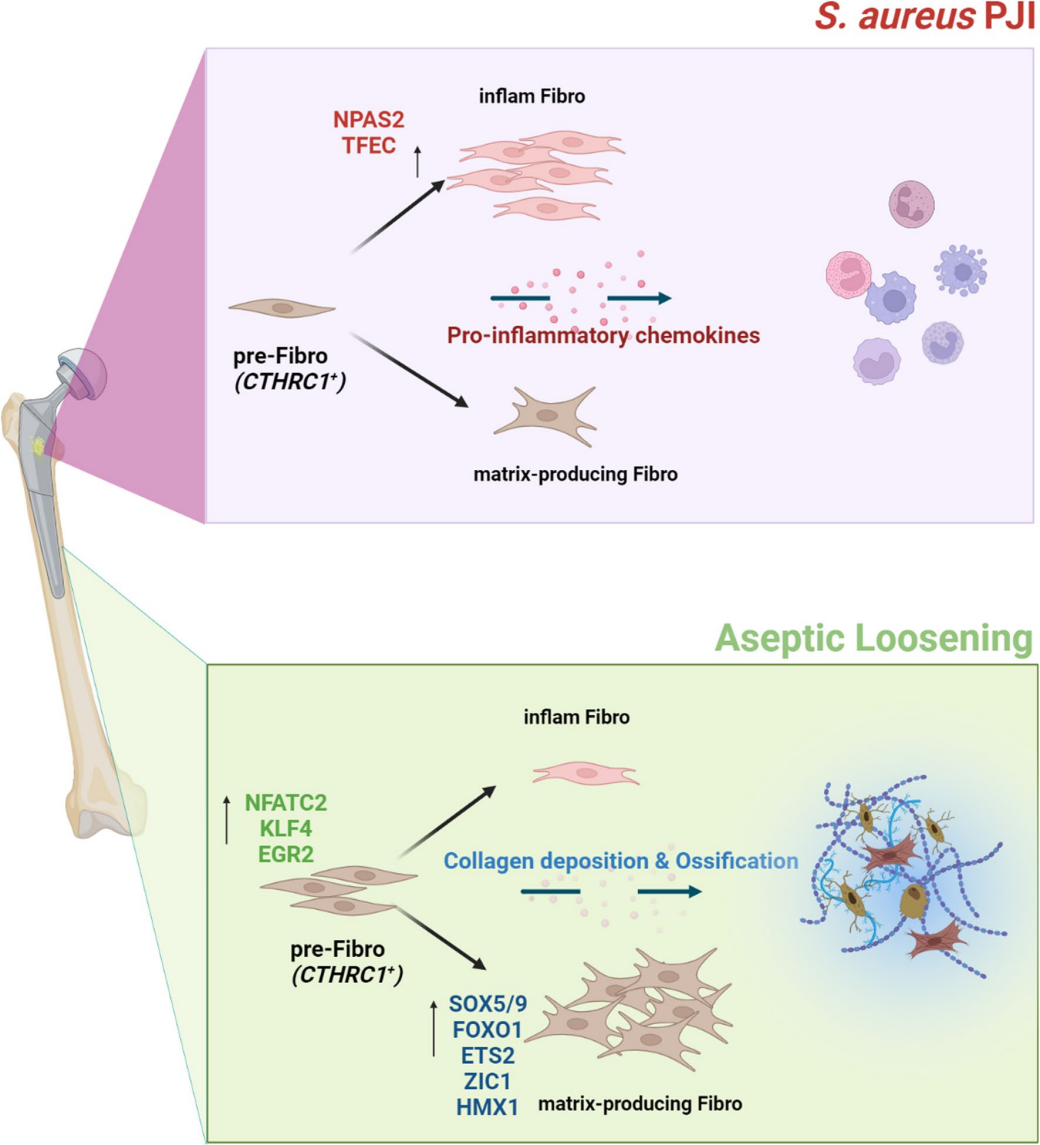
Illustration of the bipolar differentiation of fibroblast in the bone-implant interface. Periprosthetic joint infection with *S. aureus* could induce inflammatory fibroblast differentiation through up-regulation of *NPAS2* and *TFEC*, thus inducing immune cell migration and impairing the extracellular matrix deposition. An aseptic environment contains less inflammatory fibroblast and more matrix-producing fibroblast, resulting in more collagen deposition and promotion of ossification

It was reported that *CTHRC1*^+^ fibroblasts take part in the healing repair process through SOX9 and TGF-β signaling pathways, while our study indicates *S. aureus* infection could interfere with this process by modulating the fibroblast differentiation toward an inflammatory phenotype (Fig. 8). We observed downregulated *SOX5*/*SOX9* levels and upregulated levels of *TFEC*/*NPAS2* in the inflammatory fibroblast lineage. To our knowledge, *TFEC* is a MiT family transcription factor, its expression is highly restricted in macrophages with limited studies on its functions in fibroblasts (Kim et al. 2021). In contrast, *NPAS2* is a known transcription factor that regulates circadian rhythms, and it controls *CHI3L1* expression which has been reported by Brian et al. taking part in neuroinflammation (Lananna et al. 2020). The downregulated *SOX5* and *SOX9* are related to cartilage development and belong to the SOX family. It is noteworthy that the *SOX4* and *SOX11*-mediated TGF-β pathway was also found to be involved in the fibrotic process in the VML mouse model (Cherry et al. 2021). Combining our study with previous research, the SOX family could be critical in promoting healing repair and osteointegration, and therefore a worthwhile focus for future studies.

In this study, we noticed the inflammatory fibroblasts mainly presented in PJI samples but also existed, though at a relatively low proportion, in AL specimens. We speculated the activation of inflammatory fibroblasts in a sterile environment may contribute to osteolysis. The prevention of excessive inflammatory fibroblast differentiation might be a potential treatment for AL. We then found forskolin as a fibroblast differentiation-regulating compound. Forskolin is an adenosine acid cyclase agonist (C22H34O7) and has been applied in clinical practice with several diseases (e.g., glaucoma, hypertension, and asthma). The forskolin derivative (NKH477) has also been approved in Japan years ago for the treatment of heart failure (Ju et al. 2021). It can be a promising therapy once its function has been experimentally verified.

This research only studied PJI caused by *S. aureus* due to its high prevalence. However, other pathogens also cause a large portion of PJI. Apart from *S. aureus,* the other commonly isolated pathogen for PJI was CoNS (41%) including *S. epidermis (*~ *30%)* and *S. lugdunensis*(4%) (Patel 2023), It is reported that *S. lugdunensis* could cause an array of clinical infections like that of *S. aureus *(Cronin et al. 2022). However, *S. epidermis* (~ 30%) is less virulent and relies primarily on biofilm to survive in the host (Lu et al. 2022). Other less frequently isolated pathogens include *Streptococcus* species (14%), *Enterococcus* species (8%), and *Cutibacterium* species (8%). Although many differences exist in pathogenicity among pathogens, several clinical retrospective studies found the outcome of PJI was similar for different pathogens (Renz et al. 2022; Trobos et al. 2022). Therefore, we hope our research could also enrich the understanding of PJI caused by other pathogens.

At last, our study has some limitations. First, the research finding cannot reflect the conditions of the average population because of the small sample size. Secondly, our analysis did not distinguish between locations (knee/hip joints) of tissue retrieval. Although we did not observe significant differences in cell composition between samples from different locations, it is necessary to take this factor into account while analyzing. Lastly, most of our research findings are based on bioinformatic analysis which requires further validation using biological experiments. In addition, besides investigating the molecular changes between the AL and PJI group, studying the morphological characteristic through techniques such as SEM and TEM would also be interesting in our future studies.

## Conclusions

Our study found fibroblast in the bone-implant interface expresses a high level of *CTHRC1* and uncovered a bipolar mode of fibroblast differentiation. Most importantly, we identified key transcriptional regulators that determine the fate of fibroblast and demonstrated that *S. aureus* infection can alter fibroblast phenotype by upregulating *NPAS2* and *TFEC* transcriptional regulators. We suggested the differentially regulated TFs could lead to significant differences in extracellular structure between PJI and AL groups. Our study could provide deeper insight into the importance of fibroblast fate regulation on *S. aureus* orthopedic IAI.

## Materials and methods

### Study subjects

Five patients requiring joint prostheses revision were involved in this study. The criteria of the Musculoskeletal Infection Society were used to define PJI (3 cases) and aseptic failure (2 cases). All antibiotics were discontinued for at least two weeks before the surgery. Patients with *S.aureus* infection were first diagnosed with metagenomic next-generation sequencing (mNGS) on prosthetic sonicate fluid as described previously (He et al. 2021) and later confirmed by bacterial culture after surgery. Written informed consent was provided by all participants. All procedures performed on patient samples were following the ethical standards of the ethics committee of Shanghai Sixth People’s Hospital. The clinical characteristic of patients is summarized in Additional file 1: Table S1.

### Sample processing and single-cell suspension preparation

Each sample(~ 1cm^3^) at the bone-implant interface was collected during the revision surgery, cut into small pieces (~ 1mm^3^) and washed twice with HBSS containing 1% FBS and 2 mM EDTA. The tissues were then enzymatically digested in 4 mg/mL collagenase III, with constant shaking for 45 min in a water bath at 37 °C. The enzymatic hydrolyzate was then filtered through a 70 μm cell strainer and centrifuged at 300 g/5 min. After the supernatant was discarded, red blood cell lysis solution (#C3702, Beyotime) was used to resuspend the precipitates (#C3702, Beyotime) and incubated for 5 min, washed twice with HBSS and cell viability was assessed using trypan blue staining. Samples with > 90% viable cells were used for downstream processing.

### Single-cell RNA sequencing

Single-cell suspensions at concentrations of 800–1000 cells/μL were loaded onto a Chromium single-cell instrument (10 × Genomics, Pleasanton, CA, USA) for the generation of single-cell bead-in-emulsion. cDNA library construction was prepared using 10 × Chromium Single-cell 3' library kits according to the manufacturer’s protocol. Each sample was processed separately and all libraries were sequenced with Novaseq 6000 (Illumina, San Diego, CA).

### Data pre-processing and quality control

Raw sequenced data obtained from the previous process were demultiplexed and mapped to the human reference genome (GRCh38) using Cell Ranger (v5.0) for the generation of gene expression matrices. The resulting expression matrices were further processed for quality control individually in R (v4.1.2) using Seurat (v4.1.0) (Hao et al. 2021) with the following criteria: (1) Genes expressed in fewer than three cells were removed. (2) Cells possessing > 6000 unique feature counts were removed. (3) Cells with unique feature counts < 600 were removed to exclude low-quality cells or empty droplets. (4) Cells having > 25% mitochondrial counts. (5) Doublets identified by DoubletFinder (v2.0.3) (McGinnis et al. 2019) were also excluded (the doublet rate was set to 0.054).

### Normalization, integration, dimensionality reduction, unsupervised clustering, and cell-type annotation

Feature counts for each cell were normalized to and log-transformed using the “NormalizaData” function with the default method. Two thousand highly variable genes (HVGs) were identified with the “FindVariableFeatures” function. Then, expression matrices were integrated to correct for batch effect using the method provided by Stuart et al. (2019). The expression levels of HVGs were scaled before performing PCA in the variable gene space. Next, 30 principal components were used for subsequent clustering (resolution = 0.1) and UMAP dimensionality reduction. All the functions involved in this section are provided by the R package Seurat (v4.1.0).

### Marker genes/Differentially expressed genes (DEGs) detection

To obtain the marker genes in a cluster of interest, the expression in all other cells was set for comparison. We used Wilcoxon’s test for statistical analysis. Genes whose log fold change was > 0.5 and q-value < 0.05 with expression in > 30% of that cluster were defined as marker genes.

To obtain the DEG list between the two groups, we used the default Wilcoxon’s test implemented in the “FindMarkers” function from Seurat. DEGs were defined as genes whose log fold change was > 1.5 with a q-value (FDR) < 0.001.

### Gene set enrichment via AUCell algorithm

To explore the gene set enrichment scores between samples, well-studied gene sets from GO biological processes were selected and considered as input for AUCell (Aibar et al. 2017) quantification. The gene sets and their member genes could be obtained from the msigdb database and gene ontology database.

### Fibroblast pseudotime trajectory analysis

The pseudotime trajectory analysis was performed using the R package monocle2 (Qiu et al. 2017a, b; Trapnell et al. 2014) (v2.22.0). Two thousand highly variable genes were detected among fibroblasts by Seurat using the 'VariableFeatures' function for pseudotime trajectory building. Pseudotime states of single cells were determined using the 'orderCells' function with default parameters and projected onto a trajectory tree after dimensional reduction via 'DDRTree' method. The pseudotemporal dependent gene expression patterns were analyzed using the 'BEAM' function, genes were divided into three clusters (genesets1–3) according to their expression mode.

### Single-cell regulatory network inference and clustering (SCENIC)

We down-sampled the fibroblasts derived from the AL group to 1500 cells, in order to mitigate the effects of over-representation compared with those from the PJI group. The SCENIC analysis was performed with a standard workflow provided using pySCENIC (Sande et al. 2020). Briefly, the co-expression modules are first inferred with the GRNBoost2 program. Then, indirect targets are pruned using cis Target. Two databases containing DNA motif information were downloaded from the cis Target database and used during analysis (Database1: https://resources.aertslab.org/cistarget/databases/homo_sapiens/hg38/refseq_r80/mc9nr/gene_based/hg38__refseq-r80__10kb_up_and_down_tss.mc9nr.feather; Database2: https://resources.aertslab.org/cistarget/databases/homo_sapiens/hg38/refseq_r80/mc9nr/gene_based/hg38__refseq-r80__500bp_up_and_100bp_down_tss.mc9nr.feather). Last, the activity of these regulons was quantified via AUCell enrichment method.

### Inference of cell–cell communication

Cellchat (Jin et al. 1088) (v1.1.3) method was applied for cell–cell communication inference. All cell types in AL and PJI groups were preprocessed separately and merged into a single object according to the instructions from the developers. The differential strengths of cell interactions were determined using the "netVisual_diffInteraction" function and "netVisual_heatmap". PCA plots for the major sources and target cell types in each group were drawn using the "netAnalysis_signalingRole_scatter" function. The comparison of the overall information flow of each signaling pathway was plotted with the "rankNet" function. The dysfunctional ligand-receptor pairs were calculated by comparing the communication probabilities with the "netVisual_bubble" function.

### Fibroblast scRNA-seq meta-analysis

The meta-analysis procedure was done as described by Buechler et al. (2021), Briefly, publicly available scRNA-seq data were acquired by searching the GEO database. Detailed information on the datasets involved in this study was listed in Additional file 10: Table S4. All of the scRNA-seq data were downloaded and proceeded with a quality control process to remove doublet cells, empty droplets, and dead cells. Individual expression matrices were integrated and batch effects were removed using the widely used harmony method (Korsunsky et al. 2019). The subsequent analysis procedures including cell and marker genes identification were done as described above.

### Connectivity map (CMap) analysis

The connectivity map analysis (Lin et al. 2020) was conducted online. Differentially expressed genes in infected fibroblasts were compared with those of the aseptic group; (log2Foldchange ≥ 1.5) was considered as input for CMap analysis. The generated hit compounds were ranked according to their enrichment scores and the top 70 hit compounds were illustrated as a heatmap and barplot.

### Hematoxylin–eosin and Masson staining

Fresh tissues collected during the surgery were fixed with 10% formalin for over 24 h and embedded in paraffin. Tissue Sections (5 μm) were deparaffinized with xylene and washed with 100% ethanol, then rehydrated through ethanol gradients (95%, 80%, 70%). Hematoxylin–eosin staining was performed according to routine protocols. Briefly, sections were stained with hematoxylin solution for 10 min, immersed in 1% acid ethanol for 5 s, then washed with distilled water. After being stained with eosin solution for 3 min, the slides were washed with running water for 1 h. Masson staining was performed with the ready-to-use kit following the manufacturer’s instructions (G1340, Solarbio, Beijing, China).

### Immunofluorescence assay

Tissue sections were prepared as described in the previous section. After deparaffinization and rehydration through ethanol gradients (95%, 80%, 70%), sections underwent an antigen repair process in sodium citrate buffer using microwave thermal repair and endogenous peroxidase blocking in 0.3% hydrogen peroxide. After nonspecific reactions were blocked with 5% goat serum at 20℃ for 10 min. Sections were first incubated with anti-COLIII primary antibody (GB111629, Servicebio, Wuhan China) overnight at 4 °C, then incubated with horseradish peroxidase-labeled goat anti-rabbit secondary antibody, and fluorescent dye was ligated to it according to the manufacturer’s instructions (abs50012, Absin, Shanghai, China). After washing with PBST 3 times, sections were further incubated with the second primary antibody (anti-SOX5 (A6985, Abclonal, Wuhan, China), anti-NFATC2 (A3107, Abclonal), anti-NPAS2 (A16930, Abclonal)) at room temperature for 1 h. The procedures were repeated starting from the second antibody incubation. After all the procedures were completed, the slides were washed in PBST, counterstained with DAPI, and sealed with anti-fluorescence quenching sealing tablets. The stained tissue sections were examined using a Leica DMI8 microscope (Leica, Germany).

## Supplementary Information

**Additional file 1: Table S1.** Clinical characteristics for patients with PJI and aseptic loosening.

**Additional file 2: Figure S1. a**, **b** sequence quality analysis result of the number of features, total numbers of RNA, and the ratio of mitochondrial genes before (**a**) and after (**b**) quality control. **c** The balloon plot displays the count of each cell type in each individual. **d** The heatmap shows the GSEA enrichment result for each of the annotated cell clusters. GO:BP databases were used in this analysis.

**Additional file 3: Figure S2**. **a** UMAP dimensionality reduction result depicting the location of all types of cells from each individual. **b**–**d** UMAP dimensionality reduction result depicting the location of fibroblast (**b**), myeloid cells (**c**) and T_1 cells (**d**) from each individual

**Additional file 4: Figure S3.** Cell communication result for individual patients. **a**–**f** Heatmap shows reciprocal differential interaction strength among various patient pairs. Red indicates increased interaction strength and blue indicates decreased strength in PJI. **g–k** Scatter plot showing the incoming/outcoming signal strength of each cell type from individual patients.

**Additional file 5: Figure S4.** Fibroblasts, myeloid cells and T_1 cells GO term enrichment result. **a** GO term enrichment results for fibroblast genes upregulated (log2 foldchange > 1.0) in the PJI group.** b** GO term enrichment results for fibroblast genes downregulated (log2 foldchange < − 1.0) in the PJI group **c** GO term enrichment results for myeloid cell genes upregulated (log2 foldchange > 1.0) in the PJI group. **d** GO term enrichment results for myeloid cell genes downregulated (log2 foldchange < − 1.0) in the PJI group. **e** GO term enrichment results for T_1 cell genes upregulated (log2 foldchange > 1.0) in the PJI group. **f** GO term enrichment results for T_1 cell genes downregulated (log2 foldchange < − 1.0) in the PJI group.

**Additional file 6: Figure S5.** Visualization of marker genes in *CTHRC1*^+^ fibroblasts. (a-e) CTHRC1, COL1A1, COL1A2, COL3A1, and POSTN expression in CTHRC1, displayed with UMAP plot.

**Additional file 7: Table S2.** Gene clusters responsible for fibroblast differentiation in the perprosthetic environment.

**Additional file 8: Figure S6.** Immunofluorescent assay result for NFATC2. NFATC2 (red) was highly expressed in the AL group. CollagenIII was labeled with green.

**Additional file 9: Table S3.** Differentially expressed genes in fibroblast.

**Additional file 10: Table S4.** Detailed information for scRNA-seq samples for integration analysis.

## Data Availability

The single-cell expression matrix generated in this study has been deposited in the National Genomics Data Center (NGDC) of China (https://ngdc.cncb.ac.cn/) with Bioproject number: PRJCA009895. Other data involved in the current study are available upon reasonable request from the corresponding author.

## Abbreviations

IAI: Implant-associated infection
mNGS: Metagenomic next-generation sequencing
FBS: Fetal bovine serum
EDTA: Ethylene diamine tetraacetic acid
HBSS: Hank's balanced salt solution
HVGs: Highly variable genes
PCA: Primary component analysis
UMAP: Uniform manifold approximation and projection
GO: Gene ontology
SCENIC: Single-cell regulatory network inference and clustering
CMap: Connectivity map
PJI: Periprosthetic joint infection
AL: Aseptic loosening
