# Supplementary material for: Single-cell transcriptome reveals Staphylococcus aureus modulating fibroblast differentiation in the bone-implant interface
Source: Mol Med. 2023 Mar 16;29:35. doi: 10.1186/s10020-023-00632-7 (PMC10021980; doi:10.1186/s10020-023-00632-7)
Supplement: Supplementary file 1 — Additional file 1: Table S1. Clinical characteristics for patients with PJI and aseptic loosening. [file 10020_2023_632_MOESM1_ESM.docx]

**Additional file 1: Table S1. Clinical characteristics for patients with PJI and aseptic loosening**

| **Identity** | PJI03 | PJI04 | PJI11 | AL07 | AL12 |
| --- | --- | --- | --- | --- | --- |
| **Sex** | Female | Female | Male | Female | Female |
| **Age** | 53 | 75 | 60 | 64 | 75 |
| **Disease** | PJI (left hip) | PJI (right knee) | PJI (right hip) | AL (left hip) | AL (left hip) |
| **Pathogen** | S. aureus | S. aureus | S. aureus | None | None |
| **mNGS reads#** | 770 | 315 | 424 | None | None |
| **MRSA/MSSA** | MSSA | MSSA | MSSA | None | None |
| **Indication for primary joint replacement"** | Osteoarthritis | Osteoarthritis | Osteoarthritis | Osteoarthritis | Osteoarthritis |
| **Manufacturer** | LINK | PALACOS | LINK | CHUNLI | Smith&Nephew |
| **Tissue Source** | Acetabular shell | Spacer  (Tibia platform)r | Femoral Stem | Femoral Stem | Femoral Stem |
| **Material Type** | Titanium alloy | PMMA | Titanium alloy | Titanium alloy | Titanium alloy |
| **Coating** | Hydroxyapatite | / | Hydroxyapatite | Hydroxyapatite | Hydroxyapatite |

#: read counts for metagenome Next Generation Sequencing (mNGS), could reflect bacteria load to some extent.
