# Supplementary figures and images for: Single-cell transcriptome reveals Staphylococcus aureus modulating fibroblast differentiation in the bone-implant interface
Source: Mol Med. 2023 Mar 16;29:35. doi: 10.1186/s10020-023-00632-7 (PMC10021980; doi:10.1186/s10020-023-00632-7)

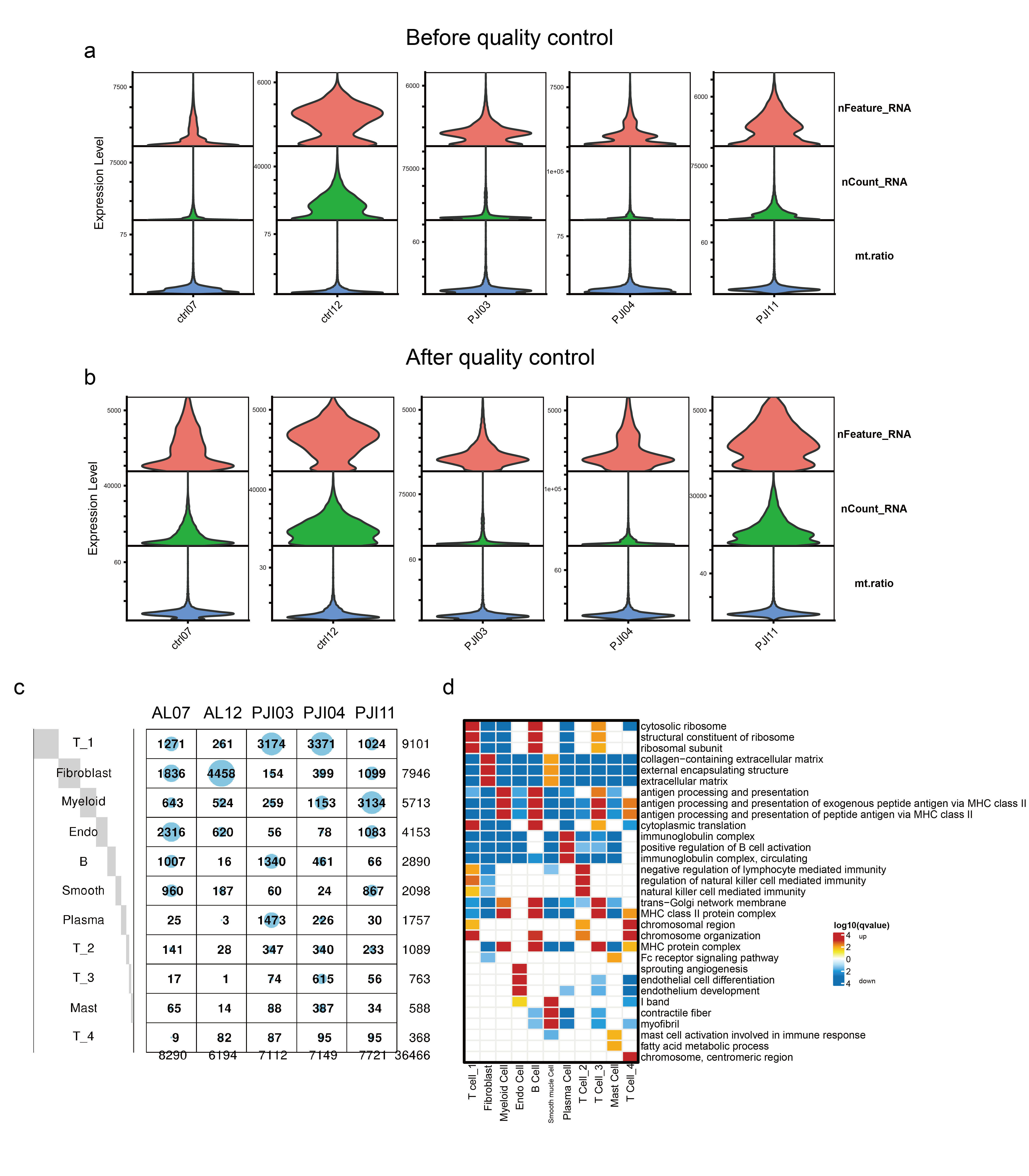

Supplement: Supplementary file 2 — Additional file 2: Figure S1. a, b sequence quality analysis result of the number of features, total numbers of RNA, and the ratio of mitochondrial genes before (a) and after (b) quality control. c The balloon plot displays the count of each cell type in each individual. d The heatmap shows the GSEA enrichment result for each of the annotated cell clusters. GO:BP databases were used in this analysis. [file 10020_2023_632_MOESM2_ESM.png]

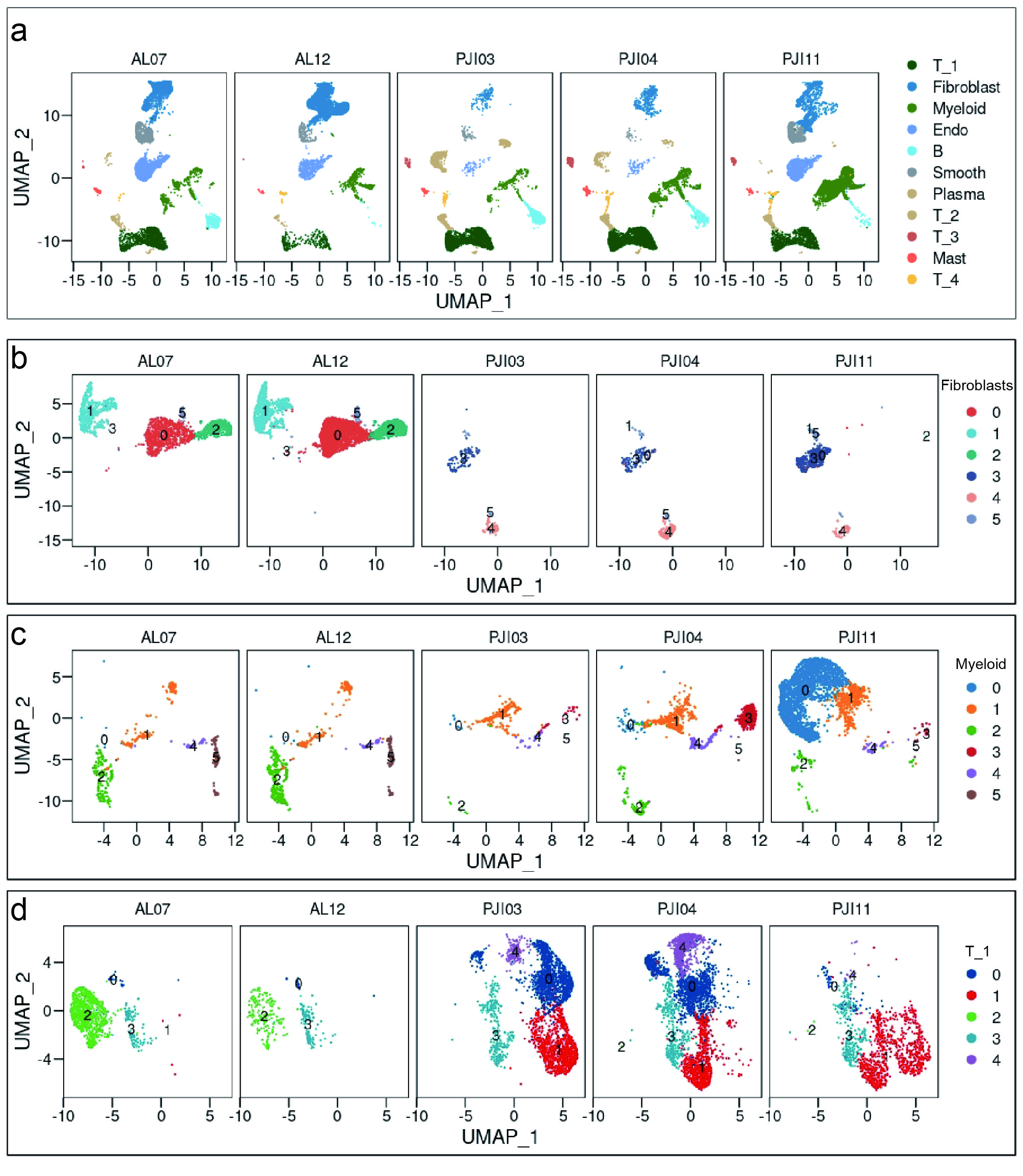

Supplement: Supplementary file 3 — Additional file 3: Figure S2. a UMAP dimensionality reduction result depicting the location of all types of cells from each individual. b–d UMAP dimensionality reduction result depicting the location of fibroblast (b), myeloid cells (c) and T_1 cells (d) from each individual [file 10020_2023_632_MOESM3_ESM.jpg]

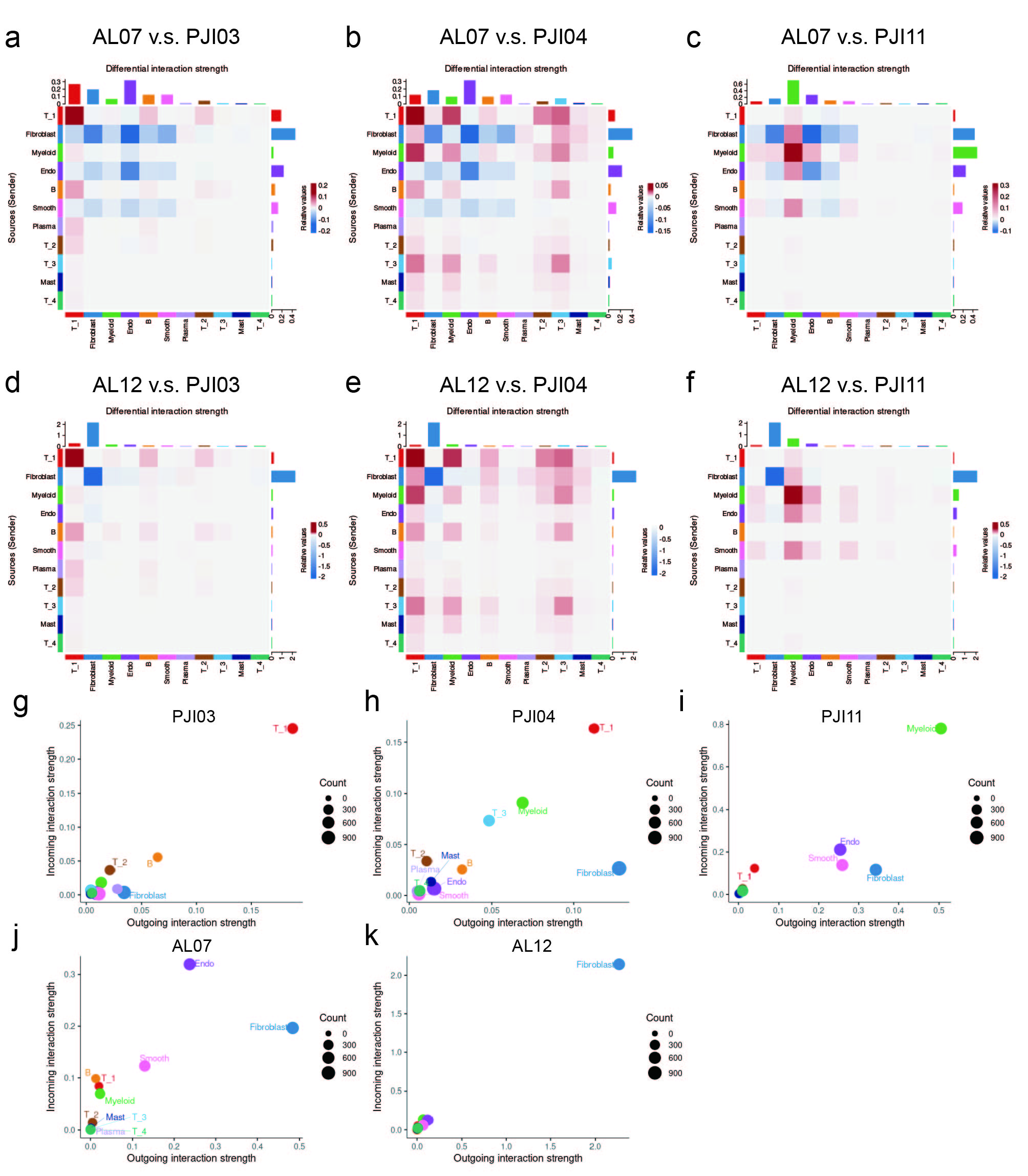

Supplement: Supplementary file 4 — Additional file 4: Figure S3. Cell communication result for individual patients. a–f Heatmap shows reciprocal differential interaction strength among various patient pairs. Red indicates increased interaction strength and blue indicates decreased strength in PJI. g–k Scatter plot showing the incoming/outcoming signal strength of each cell type from individual patients. [file 10020_2023_632_MOESM4_ESM.jpg]

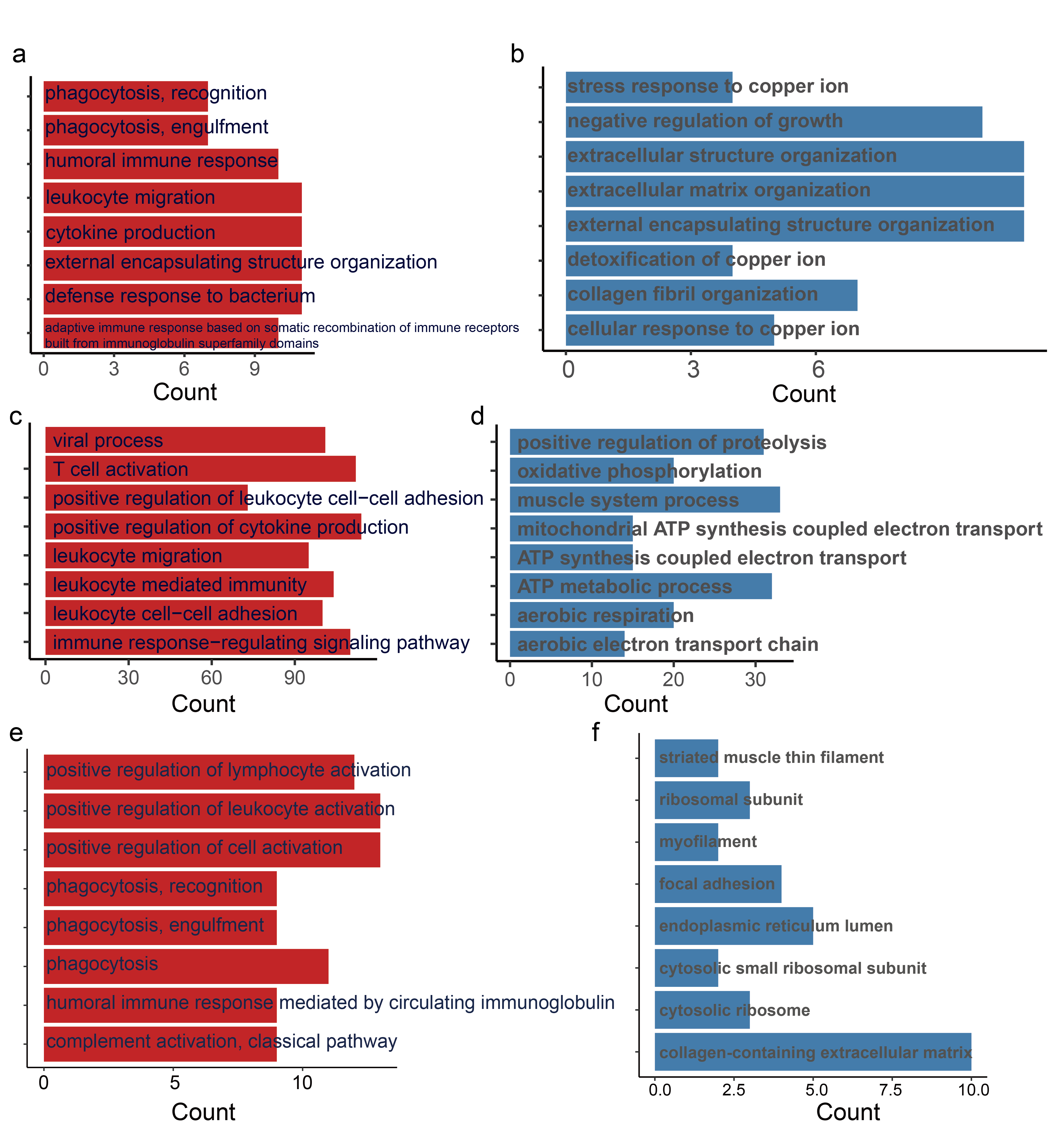

Supplement: Supplementary file 5 — Additional file 5: Figure S4. Fibroblasts, myeloid cells and T_1 cells GO term enrichment result. a GO term enrichment results for fibroblast genes upregulated (log2 foldchange > 1.0) in the PJI group. b GO term enrichment results for fibroblast genes downregulated (log2 foldchange < − 1.0) in the PJI group c GO term enrichment results for myeloid cell genes upregulated (log2 foldchange > 1.0) in the PJI group. d GO term enrichment results for myeloid cell genes downregulated (log2 foldchange < − 1.0) in the PJI group. e GO term enrichment results for T_1 cell genes upregulated (log2 foldchange > 1.0) in the PJI group. f GO term enrichment results for T_1 cell genes downregulated (log2 foldchange < − 1.0) in the PJI group. [file 10020_2023_632_MOESM5_ESM.png]

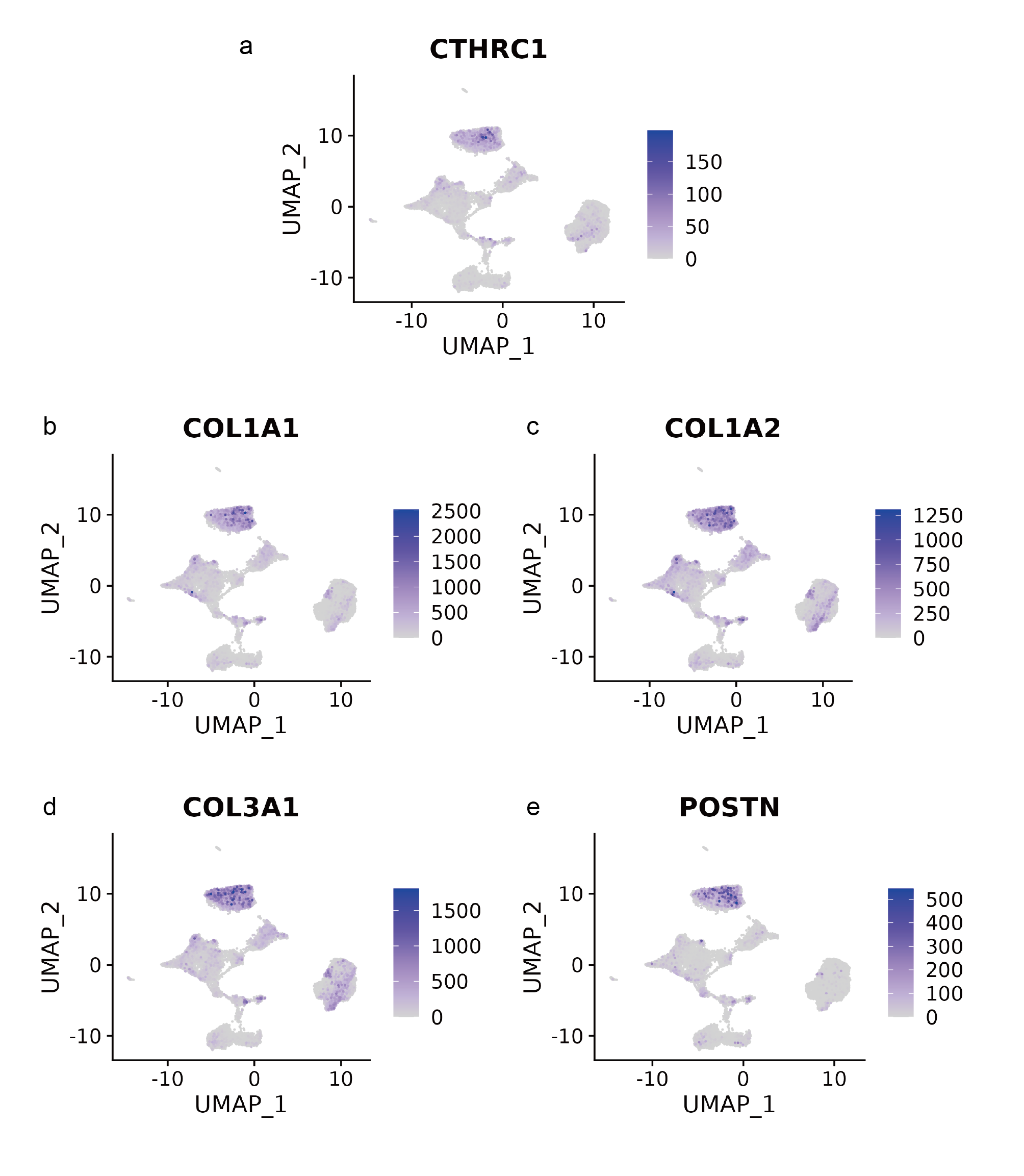

Supplement: Supplementary file 6 — Additional file 6: Figure S5. Visualization of marker genes in CTHRC1+ fibroblasts. (a-e) CTHRC1, COL1A1, COL1A2, COL3A1, and POSTN expression in CTHRC1, displayed with UMAP plot. [file 10020_2023_632_MOESM6_ESM.png]

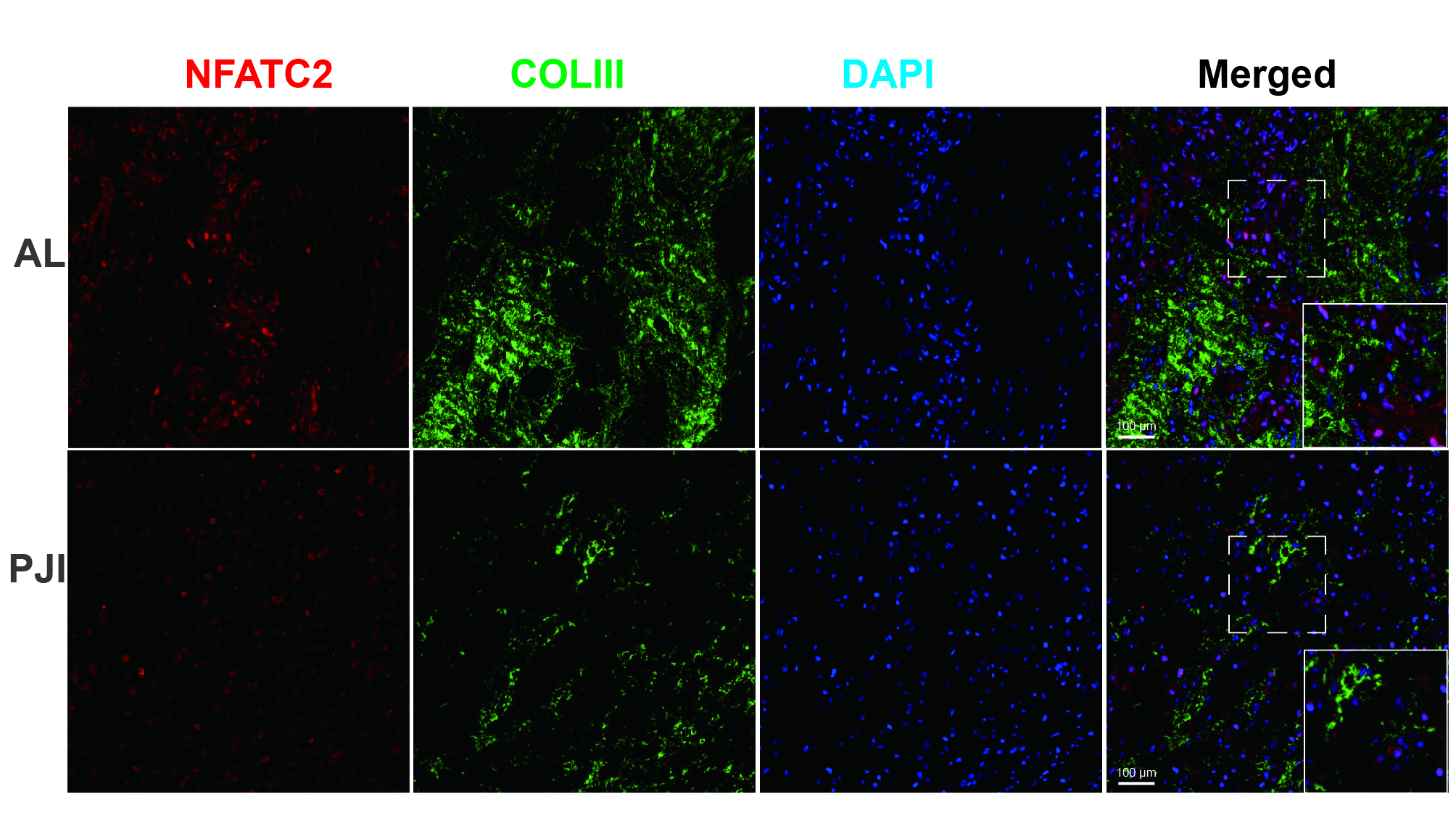

Supplement: Supplementary file 8 — Additional file 8: Figure S6. Immunofluorescent assay result for NFATC2. NFATC2 (red) was highly expressed in the AL group. CollagenIII was labeled with green. [file 10020_2023_632_MOESM8_ESM.jpg]
