# Supplementary material for: Single-cell transcriptome reveals Staphylococcus aureus modulating fibroblast differentiation in the bone-implant interface
Source: Mol Med. 2023 Mar 16;29:35. doi: 10.1186/s10020-023-00632-7 (PMC10021980; doi:10.1186/s10020-023-00632-7)
Supplement: Supplementary file 7 — Additional file 7: Table S2. Gene clusters responsible for fibroblast differentiation in the perprosthetic environment. [file 10020_2023_632_MOESM7_ESM.doc]

**Additional file 7: Table S2: gene clusters responsible for fibroblast differentiation in the perprosthetic environment.**

| **Gene Symbol** | **Gene_Clusters** | **p_value** | **q_value** |
| --- | --- | --- | --- |
| A2M | cluster_3 | 5.35E-15 | 9.30E-15 |
| ABCA1 | cluster_2 | 1.03E-40 | 2.67E-40 |
| ABCA10 | cluster_3 | 5.15E-60 | 1.67E-59 |
| ABCA6 | cluster_3 | 6.71E-95 | 2.99E-94 |
| ABCA8 | cluster_3 | 0 | 0 |
| ABCA9 | cluster_3 | 6.45E-75 | 2.37E-74 |
| ABCB11 | cluster_2 | 0.082955579 | 0.089504703 |
| ABCB5 | cluster_3 | 0.000580051 | 0.000706399 |
| ABCC9 | cluster_3 | 0 | 0 |
| ABHD5 | cluster_3 | 2.47E-56 | 7.73E-56 |
| ABL2 | cluster_3 | 0 | 0 |
| ABLIM1 | cluster_3 | 2.45E-110 | 1.22E-109 |
| AC002558.3 | cluster_3 | 0.601645131 | 0.611950924 |
| AC004540.2 | cluster_1 | 0.492929403 | 0.504052513 |
| AC004816.1 | cluster_1 | 0.493869465 | 0.504714076 |
| AC007262.2 | cluster_3 | 1.92E-12 | 3.17E-12 |
| AC008011.2 | cluster_1 | 0.000189894 | 0.000236099 |
| AC009779.2 | cluster_3 | 0.000103199 | 0.000130669 |
| AC010491.1 | cluster_3 | 0.128241316 | 0.136568674 |
| AC010624.5 | cluster_3 | 0.592221645 | 0.602722029 |
| AC010980.1 | cluster_2 | 6.29E-50 | 1.84E-49 |
| AC016074.2 | cluster_2 | 3.74E-05 | 4.83E-05 |
| AC016831.1 | cluster_3 | 2.33E-41 | 6.10E-41 |
| AC020916.1 | cluster_3 | 1.18E-18 | 2.22E-18 |
| AC023157.3 | cluster_3 | 0.028506171 | 0.031669436 |
| AC037198.1 | cluster_3 | 2.71E-38 | 6.82E-38 |
| AC079822.1 | cluster_2 | 0.114459147 | 0.122193832 |
| AC083967.1 | cluster_1 | 0.006252348 | 0.007201702 |
| AC084809.2 | cluster_1 | 0.262103806 | 0.274706484 |
| AC084871.1 | cluster_2 | 0.912294641 | 0.917087783 |
| AC087294.1 | cluster_2 | 0.000108476 | 0.000136747 |
| AC092484.1 | cluster_2 | 0.011168946 | 0.012711781 |
| AC103591.3 | cluster_1 | 3.21E-11 | 5.17E-11 |
| AC108134.2 | cluster_3 | 2.74E-14 | 4.69E-14 |
| AC112250.2 | cluster_1 | 0.07721261 | 0.08351766 |
| AC112721.1 | cluster_1 | 0.000170371 | 0.000211979 |
| AC114296.1 | cluster_3 | 0.025103657 | 0.028015877 |
| AC117453.1 | cluster_3 | 0.051968746 | 0.056675226 |
| ACAN | cluster_1 | 3.21E-05 | 4.17E-05 |
| ACKR1 | cluster_3 | 1.92E-09 | 2.92E-09 |
| ACKR3 | cluster_1 | 1.94E-265 | 2.01E-264 |
| ACKR4 | cluster_3 | 0.118796121 | 0.126745303 |
| ACOT7 | cluster_1 | 3.22E-20 | 6.22E-20 |
| ACP5 | cluster_1 | 3.54E-78 | 1.35E-77 |
| ACSL1 | cluster_3 | 3.36E-31 | 7.70E-31 |
| ACTA2 | cluster_2 | 0 | 0 |
| ACTG2 | cluster_2 | 5.46E-59 | 1.75E-58 |
| ACTN1 | cluster_2 | 1.95E-118 | 1.01E-117 |
| ADAM12 | cluster_2 | 4.68E-58 | 1.49E-57 |
| ADAM28 | cluster_3 | 0.100901098 | 0.108526978 |
| ADAMDEC1 | cluster_2 | 2.85E-20 | 5.51E-20 |
| ADAMTS1 | cluster_3 | 0 | 0 |
| ADAMTS12 | cluster_2 | 1.87E-23 | 3.79E-23 |
| ADAMTS14 | cluster_2 | 4.28E-63 | 1.43E-62 |
| ADAMTS15 | cluster_3 | 2.53E-40 | 6.51E-40 |
| ADAMTS18 | cluster_3 | 2.28E-11 | 3.68E-11 |
| ADAMTS4 | cluster_2 | 1.17E-14 | 2.02E-14 |
| ADAMTS5 | cluster_2 | 1.37E-131 | 7.55E-131 |
| ADAMTS6 | cluster_1 | 0.041059394 | 0.045149602 |
| ADAMTS9 | cluster_3 | 2.39E-06 | 3.29E-06 |
| ADAMTSL2 | cluster_3 | 1.12E-14 | 1.93E-14 |
| ADAMTSL4 | cluster_3 | 1.53E-71 | 5.46E-71 |
| ADARB1 | cluster_2 | 5.95E-44 | 1.61E-43 |
| ADCY3 | cluster_3 | 1.06E-21 | 2.10E-21 |
| ADCYAP1 | cluster_3 | 1 | 1 |
| ADGRD1 | cluster_3 | 1.16E-52 | 3.49E-52 |
| ADGRE2 | cluster_2 | 4.63E-43 | 1.24E-42 |
| ADGRF5 | cluster_2 | 0.532567747 | 0.543616871 |
| ADGRG2 | cluster_1 | 2.37E-16 | 4.23E-16 |
| ADGRL4 | cluster_3 | 7.16E-30 | 1.59E-29 |
| ADH1B | cluster_3 | 6.24E-14 | 1.06E-13 |
| ADH1C | cluster_3 | 6.79E-63 | 2.25E-62 |
| ADH4 | cluster_3 | 0.293841446 | 0.306477874 |
| ADIRF | cluster_1 | 8.69E-172 | 6.24E-171 |
| ADM | cluster_3 | 5.80E-05 | 7.42E-05 |
| ADRA1B | cluster_1 | 7.30E-05 | 9.30E-05 |
| ADRA2A | cluster_2 | 0 | 0 |
| AEBP1 | cluster_2 | 0 | 0 |
| AGT | cluster_3 | 0 | 0 |
| AHNAK2 | cluster_3 | 5.92E-82 | 2.35E-81 |
| AHR | cluster_3 | 1.45E-94 | 6.42E-94 |
| AIF1 | cluster_2 | 3.72E-57 | 1.17E-56 |
| AIF1L | cluster_3 | 1.56E-25 | 3.27E-25 |
| AIM2 | cluster_2 | 1.52E-207 | 1.26E-206 |
| AK1 | cluster_1 | 1.91E-45 | 5.25E-45 |
| AKAP12 | cluster_3 | 0 | 0 |
| AKR1C1 | cluster_1 | 2.15E-89 | 9.05E-89 |
| AKR1C2 | cluster_1 | 2.27E-102 | 1.05E-101 |
| AL139393.3 | cluster_3 | 8.78E-25 | 1.82E-24 |
| AL157373.2 | cluster_1 | 0.000105011 | 0.000132669 |
| AL157895.1 | cluster_1 | 0.000491202 | 0.000599468 |
| AL355312.4 | cluster_1 | 0.024769137 | 0.027660475 |
| AL355607.2 | cluster_1 | 4.96E-11 | 7.93E-11 |
| AL583785.1 | cluster_3 | 2.46E-24 | 5.04E-24 |
| AL590004.3 | cluster_3 | 0.001054625 | 0.001267316 |
| AL603756.1 | cluster_3 | 0.315954529 | 0.328348641 |
| AL627171.2 | cluster_3 | 1.46E-30 | 3.31E-30 |
| ALDH1A1 | cluster_3 | 6.93E-125 | 3.71E-124 |
| ALDH1A3 | cluster_3 | 8.87E-150 | 5.40E-149 |
| ALKAL2 | cluster_2 | 3.25E-08 | 4.70E-08 |
| ALOX5AP | cluster_2 | 7.70E-06 | 1.03E-05 |
| ALPL | cluster_3 | 8.97E-55 | 2.75E-54 |
| AMOTL2 | cluster_3 | 7.92E-33 | 1.87E-32 |
| AMTN | cluster_1 | 5.32E-42 | 1.41E-41 |
| ANGPT1 | cluster_3 | 8.26E-64 | 2.77E-63 |
| ANGPT2 | cluster_3 | 0.045223407 | 0.049538618 |
| ANGPTL1 | cluster_3 | 3.47E-140 | 2.01E-139 |
| ANGPTL4 | cluster_1 | 0.492464287 | 0.503876115 |
| ANGPTL5 | cluster_1 | 6.98E-31 | 1.59E-30 |
| ANGPTL7 | cluster_3 | 5.56E-05 | 7.11E-05 |
| ANK2 | cluster_3 | 0 | 0 |
| ANK3 | cluster_1 | 0.001842367 | 0.002180451 |
| ANKH | cluster_1 | 3.50E-38 | 8.80E-38 |
| ANKRD1 | cluster_3 | 2.52E-05 | 3.28E-05 |
| ANKRD37 | cluster_1 | 3.88E-24 | 7.92E-24 |
| ANO1 | cluster_1 | 0.000104663 | 0.000132327 |
| ANO5 | cluster_1 | 0.000164003 | 0.000204499 |
| ANOS1 | cluster_3 | 3.73E-14 | 6.38E-14 |
| ANTXR1 | cluster_2 | 8.67E-11 | 1.38E-10 |
| AOC2 | cluster_1 | 0.000109009 | 0.000137318 |
| AOX1 | cluster_3 | 3.46E-160 | 2.31E-159 |
| APBB1IP | cluster_3 | 1.34E-05 | 1.78E-05 |
| APCDD1 | cluster_2 | 5.31E-05 | 6.81E-05 |
| APCDD1L | cluster_1 | 3.71E-08 | 5.36E-08 |
| APCDD1L-DT | cluster_1 | 5.40E-13 | 9.04E-13 |
| APLNR | cluster_3 | 1.30E-07 | 1.85E-07 |
| APOBEC3A | cluster_2 | 2.66E-06 | 3.65E-06 |
| APOC1 | cluster_2 | 2.00E-19 | 3.81E-19 |
| APOD | cluster_3 | 0 | 0 |
| APOE | cluster_2 | 0 | 0 |
| APOLD1 | cluster_3 | 2.05E-11 | 3.32E-11 |
| AQP1 | cluster_1 | 8.78E-185 | 6.69E-184 |
| AQP4 | cluster_3 | 0.018095471 | 0.020352972 |
| AR | cluster_3 | 4.40E-47 | 1.24E-46 |
| ARC | cluster_3 | 1.00E-27 | 2.16E-27 |
| AREG | cluster_3 | 7.48E-15 | 1.30E-14 |
| ARG1 | cluster_3 | 0.878776391 | 0.884943243 |
| ARHGAP15 | cluster_3 | 0.000842852 | 0.001014959 |
| ARHGAP20 | cluster_3 | 8.49E-93 | 3.67E-92 |
| ARHGAP28 | cluster_2 | 1.33E-24 | 2.75E-24 |
| ARHGAP29 | cluster_3 | 5.72E-63 | 1.90E-62 |
| ARHGDIB | cluster_3 | 7.20E-14 | 1.22E-13 |
| ARHGEF28 | cluster_2 | 5.01E-16 | 8.92E-16 |
| ARID5B | cluster_3 | 2.76E-308 | 3.44E-307 |
| ARL4C | cluster_2 | 1.95E-14 | 3.35E-14 |
| ASPN | cluster_1 | 1.56E-48 | 4.46E-48 |
| ATF3 | cluster_3 | 0 | 0 |
| ATP1B1 | cluster_1 | 1.35E-25 | 2.83E-25 |
| ATP2B1 | cluster_2 | 0.019861961 | 0.022281627 |
| ATP2B1-AS1 | cluster_2 | 0.000130906 | 0.00016418 |
| ATP6V0D2 | cluster_1 | 0.011670751 | 0.013265369 |
| AUXG01000058.1 | cluster_3 | 0.000154606 | 0.000192921 |
| AVPR1A | cluster_3 | 0.001892439 | 0.002235103 |
| B3GNT2 | cluster_2 | 4.65E-25 | 9.68E-25 |
| B4GALT1 | cluster_3 | 6.07E-103 | 2.85E-102 |
| BAALC | cluster_2 | 9.88E-10 | 1.52E-09 |
| BAG3 | cluster_3 | 7.37E-150 | 4.50E-149 |
| BAMBI | cluster_1 | 0.039411259 | 0.043420466 |
| BARX1 | cluster_2 | 2.18E-72 | 7.85E-72 |
| BCAM | cluster_3 | 0.000203881 | 0.000253124 |
| BCAT1 | cluster_1 | 1.28590139559775e-316 | 1.67751681837495e-315 |
| BCHE | cluster_3 | 1.69E-06 | 2.33E-06 |
| BCL11A | cluster_2 | 6.02E-27 | 1.28E-26 |
| BCL11B | cluster_2 | 6.92E-11 | 1.10E-10 |
| BCL2A1 | cluster_1 | 2.29E-09 | 3.47E-09 |
| BDKRB1 | cluster_1 | 1.01E-15 | 1.79E-15 |
| BDKRB2 | cluster_3 | 1.05E-49 | 3.05E-49 |
| BDNF | cluster_3 | 1.99E-05 | 2.61E-05 |
| BEX1 | cluster_1 | 0.208175366 | 0.219252587 |
| BGLAP | cluster_1 | 1.32E-19 | 2.52E-19 |
| BIRC3 | cluster_2 | 7.55E-13 | 1.26E-12 |
| BIRC5 | cluster_2 | 2.04E-23 | 4.14E-23 |
| BIRC7 | cluster_1 | 0.079872967 | 0.086232758 |
| BMP1 | cluster_2 | 3.08E-169 | 2.16E-168 |
| BMP2 | cluster_3 | 0.006667336 | 0.007674567 |
| BMP4 | cluster_1 | 1.93E-71 | 6.87E-71 |
| BMP5 | cluster_3 | 7.66E-120 | 4.01E-119 |
| BMP8A | cluster_2 | 7.19E-69 | 2.49E-68 |
| BMP8B | cluster_2 | 2.23E-55 | 6.88E-55 |
| BMPER | cluster_3 | 4.76E-75 | 1.76E-74 |
| BNIP3 | cluster_1 | 5.10E-24 | 1.04E-23 |
| BOC | cluster_3 | 2.12E-304 | 2.55E-303 |
| BPIFB1 | cluster_3 | 0.088886049 | 0.095843316 |
| BPIFB4 | cluster_3 | 8.78E-80 | 3.40E-79 |
| BRINP1 | cluster_3 | 0.012889627 | 0.01462183 |
| BST2 | cluster_2 | 3.44E-104 | 1.63E-103 |
| BTBD3 | cluster_3 | 9.98E-34 | 2.39E-33 |
| BTG2 | cluster_3 | 0 | 0 |
| C10orf105 | cluster_1 | 0.137055356 | 0.145594894 |
| C11orf96 | cluster_1 | 5.75E-123 | 3.03E-122 |
| C12orf75 | cluster_1 | 5.40E-210 | 4.53E-209 |
| C15orf48 | cluster_2 | 0 | 0 |
| C16orf89 | cluster_3 | 2.61E-278 | 2.96E-277 |
| C1orf162 | cluster_2 | 5.19E-11 | 8.28E-11 |
| C1QA | cluster_2 | 9.16E-44 | 2.47E-43 |
| C1QB | cluster_2 | 4.71E-29 | 1.04E-28 |
| C1QC | cluster_2 | 9.26E-18 | 1.70E-17 |
| C1QL1 | cluster_1 | 7.65E-06 | 1.03E-05 |
| C1QTNF2 | cluster_1 | 1.35E-19 | 2.57E-19 |
| C1QTNF3 | cluster_1 | 3.29E-79 | 1.26E-78 |
| C1QTNF4 | cluster_3 | 1.41E-57 | 4.46E-57 |
| C1QTNF6 | cluster_2 | 4.37E-56 | 1.36E-55 |
| C1QTNF7 | cluster_1 | 4.55E-05 | 5.84E-05 |
| C2CD4B | cluster_3 | 0.027924918 | 0.031063765 |
| C2orf66 | cluster_3 | 4.69E-07 | 6.57E-07 |
| C3 | cluster_3 | 0 | 0 |
| C3orf80 | cluster_1 | 2.49E-39 | 6.31E-39 |
| C5AR1 | cluster_2 | 9.96E-11 | 1.58E-10 |
| C5AR2 | cluster_1 | 3.25E-10 | 5.09E-10 |
| C5orf46 | cluster_2 | 4.49E-38 | 1.13E-37 |
| C6 | cluster_3 | 3.30E-11 | 5.30E-11 |
| C7 | cluster_3 | 3.70797391302013e-314 | 4.72972672461558e-313 |
| CA12 | cluster_2 | 0 | 0 |
| CA2 | cluster_3 | 1.12E-26 | 2.39E-26 |
| CA9 | cluster_1 | 2.44E-05 | 3.19E-05 |
| CAB39L | cluster_3 | 3.36E-86 | 1.38E-85 |
| CACNA1H | cluster_2 | 1 | 1 |
| CADM1 | cluster_1 | 5.04E-07 | 7.05E-07 |
| CADM3 | cluster_2 | 1.33E-26 | 2.82E-26 |
| CALB2 | cluster_1 | 0.103359738 | 0.110963509 |
| CALCRL | cluster_3 | 1.51E-39 | 3.85E-39 |
| CALD1 | cluster_2 | 3.76E-10 | 5.85E-10 |
| CAMK2N1 | cluster_3 | 1.61E-54 | 4.93E-54 |
| CAPG | cluster_1 | 7.81E-141 | 4.59E-140 |
| CAPN6 | cluster_3 | 2.66E-35 | 6.45E-35 |
| CAPS | cluster_1 | 2.17E-49 | 6.28E-49 |
| CARMN | cluster_2 | 6.74E-53 | 2.04E-52 |
| CASQ2 | cluster_3 | 2.25E-05 | 2.95E-05 |
| CAV1 | cluster_1 | 4.46E-64 | 1.50E-63 |
| CAVIN2 | cluster_3 | 3.56E-31 | 8.14E-31 |
| CBLN4 | cluster_3 | 3.46E-05 | 4.49E-05 |
| CBR3 | cluster_1 | 0.255078814 | 0.267506527 |
| CCDC102B | cluster_2 | 1.04E-60 | 3.37E-60 |
| CCDC151 | cluster_1 | 3.34E-11 | 5.37E-11 |
| CCDC170 | cluster_3 | 3.16E-51 | 9.41E-51 |
| CCDC3 | cluster_3 | 1.35E-09 | 2.06E-09 |
| CCDC71L | cluster_3 | 1.38E-49 | 4.02E-49 |
| CCDC80 | cluster_3 | 0 | 0 |
| CCL11 | cluster_2 | 4.70E-48 | 1.33E-47 |
| CCL13 | cluster_3 | 1.37E-105 | 6.58E-105 |
| CCL14 | cluster_3 | 1.89E-20 | 3.67E-20 |
| CCL18 | cluster_2 | 9.40E-07 | 1.31E-06 |
| CCL19 | cluster_3 | 9.04E-70 | 3.18E-69 |
| CCL2 | cluster_3 | 3.04E-47 | 8.61E-47 |
| CCL20 | cluster_2 | 7.84E-31 | 1.78E-30 |
| CCL26 | cluster_3 | 1.07E-09 | 1.65E-09 |
| CCL3 | cluster_2 | 2.11E-42 | 5.60E-42 |
| CCL3L1 | cluster_2 | 5.05E-222 | 4.39E-221 |
| CCL4 | cluster_2 | 4.74E-155 | 3.05E-154 |
| CCL4L2 | cluster_2 | 4.25E-65 | 1.45E-64 |
| CCL5 | cluster_1 | 5.62E-164 | 3.87E-163 |
| CCL7 | cluster_2 | 1.58E-10 | 2.49E-10 |
| CCL8 | cluster_3 | 0.000212579 | 0.000263543 |
| CCN1 | cluster_3 | 0 | 0 |
| CCN2 | cluster_3 | 2.85E-147 | 1.72E-146 |
| CCN3 | cluster_3 | 0 | 0 |
| CCN4 | cluster_1 | 1.63E-204 | 1.33E-203 |
| CCN5 | cluster_3 | 4.45E-09 | 6.68E-09 |
| CCN6 | cluster_2 | 7.41E-32 | 1.73E-31 |
| CCND1 | cluster_1 | 5.94E-198 | 4.74E-197 |
| CCNL1 | cluster_3 | 7.78E-196 | 6.15E-195 |
| CCR7 | cluster_3 | 4.24E-05 | 5.46E-05 |
| CD14 | cluster_2 | 6.22E-37 | 1.55E-36 |
| CD151 | cluster_1 | 2.80E-84 | 1.12E-83 |
| CD163 | cluster_2 | 3.56E-26 | 7.50E-26 |
| CD177 | cluster_3 | 2.99E-285 | 3.48E-284 |
| CD1C | cluster_2 | 0.009913304 | 0.011335132 |
| CD2 | cluster_2 | 3.05E-15 | 5.35E-15 |
| CD200 | cluster_2 | 1.49E-07 | 2.10E-07 |
| CD200R1 | cluster_3 | 1.06E-06 | 1.47E-06 |
| CD24 | cluster_1 | 3.82E-08 | 5.50E-08 |
| CD247 | cluster_2 | 3.46E-05 | 4.49E-05 |
| CD248 | cluster_3 | 4.72E-18 | 8.70E-18 |
| CD300E | cluster_2 | 1.33E-09 | 2.04E-09 |
| CD302 | cluster_3 | 1.76E-139 | 1.01E-138 |
| CD34 | cluster_3 | 4.06E-163 | 2.77E-162 |
| CD36 | cluster_3 | 1.67E-05 | 2.20E-05 |
| CD37 | cluster_2 | 0.000650914 | 0.000789903 |
| CD3D | cluster_2 | 3.33E-18 | 6.16E-18 |
| CD3E | cluster_2 | 6.57E-06 | 8.84E-06 |
| CD3G | cluster_2 | 4.57E-08 | 6.55E-08 |
| CD48 | cluster_2 | 1.48E-07 | 2.10E-07 |
| CD52 | cluster_2 | 6.91E-23 | 1.39E-22 |
| CD53 | cluster_2 | 1.43E-11 | 2.33E-11 |
| CD55 | cluster_1 | 5.75E-80 | 2.23E-79 |
| CD68 | cluster_1 | 1.46E-48 | 4.18E-48 |
| CD69 | cluster_2 | 5.86E-21 | 1.15E-20 |
| CD70 | cluster_1 | 1.62E-10 | 2.55E-10 |
| CD74 | cluster_2 | 9.15E-25 | 1.89E-24 |
| CD79A | cluster_3 | 1.43E-05 | 1.89E-05 |
| CD82 | cluster_1 | 0 | 0 |
| CD83 | cluster_3 | 3.59E-06 | 4.88E-06 |
| CD84 | cluster_2 | 4.60E-09 | 6.90E-09 |
| CD86 | cluster_2 | 1.75E-11 | 2.85E-11 |
| CD9 | cluster_1 | 7.82E-277 | 8.69E-276 |
| CD93 | cluster_2 | 0.105129256 | 0.112792884 |
| CD96 | cluster_2 | 0.000260719 | 0.000321604 |
| CDA | cluster_1 | 0.229990051 | 0.241636893 |
| CDCP1 | cluster_2 | 1.35E-40 | 3.50E-40 |
| CDH19 | cluster_1 | 0.136514856 | 0.145110236 |
| CDH2 | cluster_1 | 4.94E-19 | 9.33E-19 |
| CDH5 | cluster_3 | 0.012717477 | 0.014436055 |
| CDH6 | cluster_3 | 2.38E-06 | 3.27E-06 |
| CDK1 | cluster_2 | 0.001405387 | 0.001673635 |
| CDK2AP2 | cluster_1 | 1.57E-15 | 2.76E-15 |
| CDK5RAP2 | cluster_3 | 4.94E-17 | 8.94E-17 |
| CDKN1A | cluster_3 | 8.42E-93 | 3.65E-92 |
| CDKN1C | cluster_3 | 3.84E-286 | 4.50E-285 |
| CDKN2A | cluster_1 | 6.78E-89 | 2.83E-88 |
| CDKN3 | cluster_3 | 1.28E-30 | 2.90E-30 |
| CDO1 | cluster_1 | 1.10E-175 | 8.03E-175 |
| CDX1 | cluster_1 | 6.62E-06 | 8.89E-06 |
| CEBPA | cluster_3 | 9.12E-42 | 2.40E-41 |
| CELF2 | cluster_3 | 1.58E-140 | 9.18E-140 |
| CELF4 | cluster_1 | 0.296787523 | 0.309363266 |
| CEMIP | cluster_1 | 4.73E-45 | 1.30E-44 |
| CEMIP2 | cluster_3 | 0.002855819 | 0.003347665 |
| CEND1 | cluster_2 | 0.001567634 | 0.00186556 |
| CENPF | cluster_2 | 3.63E-06 | 4.93E-06 |
| CES1 | cluster_3 | 4.78E-57 | 1.50E-56 |
| CETP | cluster_3 | 0.30377813 | 0.31630756 |
| CFAP69 | cluster_3 | 3.74E-40 | 9.59E-40 |
| CFB | cluster_3 | 1.78E-41 | 4.66E-41 |
| CFD | cluster_3 | 0 | 0 |
| CFH | cluster_3 | 3.01E-160 | 2.02E-159 |
| CFHR1 | cluster_3 | 2.46E-09 | 3.72E-09 |
| CFP | cluster_2 | 0.102932762 | 0.110643081 |
| CH25H | cluster_3 | 2.68E-30 | 6.03E-30 |
| CHAD | cluster_3 | 7.63E-34 | 1.83E-33 |
| CHCHD10 | cluster_1 | 9.59E-93 | 4.14E-92 |
| CHD1 | cluster_3 | 1.92E-177 | 1.42E-176 |
| CHD7 | cluster_3 | 1.60E-10 | 2.53E-10 |
| CHI3L1 | cluster_3 | 0 | 0 |
| CHI3L2 | cluster_1 | 0 | 0 |
| CHL1 | cluster_3 | 1.67E-67 | 5.74E-67 |
| CHMP1B | cluster_3 | 0 | 0 |
| CHN1 | cluster_2 | 6.74E-50 | 1.97E-49 |
| CHODL | cluster_3 | 1.09E-10 | 1.73E-10 |
| CHRDL1 | cluster_3 | 0 | 0 |
| CHRDL2 | cluster_3 | 1.05E-60 | 3.42E-60 |
| CILP | cluster_1 | 0 | 0 |
| CILP2 | cluster_3 | 2.45E-82 | 9.72E-82 |
| CIT | cluster_1 | 0.050316266 | 0.054942682 |
| CITED2 | cluster_1 | 2.97E-22 | 5.95E-22 |
| CKB | cluster_1 | 1.43E-08 | 2.11E-08 |
| CKS2 | cluster_1 | 2.66E-32 | 6.25E-32 |
| CLCA2 | cluster_3 | 0.000141401 | 0.000176957 |
| CLDN5 | cluster_3 | 2.43E-09 | 3.68E-09 |
| CLEC10A | cluster_3 | 0.986187072 | 0.986187072 |
| CLEC14A | cluster_3 | 2.20E-44 | 5.98E-44 |
| CLEC2B | cluster_3 | 5.57E-35 | 1.35E-34 |
| CLEC3B | cluster_1 | 6.39E-164 | 4.38E-163 |
| CLEC4A | cluster_1 | 0.000295985 | 0.000364321 |
| CLEC4D | cluster_2 | 0.121869189 | 0.129943495 |
| CLEC4E | cluster_3 | 0.021730445 | 0.024346016 |
| CLEC5A | cluster_2 | 1.69E-06 | 2.33E-06 |
| CLIC3 | cluster_1 | 2.25E-05 | 2.95E-05 |
| CLIC5 | cluster_1 | 8.16E-05 | 0.000103501 |
| CLSPN | cluster_1 | 1.22E-08 | 1.80E-08 |
| CLSTN2 | cluster_2 | 7.73E-19 | 1.46E-18 |
| CLU | cluster_1 | 0 | 0 |
| CLVS2 | cluster_1 | 0.282664347 | 0.295715678 |
| CMPK2 | cluster_3 | 0.071864331 | 0.077977554 |
| CMTM8 | cluster_3 | 2.83E-79 | 1.09E-78 |
| CMYA5 | cluster_3 | 2.36E-127 | 1.28E-126 |
| CNKSR3 | cluster_3 | 1.02E-12 | 1.70E-12 |
| CNN1 | cluster_2 | 6.37E-32 | 1.49E-31 |
| CNTN1 | cluster_3 | 7.89E-42 | 2.08E-41 |
| CNTN4 | cluster_3 | 2.23E-33 | 5.32E-33 |
| COL10A1 | cluster_3 | 1.59E-07 | 2.25E-07 |
| COL11A1 | cluster_2 | 1.70E-305 | 2.06E-304 |
| COL12A1 | cluster_2 | 8.96E-90 | 3.80E-89 |
| COL14A1 | cluster_3 | 0 | 0 |
| COL15A1 | cluster_1 | 1.11E-87 | 4.61E-87 |
| COL16A1 | cluster_2 | 6.72E-201 | 5.38E-200 |
| COL18A1 | cluster_2 | 1.53E-05 | 2.02E-05 |
| COL1A1 | cluster_2 | 0 | 0 |
| COL21A1 | cluster_1 | 1.86E-08 | 2.72E-08 |
| COL22A1 | cluster_1 | 0.036552611 | 0.040322612 |
| COL23A1 | cluster_2 | 8.77E-59 | 2.81E-58 |
| COL4A1 | cluster_2 | 6.28E-115 | 3.17E-114 |
| COL4A2 | cluster_2 | 1.17E-31 | 2.71E-31 |
| COL4A4 | cluster_3 | 2.05E-171 | 1.45E-170 |
| COL5A1 | cluster_1 | 0 | 0 |
| COL5A2 | cluster_1 | 0 | 0 |
| COL5A3 | cluster_2 | 0 | 0 |
| COL6A5 | cluster_2 | 0.000113811 | 0.000143157 |
| COL6A6 | cluster_3 | 4.37E-08 | 6.28E-08 |
| COL7A1 | cluster_2 | 5.58E-95 | 2.49E-94 |
| COL8A1 | cluster_1 | 1.83E-49 | 5.30E-49 |
| COLEC11 | cluster_3 | 3.88E-08 | 5.58E-08 |
| COLEC12 | cluster_3 | 5.96448343262919e-309 | 7.50E-308 |
| COMP | cluster_1 | 0 | 0 |
| CORO1A | cluster_2 | 1.83E-15 | 3.21E-15 |
| COTL1 | cluster_2 | 7.69E-11 | 1.22E-10 |
| COX4I2 | cluster_2 | 0.017942069 | 0.020193622 |
| CP | cluster_3 | 2.13E-16 | 3.82E-16 |
| CPA3 | cluster_3 | 5.12E-08 | 7.33E-08 |
| CPB1 | cluster_3 | 3.85E-19 | 7.29E-19 |
| CPE | cluster_1 | 3.98E-56 | 1.24E-55 |
| CPM | cluster_2 | 2.90E-17 | 5.26E-17 |
| CPVL | cluster_3 | 0.000198902 | 0.000247121 |
| CPXM1 | cluster_3 | 3.92E-22 | 7.81E-22 |
| CPXM2 | cluster_3 | 1.43E-138 | 8.16E-138 |
| CRABP1 | cluster_3 | 5.47E-05 | 7.00E-05 |
| CRABP2 | cluster_1 | 0.714784227 | 0.723183571 |
| CRIP1 | cluster_1 | 1.07E-109 | 5.30E-109 |
| CRIP2 | cluster_1 | 5.11E-76 | 1.91E-75 |
| CRISPLD1 | cluster_3 | 1.23E-46 | 3.43E-46 |
| CRISPLD2 | cluster_3 | 3.46E-126 | 1.86E-125 |
| CRLF1 | cluster_1 | 4.14E-278 | 4.63E-277 |
| CRTAC1 | cluster_1 | 1.25E-193 | 9.82E-193 |
| CRYAB | cluster_1 | 1.91E-38 | 4.82E-38 |
| CSF1 | cluster_1 | 1.27E-14 | 2.19E-14 |
| CSF1R | cluster_2 | 8.08E-23 | 1.62E-22 |
| CSF3 | cluster_2 | 0.000389609 | 0.000477174 |
| CSN1S1 | cluster_1 | 1.38E-79 | 5.34E-79 |
| CSPG4 | cluster_1 | 2.24E-20 | 4.35E-20 |
| CSRP2 | cluster_2 | 0.001729412 | 0.002049585 |
| CST1 | cluster_3 | 2.28E-21 | 4.51E-21 |
| CST3 | cluster_1 | 0 | 0 |
| CST6 | cluster_1 | 2.49E-06 | 3.42E-06 |
| CST7 | cluster_2 | 1.66E-14 | 2.85E-14 |
| CSTA | cluster_3 | 0.002446934 | 0.00287227 |
| CSTB | cluster_1 | 8.11E-20 | 1.56E-19 |
| CTHRC1 | cluster_2 | 3.42E-267 | 3.56E-266 |
| CTLA4 | cluster_2 | 4.26E-05 | 5.49E-05 |
| CTSC | cluster_2 | 5.96E-33 | 1.41E-32 |
| CTSG | cluster_3 | 2.00E-10 | 3.15E-10 |
| CTSH | cluster_3 | 5.78E-170 | 4.08E-169 |
| CTSK | cluster_2 | 5.33E-09 | 7.97E-09 |
| CTSS | cluster_2 | 3.69E-13 | 6.19E-13 |
| CTSW | cluster_1 | 6.97E-10 | 1.08E-09 |
| CXCL1 | cluster_2 | 8.61E-272 | 9.33E-271 |
| CXCL10 | cluster_2 | 1.85E-08 | 2.70E-08 |
| CXCL12 | cluster_2 | 6.55E-100 | 3.00E-99 |
| CXCL13 | cluster_2 | 0 | 0 |
| CXCL14 | cluster_3 | 0 | 0 |
| CXCL16 | cluster_3 | 8.07E-55 | 2.48E-54 |
| CXCL2 | cluster_3 | 3.42610325561506e-315 | 4.40279836285702e-314 |
| CXCL3 | cluster_3 | 9.37E-40 | 2.40E-39 |
| CXCL5 | cluster_2 | 6.34E-215 | 5.35E-214 |
| CXCL6 | cluster_2 | 1.57E-103 | 7.40E-103 |
| CXCL8 | cluster_3 | 2.53E-106 | 1.23E-105 |
| CXCL9 | cluster_3 | 8.33E-06 | 1.11E-05 |
| CXCR3 | cluster_1 | 1.77E-05 | 2.34E-05 |
| CXCR4 | cluster_2 | 2.25E-21 | 4.46E-21 |
| CYBB | cluster_2 | 3.87E-11 | 6.21E-11 |
| CYGB | cluster_2 | 2.53E-08 | 3.67E-08 |
| CYP19A1 | cluster_3 | 1.97E-48 | 5.61E-48 |
| CYP1B1 | cluster_3 | 7.06E-36 | 1.74E-35 |
| CYP26A1 | cluster_1 | 6.61E-06 | 8.89E-06 |
| CYP26B1 | cluster_2 | 7.29E-12 | 1.20E-11 |
| CYP3A5 | cluster_1 | 0.97337548 | 0.975074216 |
| CYP7B1 | cluster_2 | 1.26E-12 | 2.09E-12 |
| CYTIP | cluster_2 | 0.000636227 | 0.000772625 |
| CYTL1 | cluster_2 | 3.05E-79 | 1.17E-78 |
| DACH1 | cluster_3 | 5.62E-09 | 8.39E-09 |
| DACT1 | cluster_2 | 0.015810717 | 0.017841451 |
| DBNDD2 | cluster_1 | 2.72E-12 | 4.51E-12 |
| DCBLD2 | cluster_2 | 1.26E-09 | 1.94E-09 |
| DCLK1 | cluster_3 | 2.00E-124 | 1.06E-123 |
| DDIT4 | cluster_3 | 5.03E-38 | 1.26E-37 |
| DDIT4L | cluster_1 | 0.002097815 | 0.002472578 |
| DDX3Y | cluster_2 | 5.53E-75 | 2.04E-74 |
| DDX58 | cluster_3 | 0.149041048 | 0.157840519 |
| DEFB1 | cluster_1 | 0.042513184 | 0.046688586 |
| DEPP1 | cluster_2 | 1.09E-59 | 3.52E-59 |
| DEPTOR | cluster_3 | 3.76E-44 | 1.02E-43 |
| DERL3 | cluster_2 | 6.11E-22 | 1.22E-21 |
| DES | cluster_3 | 1.21E-05 | 1.61E-05 |
| DGKI | cluster_1 | 4.00E-42 | 1.06E-41 |
| DHRS3 | cluster_3 | 7.32E-101 | 3.38E-100 |
| DIO2 | cluster_2 | 5.66E-78 | 2.14E-77 |
| DIO3 | cluster_3 | 1.93E-44 | 5.26E-44 |
| DIRAS3 | cluster_3 | 1.33E-125 | 7.13E-125 |
| DIRC3 | cluster_3 | 1.69E-16 | 3.03E-16 |
| DKK1 | cluster_3 | 1.06E-05 | 1.41E-05 |
| DKK2 | cluster_3 | 1.95E-18 | 3.64E-18 |
| DKK3 | cluster_1 | 2.93E-08 | 4.25E-08 |
| DLGAP5 | cluster_1 | 0.111832054 | 0.119537428 |
| DLK1 | cluster_3 | 0.00013753 | 0.000172363 |
| DLX4 | cluster_1 | 0.097510796 | 0.105011626 |
| DLX5 | cluster_1 | 2.89E-05 | 3.75E-05 |
| DMRT3 | cluster_3 | 2.46E-06 | 3.38E-06 |
| DNAJA4 | cluster_3 | 3.20E-47 | 9.01E-47 |
| DNAJB1 | cluster_3 | 0 | 0 |
| DNAJC18 | cluster_3 | 0.00443524 | 0.005153498 |
| DPEP1 | cluster_3 | 4.25E-11 | 6.81E-11 |
| DPP4 | cluster_3 | 3.36E-80 | 1.31E-79 |
| DPT | cluster_1 | 7.53E-43 | 2.01E-42 |
| DSC2 | cluster_2 | 2.63E-17 | 4.77E-17 |
| DSEL | cluster_2 | 3.25E-58 | 1.03E-57 |
| DSG2 | cluster_1 | 7.25E-173 | 5.23E-172 |
| DSG3 | cluster_2 | 5.18E-14 | 8.83E-14 |
| DSP | cluster_1 | 0.047128624 | 0.05159281 |
| DUSP14 | cluster_1 | 9.14E-09 | 1.35E-08 |
| DUSP2 | cluster_2 | 1.39E-11 | 2.27E-11 |
| DUSP4 | cluster_3 | 1.07E-31 | 2.47E-31 |
| DUSP5 | cluster_3 | 6.61E-79 | 2.52E-78 |
| DUSP6 | cluster_3 | 7.17E-108 | 3.50E-107 |
| DUXAP8 | cluster_3 | 5.96E-09 | 8.85E-09 |
| DYRK3 | cluster_3 | 5.15E-86 | 2.10E-85 |
| DYSF | cluster_1 | 0.001851725 | 0.002190021 |
| E2F1 | cluster_1 | 1.96E-05 | 2.57E-05 |
| EBF1 | cluster_3 | 5.28E-237 | 5.03E-236 |
| EBF2 | cluster_3 | 6.40E-87 | 2.64E-86 |
| ECEL1 | cluster_3 | 1.75E-08 | 2.56E-08 |
| ECRG4 | cluster_1 | 5.69E-21 | 1.12E-20 |
| ECSCR | cluster_2 | 0.002432113 | 0.00285682 |
| EDEM2 | cluster_1 | 1.15E-14 | 1.99E-14 |
| EDN1 | cluster_3 | 2.20E-08 | 3.21E-08 |
| EDNRA | cluster_2 | 6.70E-108 | 3.28E-107 |
| EDNRB | cluster_3 | 9.90E-81 | 3.89E-80 |
| EFEMP1 | cluster_3 | 0 | 0 |
| EFNB2 | cluster_3 | 1.90E-10 | 2.99E-10 |
| EGFL6 | cluster_2 | 0 | 0 |
| EGFR | cluster_3 | 1.21E-175 | 8.83E-175 |
| EGLN3 | cluster_1 | 1.51E-44 | 4.11E-44 |
| EGR1 | cluster_3 | 0 | 0 |
| EGR2 | cluster_3 | 1.21E-50 | 3.57E-50 |
| EGR3 | cluster_3 | 1.76E-179 | 1.32E-178 |
| EGR4 | cluster_2 | 0.111736752 | 0.119509743 |
| EIF4A3 | cluster_3 | 8.88E-50 | 2.59E-49 |
| ELL2 | cluster_3 | 1.15E-272 | 1.26E-271 |
| ELN | cluster_3 | 0 | 0 |
| EMCN | cluster_3 | 4.28E-05 | 5.50E-05 |
| EMILIN1 | cluster_2 | 1.11E-140 | 6.50E-140 |
| EMILIN2 | cluster_3 | 3.40E-49 | 9.83E-49 |
| EMP3 | cluster_1 | 1.04E-235 | 9.77E-235 |
| ENAH | cluster_2 | 2.64E-64 | 8.91E-64 |
| ENC1 | cluster_2 | 2.91E-50 | 8.58E-50 |
| ENHO | cluster_1 | 0.983998611 | 0.985142796 |
| ENPEP | cluster_2 | 1.27E-07 | 1.80E-07 |
| ENPP1 | cluster_1 | 0.000425445 | 0.000520324 |
| ENPP2 | cluster_3 | 3.01E-28 | 6.56E-28 |
| ENTPD1 | cluster_3 | 3.91E-09 | 5.88E-09 |
| ENTPD3 | cluster_3 | 0.357515608 | 0.370868601 |
| EOMES | cluster_1 | 0.021921392 | 0.024543977 |
| EPAS1 | cluster_2 | 4.40E-142 | 2.62E-141 |
| EPB41L3 | cluster_1 | 5.96E-75 | 2.20E-74 |
| EPHA7 | cluster_3 | 2.55E-05 | 3.32E-05 |
| EPSTI1 | cluster_2 | 1.87E-82 | 7.45E-82 |
| EPYC | cluster_2 | 2.85E-21 | 5.63E-21 |
| EREG | cluster_2 | 3.37E-31 | 7.71E-31 |
| ERO1B | cluster_3 | 6.15E-22 | 1.22E-21 |
| ERRFI1 | cluster_1 | 9.74E-52 | 2.91E-51 |
| ESAM | cluster_3 | 0.048079955 | 0.052600815 |
| ESM1 | cluster_3 | 1.37E-18 | 2.56E-18 |
| ETS2 | cluster_1 | 7.15E-08 | 1.02E-07 |
| EVA1A | cluster_2 | 1.38E-84 | 5.57E-84 |
| EVI2B | cluster_2 | 0.001245405 | 0.001488264 |
| EYA1 | cluster_3 | 8.63E-36 | 2.11E-35 |
| EZR | cluster_1 | 2.89E-07 | 4.07E-07 |
| F10 | cluster_3 | 7.42E-15 | 1.29E-14 |
| F13A1 | cluster_2 | 3.63E-07 | 5.10E-07 |
| F2R | cluster_3 | 3.83E-31 | 8.74E-31 |
| F3 | cluster_3 | 9.79E-19 | 1.84E-18 |
| F5 | cluster_1 | 0.026690143 | 0.02970939 |
| FABP3 | cluster_1 | 3.46E-17 | 6.26E-17 |
| FABP4 | cluster_3 | 4.20E-29 | 9.28E-29 |
| FABP5 | cluster_2 | 1.11E-46 | 3.09E-46 |
| FADS1 | cluster_3 | 4.17E-20 | 8.05E-20 |
| FADS2 | cluster_3 | 6.97E-66 | 2.38E-65 |
| FAH | cluster_1 | 1.68E-29 | 3.74E-29 |
| FAM107A | cluster_3 | 1.02E-13 | 1.73E-13 |
| FAM111B | cluster_1 | 0.135467926 | 0.14408633 |
| FAM13A | cluster_3 | 3.36E-15 | 5.87E-15 |
| FAM13C | cluster_3 | 2.75E-51 | 8.20E-51 |
| FAM180A | cluster_1 | 3.42E-65 | 1.16E-64 |
| FAM184A | cluster_3 | 2.32E-70 | 8.22E-70 |
| FAM20A | cluster_3 | 5.27E-06 | 7.11E-06 |
| FAM20C | cluster_2 | 6.40E-131 | 3.52E-130 |
| FAM241A | cluster_3 | 6.83E-18 | 1.26E-17 |
| FAM43A | cluster_3 | 7.74E-44 | 2.09E-43 |
| FAM49A | cluster_1 | 9.08E-41 | 2.36E-40 |
| FAM83D | cluster_1 | 0.379882023 | 0.393359497 |
| FBLIM1 | cluster_2 | 5.56E-85 | 2.26E-84 |
| FBLN1 | cluster_2 | 7.83E-156 | 5.07E-155 |
| FBLN2 | cluster_3 | 8.78E-44 | 2.37E-43 |
| FBLN5 | cluster_3 | 6.83E-190 | 5.27E-189 |
| FBN1 | cluster_3 | 4.98E-101 | 2.31E-100 |
| FBP1 | cluster_2 | 1.29E-05 | 1.72E-05 |
| FBXO32 | cluster_2 | 9.01E-26 | 1.89E-25 |
| FCER1A | cluster_3 | 0.636017482 | 0.64576775 |
| FCER1G | cluster_2 | 3.58E-87 | 1.48E-86 |
| FCGR1A | cluster_2 | 5.72E-06 | 7.70E-06 |
| FCGR2A | cluster_2 | 5.76E-12 | 9.49E-12 |
| FCGR2B | cluster_2 | 0.003688806 | 0.004306524 |
| FCGR3A | cluster_2 | 5.53E-24 | 1.12E-23 |
| FCGR3B | cluster_3 | 5.09E-06 | 6.87E-06 |
| FCN1 | cluster_2 | 2.27E-08 | 3.30E-08 |
| FCRL5 | cluster_2 | 0.438248896 | 0.451084637 |
| FDCSP | cluster_2 | 5.61E-80 | 2.19E-79 |
| FDPS | cluster_1 | 3.27E-07 | 4.60E-07 |
| FGF10 | cluster_3 | 3.92E-140 | 2.27E-139 |
| FGF18 | cluster_3 | 0 | 0 |
| FGF2 | cluster_1 | 3.81E-18 | 7.03E-18 |
| FGF7 | cluster_3 | 0 | 0 |
| FGFBP2 | cluster_1 | 1.18E-10 | 1.87E-10 |
| FGFR1 | cluster_2 | 2.19E-37 | 5.46E-37 |
| FGL2 | cluster_3 | 1.67E-76 | 6.29E-76 |
| FHL5 | cluster_3 | 0.01082713 | 0.012330898 |
| FIBIN | cluster_1 | 9.86E-32 | 2.29E-31 |
| FILIP1 | cluster_3 | 1.96E-127 | 1.07E-126 |
| FILIP1L | cluster_3 | 3.45E-71 | 1.23E-70 |
| FKBP11 | cluster_2 | 2.11E-99 | 9.61E-99 |
| FLRT3 | cluster_3 | 0.149830252 | 0.158578792 |
| FLT1 | cluster_3 | 2.31E-09 | 3.50E-09 |
| FMO1 | cluster_3 | 5.09E-21 | 9.98E-21 |
| FMO2 | cluster_3 | 7.56E-34 | 1.81E-33 |
| FMO3 | cluster_3 | 0.00560793 | 0.006485463 |
| FMOD | cluster_1 | 6.14E-117 | 3.15E-116 |
| FN1 | cluster_1 | 0 | 0 |
| FNDC1 | cluster_3 | 4.43E-268 | 4.68E-267 |
| FNIP2 | cluster_1 | 7.74E-25 | 1.61E-24 |
| FOLH1 | cluster_2 | 1.98E-32 | 4.67E-32 |
| FOLR2 | cluster_2 | 1.39E-05 | 1.85E-05 |
| FOSB | cluster_3 | 0 | 0 |
| FOXC2 | cluster_1 | 7.80E-22 | 1.55E-21 |
| FOXO1 | cluster_1 | 8.47E-19 | 1.60E-18 |
| FOXS1 | cluster_2 | 4.19E-16 | 7.47E-16 |
| FPR1 | cluster_3 | 5.00E-07 | 7.01E-07 |
| FPR2 | cluster_2 | 0.000337986 | 0.000414591 |
| FPR3 | cluster_2 | 3.48E-06 | 4.74E-06 |
| FRMD4A | cluster_2 | 1.37E-18 | 2.56E-18 |
| FRMD4B | cluster_1 | 8.44E-13 | 1.41E-12 |
| FRZB | cluster_3 | 1.21E-31 | 2.79E-31 |
| FST | cluster_3 | 1.00E-131 | 5.56E-131 |
| FSTL3 | cluster_3 | 1.12E-36 | 2.78E-36 |
| FTH1 | cluster_2 | 0 | 0 |
| FXYD5 | cluster_1 | 6.45E-105 | 3.07E-104 |
| FXYD6 | cluster_1 | 1.35E-13 | 2.27E-13 |
| FYB1 | cluster_2 | 9.42E-10 | 1.45E-09 |
| FYB2 | cluster_3 | 1 | 1 |
| FZD4 | cluster_3 | 7.10E-77 | 2.68E-76 |
| G0S2 | cluster_2 | 1.41E-155 | 9.12E-155 |
| GABPB1-AS1 | cluster_3 | 2.74E-09 | 4.13E-09 |
| GABRA4 | cluster_1 | 0.008030655 | 0.009219192 |
| GABRB2 | cluster_1 | 8.73E-06 | 1.17E-05 |
| GADD45A | cluster_3 | 1.22E-32 | 2.88E-32 |
| GADD45B | cluster_3 | 0 | 0 |
| GADD45G | cluster_1 | 1.04E-27 | 2.24E-27 |
| GAL | cluster_3 | 2.67E-153 | 1.70E-152 |
| GALNT1 | cluster_2 | 0.007683311 | 0.008826325 |
| GALNT15 | cluster_1 | 4.96E-31 | 1.13E-30 |
| GAP43 | cluster_1 | 3.67E-56 | 1.15E-55 |
| GAPDH | cluster_1 | 0 | 0 |
| GAS1 | cluster_3 | 1.62E-295 | 1.93E-294 |
| GAS6 | cluster_3 | 7.08E-244 | 6.97E-243 |
| GAS7 | cluster_2 | 0.004426948 | 0.005147336 |
| GASK1B | cluster_2 | 1.60E-13 | 2.69E-13 |
| GATA3 | cluster_2 | 0.001643926 | 0.001952304 |
| GATA6 | cluster_3 | 3.46E-07 | 4.86E-07 |
| GBP1 | cluster_2 | 7.12E-36 | 1.75E-35 |
| GBP2 | cluster_2 | 1.53E-28 | 3.35E-28 |
| GBP4 | cluster_2 | 3.52E-05 | 4.57E-05 |
| GBP5 | cluster_2 | 4.17E-06 | 5.65E-06 |
| GCH1 | cluster_3 | 3.22E-75 | 1.20E-74 |
| GDF10 | cluster_3 | 3.51E-10 | 5.47E-10 |
| GDF15 | cluster_2 | 6.24E-47 | 1.75E-46 |
| GDNF | cluster_2 | 5.11E-05 | 6.55E-05 |
| GEM | cluster_3 | 0 | 0 |
| GFPT2 | cluster_1 | 4.77E-32 | 1.11E-31 |
| GFRA1 | cluster_3 | 4.52E-175 | 3.28E-174 |
| GGT5 | cluster_3 | 2.64E-94 | 1.16E-93 |
| GIMAP1 | cluster_3 | 0.044018059 | 0.048248948 |
| GIMAP4 | cluster_2 | 0.017339676 | 0.019541179 |
| GIMAP5 | cluster_2 | 0.110169778 | 0.117906997 |
| GIMAP7 | cluster_3 | 5.32E-10 | 8.27E-10 |
| GINS2 | cluster_1 | 5.75E-10 | 8.92E-10 |
| GJA1 | cluster_2 | 3.33E-157 | 2.18E-156 |
| GJA4 | cluster_2 | 4.16E-05 | 5.37E-05 |
| GJA5 | cluster_2 | 1.07E-16 | 1.93E-16 |
| GJB2 | cluster_1 | 1.49E-206 | 1.23E-205 |
| GJB6 | cluster_1 | 0.804369636 | 0.81096283 |
| GLIPR1 | cluster_2 | 3.23E-06 | 4.42E-06 |
| GLIS3 | cluster_3 | 1.36E-28 | 2.98E-28 |
| GLRX | cluster_1 | 3.34E-29 | 7.39E-29 |
| GLUL | cluster_2 | 1.84E-89 | 7.76E-89 |
| GMFG | cluster_2 | 5.77E-40 | 1.48E-39 |
| GNAL | cluster_3 | 4.84E-89 | 2.02E-88 |
| GNB5 | cluster_3 | 0.015570485 | 0.017581886 |
| GNLY | cluster_2 | 0.000519678 | 0.000633771 |
| GPC3 | cluster_3 | 3.72E-118 | 1.92E-117 |
| GPC6 | cluster_2 | 4.03E-76 | 1.51E-75 |
| GPIHBP1 | cluster_3 | 0.031788844 | 0.035202822 |
| GPM6B | cluster_2 | 4.01E-46 | 1.11E-45 |
| GPR1 | cluster_2 | 8.78E-06 | 1.17E-05 |
| GPR183 | cluster_2 | 1.27E-35 | 3.09E-35 |
| GPR34 | cluster_2 | 0.420082064 | 0.43290324 |
| GPR37 | cluster_2 | 0.024662508 | 0.027559273 |
| GPR83 | cluster_3 | 0.023123661 | 0.025873258 |
| GPRC5A | cluster_3 | 0 | 0 |
| GPRC5C | cluster_1 | 9.14E-16 | 1.62E-15 |
| GPRIN3 | cluster_3 | 0.00435618 | 0.005068474 |
| GPX3 | cluster_1 | 0 | 0 |
| GREM1 | cluster_1 | 2.49E-30 | 5.61E-30 |
| GREM2 | cluster_3 | 0.000651841 | 0.000789915 |
| GRP | cluster_1 | 0.001578129 | 0.001876753 |
| GUCY1A1 | cluster_2 | 4.28E-76 | 1.61E-75 |
| GUCY1A2 | cluster_3 | 0.572798592 | 0.583644483 |
| GUCY1B1 | cluster_2 | 3.23E-21 | 6.36E-21 |
| GZMA | cluster_2 | 2.11E-26 | 4.47E-26 |
| GZMB | cluster_2 | 1.05E-22 | 2.11E-22 |
| GZMH | cluster_2 | 6.61E-11 | 1.05E-10 |
| GZMK | cluster_2 | 4.93E-21 | 9.69E-21 |
| GZMM | cluster_2 | 5.99E-08 | 8.57E-08 |
| H19 | cluster_3 | 1.06E-239 | 1.04E-238 |
| HAPLN1 | cluster_1 | 1.26E-18 | 2.36E-18 |
| HAS1 | cluster_3 | 0 | 0 |
| HAS2 | cluster_3 | 2.15E-15 | 3.77E-15 |
| HBA1 | cluster_1 | 9.35E-165 | 6.46E-164 |
| HBA2 | cluster_1 | 4.69E-184 | 3.56E-183 |
| HBB | cluster_1 | 1.04E-245 | 1.04E-244 |
| HBEGF | cluster_1 | 1.51E-10 | 2.38E-10 |
| HCAR2 | cluster_3 | 5.09E-08 | 7.29E-08 |
| HCAR3 | cluster_2 | 0.043226481 | 0.047441683 |
| HCK | cluster_2 | 5.33E-30 | 1.19E-29 |
| HCST | cluster_2 | 1.63E-35 | 3.98E-35 |
| HDC | cluster_3 | 0.059522024 | 0.06483044 |
| HELLPAR | cluster_3 | 0 | 0 |
| HEPH | cluster_2 | 6.17E-10 | 9.56E-10 |
| HES1 | cluster_3 | 1.19E-60 | 3.87E-60 |
| HES4 | cluster_2 | 2.57E-31 | 5.93E-31 |
| HEY1 | cluster_3 | 0.000108421 | 0.000136747 |
| HEY2 | cluster_2 | 2.21E-12 | 3.66E-12 |
| HEYL | cluster_2 | 0.03775076 | 0.041617675 |
| HGF | cluster_3 | 1.04E-15 | 1.83E-15 |
| HHIP | cluster_1 | 2.63E-10 | 4.12E-10 |
| HHIP-AS1 | cluster_1 | 0.008140199 | 0.009332505 |
| HIGD1A | cluster_1 | 1.16E-18 | 2.18E-18 |
| HIGD1B | cluster_2 | 9.25E-09 | 1.37E-08 |
| HILPDA | cluster_2 | 2.03E-132 | 1.13E-131 |
| HIST1H1C | cluster_2 | 5.42E-16 | 9.62E-16 |
| HIST1H2BG | cluster_2 | 6.25E-05 | 7.97E-05 |
| HLA-A | cluster_2 | 0 | 0 |
| HLA-B | cluster_2 | 0 | 0 |
| HLA-DMA | cluster_2 | 1.28E-12 | 2.12E-12 |
| HLA-DPA1 | cluster_2 | 0.010674134 | 0.012180821 |
| HLA-DPB1 | cluster_2 | 7.69E-05 | 9.76E-05 |
| HLA-DQA1 | cluster_2 | 1.55E-13 | 2.61E-13 |
| HLA-DQA2 | cluster_3 | 0.263967892 | 0.276491916 |
| HLA-DQB1 | cluster_2 | 1.80E-20 | 3.49E-20 |
| HLA-DRA | cluster_2 | 2.86E-62 | 9.45E-62 |
| HLA-DRB1 | cluster_3 | 2.13E-69 | 7.42E-69 |
| HLA-DRB5 | cluster_2 | 2.66E-35 | 6.45E-35 |
| HLA-F | cluster_2 | 2.59E-117 | 1.33E-116 |
| HMCN1 | cluster_3 | 0.004527437 | 0.005253535 |
| HMGA1 | cluster_1 | 0.108130234 | 0.115796183 |
| HMOX1 | cluster_3 | 3.22E-27 | 6.89E-27 |
| HMX1 | cluster_1 | 0.001019653 | 0.001226147 |
| HOPX | cluster_1 | 6.85E-08 | 9.76E-08 |
| HP | cluster_3 | 1.98E-204 | 1.61E-203 |
| HPD | cluster_3 | 1.59E-48 | 4.53E-48 |
| HPGD | cluster_2 | 1.21E-108 | 5.96E-108 |
| HS6ST2 | cluster_3 | 0.007125 | 0.008190421 |
| HSD11B1 | cluster_3 | 0.000170297 | 0.000211979 |
| HSD11B2 | cluster_2 | 2.06E-24 | 4.25E-24 |
| HSD17B2 | cluster_3 | 0.147940929 | 0.156771865 |
| HSD3B7 | cluster_1 | 0.000252216 | 0.00031156 |
| HSPA1A | cluster_3 | 2.78E-41 | 7.27E-41 |
| HSPA1B | cluster_3 | 5.48E-30 | 1.23E-29 |
| HSPA2 | cluster_1 | 0.01926228 | 0.021651205 |
| HSPA6 | cluster_3 | 1.10E-281 | 1.26E-280 |
| HSPB6 | cluster_3 | 5.86E-270 | 6.23E-269 |
| HSPH1 | cluster_3 | 4.49E-230 | 4.07E-229 |
| HTR2B | cluster_3 | 2.07E-29 | 4.58E-29 |
| HTRA1 | cluster_1 | 1.59E-31 | 3.66E-31 |
| HTRA3 | cluster_2 | 1.60E-53 | 4.87E-53 |
| HTRA4 | cluster_1 | 0.0033801 | 0.003948801 |
| HYMAI | cluster_3 | 1.34E-31 | 3.10E-31 |
| IBSP | cluster_2 | 0 | 0 |
| ICAM1 | cluster_3 | 2.53E-09 | 3.82E-09 |
| ICAM4 | cluster_3 | 1.54E-12 | 2.56E-12 |
| ICOS | cluster_2 | 0.000683186 | 0.000827319 |
| ID1 | cluster_1 | 1.31E-46 | 3.63E-46 |
| ID2 | cluster_3 | 1.22E-228 | 1.10E-227 |
| ID3 | cluster_3 | 3.45E-92 | 1.49E-91 |
| ID4 | cluster_3 | 9.60E-73 | 3.48E-72 |
| IDO1 | cluster_2 | 1.43E-05 | 1.89E-05 |
| IER3 | cluster_3 | 9.84E-259 | 1.00E-257 |
| IER5L | cluster_2 | 0.001222355 | 0.001461732 |
| IFI27 | cluster_1 | 8.92E-179 | 6.62E-178 |
| IFI30 | cluster_2 | 6.82E-121 | 3.58E-120 |
| IFI44L | cluster_3 | 3.10E-11 | 5.00E-11 |
| IFI6 | cluster_2 | 3.32E-08 | 4.79E-08 |
| IFIT1 | cluster_3 | 0.000121025 | 0.00015212 |
| IFIT2 | cluster_3 | 0.010737342 | 0.012236732 |
| IFIT3 | cluster_3 | 9.66E-14 | 1.64E-13 |
| IFITM1 | cluster_2 | 1.08E-24 | 2.23E-24 |
| IFNG | cluster_2 | 2.36E-13 | 3.96E-13 |
| IGF1 | cluster_3 | 0 | 0 |
| IGF2 | cluster_3 | 4.06E-13 | 6.80E-13 |
| IGFBP1 | cluster_1 | 8.27E-09 | 1.23E-08 |
| IGFBP2 | cluster_3 | 3.14E-70 | 1.11E-69 |
| IGFBP3 | cluster_3 | 1.91E-41 | 5.00E-41 |
| IGFBP4 | cluster_3 | 0 | 0 |
| IGFBP5 | cluster_3 | 0 | 0 |
| IGFBP6 | cluster_1 | 3.31E-06 | 4.51E-06 |
| IGFL2 | cluster_1 | 2.70E-06 | 3.70E-06 |
| IGHA1 | cluster_2 | 5.00E-100 | 2.29E-99 |
| IGHG1 | cluster_2 | 0 | 0 |
| IGHG2 | cluster_2 | 1.19E-139 | 6.85E-139 |
| IGHG3 | cluster_2 | 0 | 0 |
| IGHG4 | cluster_2 | 0 | 0 |
| IGHM | cluster_2 | 1 | 1 |
| IGHV3-15 | cluster_2 | 2.43E-05 | 3.17E-05 |
| IGHV3-74 | cluster_2 | 1 | 1 |
| IGKC | cluster_2 | 0 | 0 |
| IGKV1-12 | cluster_2 | 1 | 1 |
| IGLC1 | cluster_2 | 1.90E-71 | 6.78E-71 |
| IGLC2 | cluster_2 | 0 | 0 |
| IGLC3 | cluster_2 | 0 | 0 |
| IGLV2-11 | cluster_2 | 1.86E-08 | 2.72E-08 |
| IGLV4-69 | cluster_2 | 1.46E-08 | 2.15E-08 |
| IGSF10 | cluster_3 | 5.51E-12 | 9.07E-12 |
| IGSF6 | cluster_2 | 0.001107005 | 0.001328406 |
| IKZF1 | cluster_2 | 2.26E-08 | 3.29E-08 |
| IKZF3 | cluster_2 | 9.06E-08 | 1.29E-07 |
| IL10 | cluster_2 | 3.54E-06 | 4.82E-06 |
| IL11 | cluster_1 | 0.000438809 | 0.000536287 |
| IL13RA2 | cluster_3 | 0.155373375 | 0.164344565 |
| IL18 | cluster_2 | 0.204055412 | 0.215044933 |
| IL1A | cluster_2 | 0.288556283 | 0.301330455 |
| IL1B | cluster_2 | 3.44E-64 | 1.16E-63 |
| IL1R1 | cluster_2 | 3.21E-62 | 1.06E-61 |
| IL1RL1 | cluster_3 | 5.63E-47 | 1.58E-46 |
| IL1RN | cluster_2 | 7.88E-12 | 1.29E-11 |
| IL24 | cluster_2 | 6.48E-10 | 1.00E-09 |
| IL2RB | cluster_2 | 8.85E-06 | 1.18E-05 |
| IL2RG | cluster_2 | 5.87E-15 | 1.02E-14 |
| IL32 | cluster_1 | 6.44E-85 | 2.61E-84 |
| IL33 | cluster_3 | 1.05E-54 | 3.23E-54 |
| IL3RA | cluster_2 | 0.079701092 | 0.08610118 |
| IL4I1 | cluster_2 | 1.39E-43 | 3.74E-43 |
| IL6 | cluster_3 | 0 | 0 |
| IL6ST | cluster_3 | 0 | 0 |
| IL7R | cluster_2 | 0 | 0 |
| INAFM1 | cluster_2 | 2.75E-20 | 5.33E-20 |
| INHBA | cluster_2 | 0 | 0 |
| INMT | cluster_3 | 5.65E-124 | 2.98E-123 |
| INSIG1 | cluster_3 | 2.01E-33 | 4.80E-33 |
| INTS6 | cluster_3 | 7.29E-36 | 1.79E-35 |
| IQCG | cluster_1 | 4.07E-05 | 5.25E-05 |
| IQGAP2 | cluster_2 | 0.010082649 | 0.011521116 |
| IRF1 | cluster_3 | 1.22E-107 | 5.95E-107 |
| IRF4 | cluster_3 | 0.000589234 | 0.000717075 |
| IRF8 | cluster_3 | 0.000309987 | 0.000381012 |
| IRX3 | cluster_3 | 4.08E-10 | 6.35E-10 |
| ISG15 | cluster_2 | 1.37E-59 | 4.39E-59 |
| ISG20 | cluster_2 | 0.001714715 | 0.002033567 |
| ISM1 | cluster_3 | 3.27E-173 | 2.36E-172 |
| ITGA1 | cluster_2 | 1.40E-25 | 2.93E-25 |
| ITGA2 | cluster_3 | 1.15E-05 | 1.54E-05 |
| ITGA5 | cluster_1 | 2.25E-48 | 6.40E-48 |
| ITGA6 | cluster_1 | 8.92E-06 | 1.19E-05 |
| ITGA8 | cluster_3 | 1.78E-09 | 2.71E-09 |
| ITGAM | cluster_2 | 0.079611142 | 0.086057996 |
| ITGAX | cluster_1 | 0.049088802 | 0.053636369 |
| ITGB2 | cluster_2 | 4.42E-41 | 1.15E-40 |
| ITGB8 | cluster_1 | 0.034533755 | 0.038144404 |
| ITGBL1 | cluster_3 | 7.46E-221 | 6.42E-220 |
| ITIH5 | cluster_3 | 1.33397724377137e-321 | 1.78061258761185e-320 |
| ITK | cluster_2 | 0.010166229 | 0.011608917 |
| ITM2A | cluster_3 | 4.28E-287 | 5.04E-286 |
| ITPKC | cluster_3 | 1.34E-11 | 2.18E-11 |
| ITPR3 | cluster_1 | 0.003210257 | 0.003758031 |
| IVNS1ABP | cluster_1 | 0.021161846 | 0.023724413 |
| JAG1 | cluster_3 | 0.474518104 | 0.486091716 |
| JAK2 | cluster_2 | 4.07E-48 | 1.16E-47 |
| JAML | cluster_2 | 0.000210223 | 0.000260809 |
| JCHAIN | cluster_2 | 6.14E-10 | 9.52E-10 |
| JUN | cluster_3 | 0 | 0 |
| KBTBD7 | cluster_3 | 1.41E-16 | 2.54E-16 |
| KCND2 | cluster_3 | 8.00E-14 | 1.36E-13 |
| KCND3 | cluster_3 | 3.82E-30 | 8.56E-30 |
| KCNE4 | cluster_3 | 0 | 0 |
| KCNIP4 | cluster_2 | 1.06E-06 | 1.47E-06 |
| KCNJ6 | cluster_2 | 6.18E-108 | 3.03E-107 |
| KCNJ8 | cluster_3 | 3.04E-43 | 8.13E-43 |
| KCNK12 | cluster_1 | 0.011410151 | 0.012977728 |
| KCNK15 | cluster_3 | 3.57E-05 | 4.62E-05 |
| KCNK17 | cluster_3 | 0.004660593 | 0.005404405 |
| KCNK3 | cluster_2 | 9.88E-39 | 2.49E-38 |
| KCNMA1 | cluster_1 | 4.06E-67 | 1.39E-66 |
| KCNN4 | cluster_1 | 1.73E-46 | 4.80E-46 |
| KCNQ1OT1 | cluster_3 | 2.19E-264 | 2.25E-263 |
| KDM6B | cluster_3 | 8.60E-62 | 2.82E-61 |
| KERA | cluster_3 | 1.86E-160 | 1.25E-159 |
| KHDRBS3 | cluster_3 | 2.53E-73 | 9.22E-73 |
| KIAA0040 | cluster_1 | 3.31E-12 | 5.46E-12 |
| KIAA1324L | cluster_3 | 1.46E-79 | 5.62E-79 |
| KIF26B | cluster_2 | 5.65E-30 | 1.26E-29 |
| KITLG | cluster_2 | 2.15E-17 | 3.92E-17 |
| KLF4 | cluster_3 | 0 | 0 |
| KLF9 | cluster_3 | 8.48E-196 | 6.67E-195 |
| KLK1 | cluster_1 | 0.003265665 | 0.003820296 |
| KLRB1 | cluster_2 | 5.65E-09 | 8.42E-09 |
| KLRC2 | cluster_1 | 0.138176736 | 0.146605261 |
| KLRC3 | cluster_1 | 0.008530202 | 0.009773126 |
| KRT14 | cluster_1 | 0.264214934 | 0.276582441 |
| KRT16 | cluster_1 | 0.014786317 | 0.016729328 |
| KRT17 | cluster_2 | 4.64E-128 | 2.54E-127 |
| KRT18 | cluster_1 | 0.322808658 | 0.335269306 |
| KRT19 | cluster_3 | 0.363285678 | 0.376627296 |
| KRT222 | cluster_3 | 7.89E-26 | 1.66E-25 |
| KRT25 | cluster_3 | 0.000150897 | 0.00018843 |
| KRT27 | cluster_3 | 2.52E-14 | 4.32E-14 |
| KSR1 | cluster_3 | 2.23E-52 | 6.70E-52 |
| KYNU | cluster_2 | 1.50E-93 | 6.56E-93 |
| L1CAM | cluster_2 | 0.400753582 | 0.413479729 |
| LAMA2 | cluster_3 | 7.16E-135 | 4.04E-134 |
| LAMA4 | cluster_2 | 8.25E-25 | 1.71E-24 |
| LAMB1 | cluster_2 | 2.65E-35 | 6.44E-35 |
| LAMP5 | cluster_2 | 0.93135155 | 0.934608024 |
| LAPTM5 | cluster_2 | 7.95E-42 | 2.10E-41 |
| LAYN | cluster_2 | 2.49E-07 | 3.51E-07 |
| LBH | cluster_1 | 1.70E-07 | 2.40E-07 |
| LBP | cluster_2 | 1.71E-307 | 2.12E-306 |
| LCK | cluster_2 | 0.00041391 | 0.000506576 |
| LCN6 | cluster_1 | 0.917355866 | 0.921637574 |
| LCP1 | cluster_2 | 5.19E-14 | 8.85E-14 |
| LDLR | cluster_3 | 1.00E-110 | 5.04E-110 |
| LEPR | cluster_3 | 1.60E-64 | 5.42E-64 |
| LGALS2 | cluster_2 | 4.76E-06 | 6.44E-06 |
| LGI2 | cluster_2 | 5.72E-72 | 2.05E-71 |
| LGR4 | cluster_3 | 1.11E-39 | 2.83E-39 |
| LGR5 | cluster_2 | 4.22E-06 | 5.72E-06 |
| LHFPL6 | cluster_3 | 0 | 0 |
| LIF | cluster_3 | 4.74E-68 | 1.63E-67 |
| LIFR | cluster_3 | 9.68E-18 | 1.78E-17 |
| LILRB2 | cluster_2 | 0.000106776 | 0.0001348 |
| LILRB4 | cluster_2 | 9.35E-16 | 1.65E-15 |
| LILRB5 | cluster_2 | 0.066032783 | 0.071695115 |
| LIMCH1 | cluster_3 | 1.06E-11 | 1.73E-11 |
| LIMD2 | cluster_2 | 7.95E-95 | 3.53E-94 |
| LINC00324 | cluster_1 | 2.70E-06 | 3.70E-06 |
| LINC00472 | cluster_3 | 2.80E-18 | 5.19E-18 |
| LINC00603 | cluster_3 | 1.27E-20 | 2.48E-20 |
| LINC00640 | cluster_1 | 0.014834655 | 0.016772998 |
| LINC00968 | cluster_3 | 4.55E-15 | 7.92E-15 |
| LINC01088 | cluster_3 | 3.64E-21 | 7.16E-21 |
| LINC01133 | cluster_3 | 3.71E-28 | 8.05E-28 |
| LINC01140 | cluster_3 | 9.13E-49 | 2.63E-48 |
| LINC01220 | cluster_3 | 0.286312491 | 0.299168756 |
| LINC01230 | cluster_3 | 0.00059397 | 0.000722328 |
| LINC01235 | cluster_3 | 0.008782421 | 0.010055404 |
| LINC01423 | cluster_3 | 6.00E-06 | 8.08E-06 |
| LINC01436 | cluster_3 | 2.42E-22 | 4.84E-22 |
| LINC01705 | cluster_1 | 2.19E-24 | 4.50E-24 |
| LINC01833 | cluster_1 | 0.209466351 | 0.220477418 |
| LINC01929 | cluster_2 | 5.61E-26 | 1.18E-25 |
| LINC01933 | cluster_1 | 0.029003942 | 0.032201669 |
| LINC02154 | cluster_1 | 0.562303718 | 0.573290114 |
| LINC02185 | cluster_3 | 0.000168023 | 0.00020936 |
| LINC02207 | cluster_3 | 0.417150645 | 0.430139767 |
| LINC02397 | cluster_3 | 0.467219185 | 0.479184894 |
| LINC02432 | cluster_3 | 0.005827362 | 0.006725682 |
| LINC02544 | cluster_3 | 0.000451548 | 0.000551466 |
| LINC02605 | cluster_2 | 2.17E-07 | 3.06E-07 |
| LINC02802 | cluster_2 | 7.15E-06 | 9.60E-06 |
| LIPG | cluster_3 | 3.99E-15 | 6.96E-15 |
| LIX1 | cluster_2 | 0.005644374 | 0.00652323 |
| LMCD1 | cluster_3 | 3.04E-08 | 4.40E-08 |
| LMO1 | cluster_1 | 0.330359158 | 0.342904442 |
| LMO3 | cluster_3 | 3.93E-53 | 1.19E-52 |
| LMOD1 | cluster_3 | 0.453501313 | 0.4656704 |
| LNX1 | cluster_1 | 0.380726827 | 0.393997353 |
| LONRF1 | cluster_3 | 1.06E-147 | 6.40E-147 |
| LOX | cluster_1 | 0.000230086 | 0.000284633 |
| LOXL1 | cluster_2 | 1.07E-43 | 2.89E-43 |
| LOXL2 | cluster_1 | 0 | 0 |
| LPAR6 | cluster_3 | 3.74E-08 | 5.40E-08 |
| LPL | cluster_1 | 0.004066624 | 0.004734771 |
| LRRC17 | cluster_1 | 4.08E-50 | 1.20E-49 |
| LRRC32 | cluster_3 | 3.51E-14 | 6.00E-14 |
| LRRN3 | cluster_3 | 8.57E-54 | 2.62E-53 |
| LRRN4CL | cluster_3 | 7.99E-160 | 5.29E-159 |
| LSAMP | cluster_3 | 1.88E-124 | 1.00E-123 |
| LST1 | cluster_2 | 5.00E-09 | 7.48E-09 |
| LTB | cluster_2 | 5.15E-22 | 1.03E-21 |
| LTF | cluster_2 | 0.052903464 | 0.057658079 |
| LUCAT1 | cluster_3 | 2.98E-10 | 4.66E-10 |
| LXN | cluster_3 | 4.85E-224 | 4.35E-223 |
| LY6D | cluster_1 | 0.000602075 | 0.000731667 |
| LY9 | cluster_3 | 0.024340813 | 0.027217454 |
| LYPD1 | cluster_3 | 0.002253711 | 0.002650882 |
| LYPD2 | cluster_1 | 1 | 1 |
| LYPLAL1-AS1 | cluster_2 | 1.85E-08 | 2.71E-08 |
| LYVE1 | cluster_1 | 1.10E-33 | 2.63E-33 |
| LYZ | cluster_2 | 1.38E-72 | 4.99E-72 |
| MAF | cluster_2 | 4.15E-27 | 8.84E-27 |
| MAFB | cluster_2 | 0.00139586 | 0.00166344 |
| MAFF | cluster_3 | 4.80E-152 | 2.98E-151 |
| MAMDC2 | cluster_1 | 5.91E-09 | 8.79E-09 |
| MAP1B | cluster_3 | 2.47E-40 | 6.36E-40 |
| MAP3K5 | cluster_2 | 3.19E-30 | 7.15E-30 |
| MAP4K4 | cluster_2 | 2.52E-136 | 1.43E-135 |
| 1-Mar | cluster_3 | 0.159664635 | 0.168676382 |
| MARCKS | cluster_3 | 4.42E-06 | 5.98E-06 |
| MARCKSL1 | cluster_2 | 2.84E-08 | 4.13E-08 |
| MARCO | cluster_2 | 5.98E-28 | 1.29E-27 |
| MARCOL | cluster_3 | 2.02E-09 | 3.07E-09 |
| MAT2A | cluster_3 | 4.81E-35 | 1.16E-34 |
| MATN2 | cluster_1 | 4.20E-08 | 6.04E-08 |
| MCAM | cluster_2 | 1.79E-36 | 4.43E-36 |
| MCEMP1 | cluster_2 | 0.001319223 | 0.001574291 |
| MCL1 | cluster_3 | 1.27E-278 | 1.45E-277 |
| MCM10 | cluster_1 | 0.137540469 | 0.146020153 |
| MCTP1 | cluster_3 | 0.001787493 | 0.002116962 |
| MCTP2 | cluster_3 | 4.08E-38 | 1.02E-37 |
| MDK | cluster_3 | 4.55E-99 | 2.07E-98 |
| MDM2 | cluster_2 | 0.000827882 | 0.000998328 |
| MEDAG | cluster_3 | 0 | 0 |
| MEG3 | cluster_3 | 2.47E-169 | 1.73E-168 |
| MEG8 | cluster_3 | 8.84E-68 | 3.04E-67 |
| MELTF | cluster_1 | 5.34E-07 | 7.45E-07 |
| MEST | cluster_3 | 2.15E-130 | 1.18E-129 |
| MET | cluster_1 | 1.23E-06 | 1.71E-06 |
| MFAP4 | cluster_3 | 1.32E-236 | 1.25E-235 |
| MFAP5 | cluster_3 | 0 | 0 |
| MFGE8 | cluster_1 | 7.67E-12 | 1.26E-11 |
| MFSD2A | cluster_3 | 0.189292721 | 0.199609348 |
| MGAT4C | cluster_3 | 2.12E-32 | 4.99E-32 |
| MGLL | cluster_1 | 0.00161292 | 0.001916804 |
| MGP | cluster_1 | 0 | 0 |
| MGST1 | cluster_1 | 0 | 0 |
| MIA | cluster_1 | 2.57E-14 | 4.41E-14 |
| MIAT | cluster_2 | 9.78E-45 | 2.67E-44 |
| MICAL2 | cluster_1 | 1.06E-154 | 6.78E-154 |
| MIR222HG | cluster_3 | 1.44E-91 | 6.13E-91 |
| MIR503HG | cluster_3 | 0.712557555 | 0.721354562 |
| MIR99AHG | cluster_3 | 1.67E-126 | 9.01E-126 |
| MLLT11 | cluster_3 | 6.53E-46 | 1.80E-45 |
| MME | cluster_3 | 3.41E-15 | 5.95E-15 |
| MMP1 | cluster_2 | 0 | 0 |
| MMP11 | cluster_2 | 4.81E-86 | 1.97E-85 |
| MMP13 | cluster_2 | 0 | 0 |
| MMP14 | cluster_2 | 1.43E-93 | 6.29E-93 |
| MMP16 | cluster_3 | 4.99E-09 | 7.47E-09 |
| MMP2 | cluster_2 | 3.02E-58 | 9.64E-58 |
| MMP23B | cluster_3 | 1.19E-17 | 2.17E-17 |
| MMP28 | cluster_1 | 1.10E-110 | 5.53E-110 |
| MMP3 | cluster_1 | 1.88E-221 | 1.63E-220 |
| MMP7 | cluster_1 | 1.45E-05 | 1.92E-05 |
| MMP9 | cluster_1 | 0 | 0 |
| MMRN1 | cluster_3 | 0.009076311 | 0.010384988 |
| MMRN2 | cluster_3 | 0.004021602 | 0.004688692 |
| MNDA | cluster_2 | 0.0002553 | 0.000315144 |
| MOXD1 | cluster_2 | 1.23E-17 | 2.25E-17 |
| MPEG1 | cluster_2 | 2.04E-07 | 2.89E-07 |
| MRC1 | cluster_2 | 0.001167439 | 0.00139756 |
| MRPL17 | cluster_1 | 6.95E-33 | 1.65E-32 |
| MRPS6 | cluster_1 | 1.03E-68 | 3.57E-68 |
| MS4A1 | cluster_3 | 4.50E-08 | 6.47E-08 |
| MS4A2 | cluster_3 | 0.016116417 | 0.018174506 |
| MS4A4A | cluster_1 | 4.87E-11 | 7.79E-11 |
| MS4A6A | cluster_2 | 2.02E-10 | 3.17E-10 |
| MS4A7 | cluster_2 | 4.43E-11 | 7.09E-11 |
| MSC | cluster_2 | 1.05E-17 | 1.92E-17 |
| MSMO1 | cluster_1 | 0.248712466 | 0.26098895 |
| MSR1 | cluster_2 | 6.44E-05 | 8.21E-05 |
| MSTN | cluster_1 | 0.126171642 | 0.134447752 |
| MT-ND1 | cluster_3 | 9.98E-169 | 6.96E-168 |
| MT-ND6 | cluster_3 | 2.07E-46 | 5.75E-46 |
| MT1A | cluster_1 | 5.93E-57 | 1.86E-56 |
| MT1E | cluster_1 | 1.90E-216 | 1.62E-215 |
| MT1F | cluster_1 | 1.85E-235 | 1.74E-234 |
| MT1G | cluster_1 | 3.30E-254 | 3.30E-253 |
| MT1H | cluster_1 | 1.60E-28 | 3.50E-28 |
| MT1M | cluster_1 | 1.72E-11 | 2.79E-11 |
| MT1X | cluster_1 | 0.103309268 | 0.110963509 |
| MT2A | cluster_1 | 1.84E-30 | 4.14E-30 |
| MTUS1 | cluster_3 | 1.20E-141 | 7.13E-141 |
| MTUS2 | cluster_1 | 0.718695959 | 0.726714293 |
| MUSK | cluster_3 | 1.36E-10 | 2.16E-10 |
| MUSTN1 | cluster_3 | 8.26E-15 | 1.43E-14 |
| MX1 | cluster_2 | 0.000522484 | 0.000636742 |
| MX2 | cluster_3 | 2.39E-33 | 5.69E-33 |
| MXRA5 | cluster_2 | 3.41E-83 | 1.36E-82 |
| MYBPH | cluster_1 | 1.86E-05 | 2.44E-05 |
| MYC | cluster_3 | 2.68E-278 | 3.01E-277 |
| MYCT1 | cluster_3 | 0.310790754 | 0.323177342 |
| MYEOV | cluster_1 | 0.033507143 | 0.03703421 |
| MYH11 | cluster_3 | 8.26E-20 | 1.58E-19 |
| MYH9 | cluster_2 | 5.74E-216 | 4.87E-215 |
| MYL1 | cluster_3 | 3.19E-52 | 9.54E-52 |
| MYL9 | cluster_1 | 3.03356306546525e-321 | 4.01823589762686e-320 |
| MYLK | cluster_2 | 3.11E-18 | 5.76E-18 |
| MYO1E | cluster_3 | 6.26E-34 | 1.50E-33 |
| MYOC | cluster_3 | 2.47E-19 | 4.68E-19 |
| NABP1 | cluster_3 | 6.66E-40 | 1.70E-39 |
| NAMPT | cluster_3 | 1.82E-99 | 8.33E-99 |
| NCALD | cluster_3 | 9.42E-38 | 2.36E-37 |
| NCF2 | cluster_3 | 1.63E-05 | 2.15E-05 |
| NCKAP5 | cluster_3 | 0.00404317 | 0.004710649 |
| NCOA7 | cluster_3 | 1.31E-98 | 5.94E-98 |
| NDP | cluster_1 | 0.636518163 | 0.645895272 |
| NDUFA4L2 | cluster_1 | 0 | 0 |
| NEAT1 | cluster_3 | 2.18E-13 | 3.66E-13 |
| NECTIN4 | cluster_1 | 1.16E-11 | 1.90E-11 |
| NEDD9 | cluster_3 | 3.01E-12 | 4.97E-12 |
| NEFL | cluster_1 | 0.957124864 | 0.959912065 |
| NEFM | cluster_3 | 0.060060687 | 0.065375792 |
| NEGR1 | cluster_3 | 1.86E-55 | 5.75E-55 |
| NELL1 | cluster_1 | 0.048990645 | 0.053563105 |
| NES | cluster_2 | 3.68E-10 | 5.74E-10 |
| NEXN | cluster_3 | 4.77E-105 | 2.28E-104 |
| NFATC2 | cluster_3 | 3.85E-98 | 1.74E-97 |
| NFE4 | cluster_1 | 0.063813494 | 0.069329234 |
| NFIA | cluster_3 | 0 | 0 |
| NFIB | cluster_3 | 0 | 0 |
| NFIL3 | cluster_3 | 7.04E-185 | 5.39E-184 |
| NFKBIZ | cluster_3 | 2.89E-84 | 1.16E-83 |
| NGF | cluster_1 | 0.043952202 | 0.048207447 |
| NGFR | cluster_3 | 1.50E-115 | 7.66E-115 |
| NID1 | cluster_3 | 1.37E-21 | 2.71E-21 |
| NID2 | cluster_3 | 9.88E-15 | 1.71E-14 |
| NKG7 | cluster_2 | 7.13E-20 | 1.37E-19 |
| NKX2-5 | cluster_1 | 0.392691532 | 0.405647761 |
| NLGN4X | cluster_2 | 1.41E-15 | 2.48E-15 |
| NLRP3 | cluster_2 | 0.031028105 | 0.034382494 |
| NMB | cluster_1 | 2.66E-05 | 3.47E-05 |
| NOS1AP | cluster_2 | 5.33E-07 | 7.45E-07 |
| NOTCH3 | cluster_2 | 4.17E-56 | 1.30E-55 |
| NOVA1 | cluster_3 | 2.64E-307 | 3.25E-306 |
| NPAS2 | cluster_2 | 3.70E-28 | 8.05E-28 |
| NPPC | cluster_3 | 1.84E-17 | 3.35E-17 |
| NPR3 | cluster_3 | 6.94E-109 | 3.43E-108 |
| NPTX2 | cluster_2 | 4.08E-05 | 5.26E-05 |
| NPW | cluster_1 | 0.000121821 | 0.000153009 |
| NPY1R | cluster_3 | 5.39E-20 | 1.04E-19 |
| NQO1 | cluster_1 | 1.17E-09 | 1.80E-09 |
| NR2F1 | cluster_2 | 0.702869707 | 0.711965668 |
| NR2F2 | cluster_3 | 7.08E-29 | 1.56E-28 |
| NR4A1 | cluster_3 | 0 | 0 |
| NR4A2 | cluster_3 | 0 | 0 |
| NR4A3 | cluster_3 | 1.28E-239 | 1.25E-238 |
| NRARP | cluster_3 | 0.019806194 | 0.02223355 |
| NRCAM | cluster_2 | 5.15E-27 | 1.10E-26 |
| NREP | cluster_1 | 1.15E-168 | 7.96E-168 |
| NRG1 | cluster_3 | 0.028289249 | 0.031448733 |
| NRIP1 | cluster_3 | 6.92E-51 | 2.05E-50 |
| NRK | cluster_3 | 5.14E-45 | 1.41E-44 |
| NRN1 | cluster_1 | 0.025814813 | 0.028772239 |
| NRXN1 | cluster_3 | 0.002287579 | 0.002688881 |
| NSG1 | cluster_2 | 4.91E-23 | 9.89E-23 |
| NT5DC2 | cluster_1 | 2.39E-204 | 1.93E-203 |
| NTM | cluster_3 | 4.13E-12 | 6.81E-12 |
| NTN1 | cluster_2 | 1.59E-57 | 5.01E-57 |
| NTN4 | cluster_1 | 1.00E-29 | 2.22E-29 |
| NTNG1 | cluster_3 | 3.51E-06 | 4.77E-06 |
| NTRK2 | cluster_3 | 9.13E-172 | 6.53E-171 |
| NTRK3 | cluster_2 | 7.86E-50 | 2.30E-49 |
| NUAK1 | cluster_2 | 0.001666228 | 0.001977425 |
| NXN | cluster_2 | 5.18E-92 | 2.22E-91 |
| NXPH4 | cluster_1 | 3.28E-09 | 4.95E-09 |
| OAF | cluster_3 | 1.75E-207 | 1.45E-206 |
| OAS1 | cluster_2 | 0.014558803 | 0.016482748 |
| OASL | cluster_3 | 0.754656715 | 0.762628441 |
| OCSTAMP | cluster_1 | 0.105782556 | 0.113423139 |
| ODAM | cluster_1 | 0.984815381 | 0.985387615 |
| OGN | cluster_3 | 0 | 0 |
| OLFM2 | cluster_2 | 4.19E-46 | 1.16E-45 |
| OLFML2A | cluster_1 | 5.43E-06 | 7.32E-06 |
| OLFML2B | cluster_2 | 3.89E-78 | 1.48E-77 |
| OLFML3 | cluster_2 | 4.94E-16 | 8.79E-16 |
| OLR1 | cluster_2 | 4.10E-05 | 5.29E-05 |
| OMD | cluster_1 | 3.87E-53 | 1.17E-52 |
| OMG | cluster_3 | 5.71E-09 | 8.50E-09 |
| OR51E1 | cluster_2 | 0.304766985 | 0.317104983 |
| OSBPL1A | cluster_3 | 3.07E-106 | 1.48E-105 |
| OSM | cluster_2 | 0.008054796 | 0.009240746 |
| OSR1 | cluster_3 | 5.31E-70 | 1.87E-69 |
| OSR2 | cluster_3 | 1.45E-25 | 3.03E-25 |
| P2RY13 | cluster_2 | 0.399955544 | 0.412903745 |
| P2RY14 | cluster_3 | 0.019350185 | 0.021735824 |
| PAG1 | cluster_2 | 1.03E-06 | 1.44E-06 |
| PALM2-AKAP2 | cluster_2 | 3.71E-10 | 5.77E-10 |
| PALMD | cluster_1 | 4.34E-127 | 2.35E-126 |
| PAPLN | cluster_2 | 2.20E-24 | 4.52E-24 |
| PAPPA | cluster_3 | 2.57E-18 | 4.78E-18 |
| PAPPA2 | cluster_3 | 8.94E-103 | 4.18E-102 |
| PARD6G-AS1 | cluster_3 | 0.000329733 | 0.000404993 |
| PARP14 | cluster_2 | 6.09E-16 | 1.08E-15 |
| PARP16 | cluster_1 | 4.03E-05 | 5.21E-05 |
| PART1 | cluster_1 | 1.81E-06 | 2.50E-06 |
| PAWR | cluster_2 | 0.290757895 | 0.303445513 |
| PBK | cluster_1 | 0.000104111 | 0.000131725 |
| PCDH17 | cluster_3 | 0.002242432 | 0.002639418 |
| PCDH18 | cluster_3 | 3.08E-94 | 1.35E-93 |
| PCDH7 | cluster_2 | 3.91E-37 | 9.74E-37 |
| PCDH8 | cluster_3 | 1.01E-06 | 1.40E-06 |
| PCDH9 | cluster_3 | 1.21E-156 | 7.88E-156 |
| PCDHB4 | cluster_3 | 1.15E-19 | 2.20E-19 |
| PCDHGA12 | cluster_3 | 0.00022051 | 0.000273179 |
| PCDHGA5 | cluster_3 | 1.17E-17 | 2.14E-17 |
| PCED1B-AS1 | cluster_2 | 7.54E-10 | 1.16E-09 |
| PCK1 | cluster_1 | 2.49E-17 | 4.54E-17 |
| PCLAF | cluster_1 | 1.29E-42 | 3.44E-42 |
| PCOLCE2 | cluster_1 | 0.000139112 | 0.000174218 |
| PCSK1 | cluster_3 | 1.60E-232 | 1.47E-231 |
| PCSK5 | cluster_3 | 8.58E-75 | 3.14E-74 |
| PCSK6 | cluster_1 | 2.07E-24 | 4.27E-24 |
| PDE10A | cluster_3 | 6.35E-107 | 3.08E-106 |
| PDE3B | cluster_3 | 6.92E-19 | 1.31E-18 |
| PDE4B | cluster_3 | 3.71E-271 | 3.99E-270 |
| PDE4D | cluster_3 | 1.17E-306 | 1.43E-305 |
| PDE5A | cluster_3 | 4.86E-153 | 3.07E-152 |
| PDGFA | cluster_1 | 0.000268914 | 0.000331474 |
| PDGFD | cluster_3 | 1.70639503442487e-317 | 2.24306277514946e-316 |
| PDGFRA | cluster_3 | 1.84E-30 | 4.14E-30 |
| PDK4 | cluster_3 | 0 | 0 |
| PDLIM3 | cluster_1 | 0.002002906 | 0.002363951 |
| PDPN | cluster_1 | 2.22E-222 | 1.94E-221 |
| PDZRN4 | cluster_3 | 8.76E-153 | 5.49E-152 |
| PECAM1 | cluster_3 | 7.10E-12 | 1.17E-11 |
| PEG10 | cluster_3 | 0 | 0 |
| PENK | cluster_1 | 1.96E-163 | 1.34E-162 |
| PFKFB3 | cluster_3 | 7.49E-16 | 1.33E-15 |
| PFKP | cluster_1 | 2.26E-10 | 3.55E-10 |
| PGAM2 | cluster_1 | 0.303816902 | 0.31630756 |
| PGF | cluster_2 | 3.06E-61 | 9.99E-61 |
| PGK1 | cluster_1 | 7.95E-92 | 3.40E-91 |
| PHACTR1 | cluster_2 | 2.69E-05 | 3.51E-05 |
| PHC2 | cluster_2 | 3.05E-22 | 6.09E-22 |
| PHGDH | cluster_3 | 0.000684137 | 0.000827887 |
| PHLDA1 | cluster_3 | 8.44E-141 | 4.95E-140 |
| PHLDA2 | cluster_1 | 1.71E-69 | 6.00E-69 |
| PHLDB2 | cluster_3 | 3.11E-62 | 1.03E-61 |
| PHYHIP | cluster_3 | 1.75E-18 | 3.28E-18 |
| PI15 | cluster_2 | 7.84E-53 | 2.36E-52 |
| PI16 | cluster_3 | 1.15E-209 | 9.61E-209 |
| PI3 | cluster_2 | 3.55E-09 | 5.35E-09 |
| PIEZO2 | cluster_3 | 3.50E-83 | 1.40E-82 |
| PIK3R1 | cluster_2 | 9.21E-87 | 3.78E-86 |
| PIK3R3 | cluster_3 | 1.93E-39 | 4.91E-39 |
| PIM1 | cluster_3 | 0.005673812 | 0.006552853 |
| PIM2 | cluster_2 | 2.25E-05 | 2.95E-05 |
| PKDCC | cluster_3 | 6.69E-163 | 4.53E-162 |
| PLA2G2A | cluster_3 | 0 | 0 |
| PLAAT4 | cluster_3 | 1.83E-25 | 3.82E-25 |
| PLAC8 | cluster_2 | 0.040414068 | 0.044496819 |
| PLAT | cluster_2 | 3.82E-08 | 5.50E-08 |
| PLAU | cluster_2 | 9.66E-79 | 3.68E-78 |
| PLCXD3 | cluster_3 | 3.47E-06 | 4.74E-06 |
| PLEK | cluster_2 | 6.97E-21 | 1.36E-20 |
| PLEKHA1 | cluster_1 | 1.87E-07 | 2.64E-07 |
| PLIN2 | cluster_3 | 1.70E-05 | 2.24E-05 |
| PLK2 | cluster_3 | 2.56E-09 | 3.87E-09 |
| PLN | cluster_3 | 0.000338029 | 0.000414591 |
| PLP2 | cluster_1 | 8.28E-125 | 4.41E-124 |
| PLPP1 | cluster_3 | 0 | 0 |
| PLPP3 | cluster_3 | 0 | 0 |
| PLPPR4 | cluster_3 | 0.006151474 | 0.007095002 |
| PLTP | cluster_1 | 3.25E-06 | 4.44E-06 |
| PLVAP | cluster_3 | 0.0044452 | 0.005161587 |
| PLXDC1 | cluster_1 | 1.51E-17 | 2.76E-17 |
| PLXNA4 | cluster_3 | 5.58E-47 | 1.57E-46 |
| PMAIP1 | cluster_1 | 9.55E-10 | 1.47E-09 |
| PNRC1 | cluster_3 | 0 | 0 |
| PODN | cluster_3 | 0 | 0 |
| PODXL | cluster_3 | 0.026484124 | 0.029499135 |
| PON1 | cluster_3 | 0.000238231 | 0.000294497 |
| PON3 | cluster_3 | 6.63E-28 | 1.43E-27 |
| POPDC3 | cluster_1 | 1.67E-89 | 7.04E-89 |
| POSTN | cluster_1 | 0 | 0 |
| POU2AF1 | cluster_2 | 0.452717391 | 0.465142808 |
| POU2F2 | cluster_2 | 6.21E-28 | 1.34E-27 |
| POU3F1 | cluster_3 | 0.001090864 | 0.00130995 |
| PPDPFL | cluster_3 | 1.60E-39 | 4.06E-39 |
| PPIF | cluster_3 | 0.035608203 | 0.039305978 |
| PPL | cluster_3 | 5.56E-30 | 1.24E-29 |
| PPP1R12B | cluster_3 | 1.70E-27 | 3.65E-27 |
| PPP1R14A | cluster_3 | 0.000651569 | 0.000789915 |
| PPP1R14C | cluster_1 | 1.10E-08 | 1.62E-08 |
| PPP1R15B | cluster_3 | 2.09E-80 | 8.21E-80 |
| PPP1R1A | cluster_1 | 0.017608568 | 0.019831232 |
| PRDM1 | cluster_2 | 3.91E-153 | 2.48E-152 |
| PRDX1 | cluster_1 | 1.87E-104 | 8.88E-104 |
| PRELP | cluster_1 | 0 | 0 |
| PREX2 | cluster_3 | 6.06E-29 | 1.33E-28 |
| PRF1 | cluster_1 | 0.44938163 | 0.461991144 |
| PRG4 | cluster_1 | 6.63E-233 | 6.10E-232 |
| PRICKLE1 | cluster_3 | 3.71E-116 | 1.90E-115 |
| PRKAR2B | cluster_3 | 3.64E-06 | 4.93E-06 |
| PRKY | cluster_2 | 4.14E-24 | 8.44E-24 |
| PRL | cluster_1 | 0.073042516 | 0.079183202 |
| PRLR | cluster_2 | 2.79E-62 | 9.25E-62 |
| PRND | cluster_3 | 0.284840685 | 0.297811572 |
| PROCR | cluster_1 | 6.13E-08 | 8.76E-08 |
| PROK1 | cluster_3 | 1.55E-48 | 4.45E-48 |
| PROK2 | cluster_2 | 0.006771974 | 0.007789805 |
| PRSS23 | cluster_1 | 6.75E-73 | 2.45E-72 |
| PRSS35 | cluster_3 | 0.60618209 | 0.616201628 |
| PSAT1 | cluster_1 | 2.84E-11 | 4.58E-11 |
| PSMB9 | cluster_2 | 2.14E-152 | 1.33E-151 |
| PSTPIP1 | cluster_2 | 1.40E-06 | 1.94E-06 |
| PTAFR | cluster_2 | 0.00218077 | 0.002568595 |
| PTGDS | cluster_3 | 0 | 0 |
| PTGER3 | cluster_3 | 2.25E-29 | 4.97E-29 |
| PTGER4 | cluster_3 | 3.33E-74 | 1.22E-73 |
| PTGES | cluster_1 | 2.21E-11 | 3.58E-11 |
| PTGFR | cluster_3 | 8.05E-144 | 4.81E-143 |
| PTGIS | cluster_3 | 4.85E-263 | 4.97E-262 |
| PTGS2 | cluster_3 | 6.40E-57 | 2.00E-56 |
| PTHLH | cluster_1 | 4.09E-23 | 8.26E-23 |
| PTN | cluster_3 | 0 | 0 |
| PTPRB | cluster_3 | 0.000794189 | 0.000958369 |
| PTPRC | cluster_2 | 4.05E-15 | 7.06E-15 |
| PTPRN | cluster_2 | 4.21E-100 | 1.94E-99 |
| PTPRZ1 | cluster_1 | 0.096013491 | 0.103463849 |
| PTTG1 | cluster_1 | 4.12E-42 | 1.09E-41 |
| PTX3 | cluster_3 | 2.53E-18 | 4.70E-18 |
| QPRT | cluster_3 | 1.63E-08 | 2.39E-08 |
| RAB11FIP1 | cluster_2 | 0.00113604 | 0.001362298 |
| RAB27B | cluster_2 | 6.49E-24 | 1.32E-23 |
| RAB38 | cluster_1 | 0.173446818 | 0.183124109 |
| RAC2 | cluster_1 | 1.10E-28 | 2.41E-28 |
| RAI14 | cluster_2 | 2.16E-102 | 1.00E-101 |
| RALGPS2 | cluster_3 | 0.00116788 | 0.00139756 |
| RAMP1 | cluster_3 | 7.73E-32 | 1.80E-31 |
| RAMP2 | cluster_1 | 5.09E-35 | 1.23E-34 |
| RAMP3 | cluster_2 | 0.000141646 | 0.000177135 |
| RANBP3L | cluster_1 | 1.70E-36 | 4.19E-36 |
| RAPGEF4 | cluster_1 | 6.28E-05 | 8.01E-05 |
| RAPGEF5 | cluster_3 | 5.45E-08 | 7.80E-08 |
| RARRES1 | cluster_3 | 0 | 0 |
| RARRES2 | cluster_2 | 6.99E-81 | 2.76E-80 |
| RASD1 | cluster_3 | 0 | 0 |
| RASGRP3 | cluster_2 | 0.156994359 | 0.165957204 |
| RBBP6 | cluster_3 | 1.75E-53 | 5.33E-53 |
| RBMS3 | cluster_3 | 1.04E-171 | 7.42E-171 |
| RBP4 | cluster_1 | 1.37E-13 | 2.30E-13 |
| RBP5 | cluster_3 | 9.80E-20 | 1.88E-19 |
| RBP7 | cluster_3 | 1.61E-06 | 2.23E-06 |
| RCAN1 | cluster_1 | 2.58E-31 | 5.93E-31 |
| RCAN2 | cluster_2 | 8.48E-42 | 2.23E-41 |
| RCN1 | cluster_2 | 4.20E-72 | 1.51E-71 |
| RDH10 | cluster_3 | 4.83E-58 | 1.53E-57 |
| RDH5 | cluster_2 | 5.05E-16 | 8.97E-16 |
| REL | cluster_3 | 6.10E-80 | 2.37E-79 |
| RERG | cluster_1 | 3.95E-07 | 5.53E-07 |
| RERGL | cluster_3 | 2.40E-10 | 3.76E-10 |
| RETN | cluster_2 | 0.905932802 | 0.911224465 |
| REV3L | cluster_3 | 1.83E-197 | 1.45E-196 |
| RFLNA | cluster_2 | 1.70E-26 | 3.60E-26 |
| RFPL4B | cluster_3 | 0.001249147 | 0.0014917 |
| RGCC | cluster_1 | 0 | 0 |
| RGS1 | cluster_2 | 1.12E-35 | 2.73E-35 |
| RGS10 | cluster_1 | 7.41E-41 | 1.93E-40 |
| RGS16 | cluster_1 | 5.73E-09 | 8.53E-09 |
| RGS2 | cluster_3 | 1.31E-91 | 5.60E-91 |
| RGS3 | cluster_1 | 1.70E-239 | 1.65E-238 |
| RGS4 | cluster_3 | 0.000899756 | 0.001082725 |
| RGS5 | cluster_3 | 6.27E-20 | 1.21E-19 |
| RHEX | cluster_3 | 0.000121991 | 0.000153111 |
| RHOBTB3 | cluster_3 | 3.42E-21 | 6.74E-21 |
| RHOH | cluster_2 | 1.42E-09 | 2.17E-09 |
| RHOU | cluster_3 | 1.26E-48 | 3.62E-48 |
| RIPOR3 | cluster_3 | 7.12E-15 | 1.24E-14 |
| RMDN2-AS1 | cluster_3 | 0.014863495 | 0.016794579 |
| RNASE1 | cluster_2 | 3.60E-31 | 8.23E-31 |
| RND3 | cluster_3 | 1.71E-115 | 8.69E-115 |
| RNF152 | cluster_3 | 2.39E-08 | 3.47E-08 |
| RNF175 | cluster_3 | 1.76E-11 | 2.86E-11 |
| ROR1-AS1 | cluster_3 | 0.000112385 | 0.000141466 |
| RPL13A | cluster_3 | 1.66E-256 | 1.68E-255 |
| RPRM | cluster_3 | 1.09E-08 | 1.61E-08 |
| RPS2 | cluster_2 | 7.97E-88 | 3.31E-87 |
| RPS4Y1 | cluster_2 | 0 | 0 |
| RPTN | cluster_1 | 0.585785733 | 0.59652456 |
| RRAD | cluster_1 | 9.70E-60 | 3.13E-59 |
| RSAD2 | cluster_3 | 3.61E-24 | 7.39E-24 |
| RSPO2 | cluster_1 | 1.67E-05 | 2.20E-05 |
| RSPO3 | cluster_2 | 3.65E-40 | 9.39E-40 |
| RTN1 | cluster_1 | 0.513790869 | 0.524761492 |
| RUNX1T1 | cluster_3 | 1.49E-69 | 5.22E-69 |
| RUNX2 | cluster_2 | 1.82E-191 | 1.42E-190 |
| S100A10 | cluster_1 | 7.63E-82 | 3.02E-81 |
| S100A12 | cluster_3 | 6.68E-10 | 1.03E-09 |
| S100A3 | cluster_1 | 5.36E-51 | 1.59E-50 |
| S100A4 | cluster_1 | 1.52E-284 | 1.75E-283 |
| S100A8 | cluster_2 | 4.35E-71 | 1.54E-70 |
| S100A9 | cluster_2 | 6.04E-88 | 2.51E-87 |
| S100B | cluster_1 | 3.13E-15 | 5.48E-15 |
| S1PR3 | cluster_2 | 5.08E-96 | 2.28E-95 |
| SAA1 | cluster_3 | 2.10E-24 | 4.31E-24 |
| SAA2 | cluster_3 | 0.002537436 | 0.002976474 |
| SAMHD1 | cluster_3 | 9.66E-41 | 2.51E-40 |
| SAMSN1 | cluster_3 | 0.004774665 | 0.005532956 |
| SBSN | cluster_1 | 1.22E-118 | 6.34E-118 |
| SCARA3 | cluster_1 | 2.77E-67 | 9.48E-67 |
| SCARA5 | cluster_2 | 3.11E-28 | 6.77E-28 |
| SCG2 | cluster_3 | 0 | 0 |
| SCG5 | cluster_2 | 5.45E-09 | 8.14E-09 |
| SCIMP | cluster_2 | 0.112817107 | 0.120515544 |
| SCRG1 | cluster_3 | 1.39E-156 | 9.06E-156 |
| SCUBE2 | cluster_3 | 7.24E-53 | 2.18E-52 |
| SCX | cluster_1 | 0.040477139 | 0.044537785 |
| SDC1 | cluster_2 | 3.07E-18 | 5.70E-18 |
| SDC2 | cluster_1 | 7.22E-46 | 1.99E-45 |
| SDC4 | cluster_1 | 3.51E-10 | 5.48E-10 |
| SDF2L1 | cluster_1 | 3.16E-33 | 7.50E-33 |
| SDK1 | cluster_2 | 8.29E-60 | 2.68E-59 |
| SDS | cluster_2 | 3.38E-05 | 4.38E-05 |
| SEC11C | cluster_1 | 0.003709286 | 0.0043275 |
| SELE | cluster_3 | 1.59E-05 | 2.10E-05 |
| SELENOP | cluster_3 | 0 | 0 |
| SELP | cluster_1 | 0.803079124 | 0.810136058 |
| SEMA3A | cluster_1 | 0.005267451 | 0.0060958 |
| SEMA3C | cluster_1 | 0 | 0 |
| SEMA3D | cluster_3 | 3.16E-47 | 8.92E-47 |
| SEMA3E | cluster_1 | 0.013660884 | 0.015476344 |
| SEMA4A | cluster_3 | 2.60E-69 | 9.05E-69 |
| SEMA5A | cluster_2 | 5.39E-26 | 1.14E-25 |
| SEMA6A | cluster_3 | 9.49E-17 | 1.71E-16 |
| SEPTIN4 | cluster_2 | 3.56E-21 | 7.00E-21 |
| SERINC2 | cluster_1 | 9.30E-12 | 1.52E-11 |
| SERPINA1 | cluster_1 | 1.28E-13 | 2.16E-13 |
| SERPINA11 | cluster_1 | 0.896800633 | 0.902566155 |
| SERPINA3 | cluster_3 | 5.12E-239 | 4.90E-238 |
| SERPINA9 | cluster_1 | 0.607050914 | 0.616720752 |
| SERPINB2 | cluster_2 | 0.051790818 | 0.056516976 |
| SERPINB4 | cluster_2 | 0.005178225 | 0.005996573 |
| SERPINE1 | cluster_1 | 4.88E-155 | 3.12E-154 |
| SERPINE2 | cluster_3 | 4.60E-51 | 1.37E-50 |
| SERTAD4-AS1 | cluster_1 | 5.96E-39 | 1.51E-38 |
| SERTM1 | cluster_3 | 1.34E-09 | 2.04E-09 |
| SESN3 | cluster_3 | 1.30E-146 | 7.82E-146 |
| SEZ6L2 | cluster_2 | 8.12E-298 | 9.71E-297 |
| SFRP1 | cluster_3 | 0 | 0 |
| SFRP2 | cluster_3 | 0 | 0 |
| SFRP4 | cluster_3 | 0 | 0 |
| SFTA1P | cluster_3 | 0.483008925 | 0.494495463 |
| SGCA | cluster_1 | 6.26E-28 | 1.35E-27 |
| SGK1 | cluster_1 | 5.00E-268 | 5.25E-267 |
| SGPP2 | cluster_2 | 7.80E-30 | 1.73E-29 |
| SH3BGRL3 | cluster_1 | 0 | 0 |
| SH3PXD2B | cluster_3 | 1.41E-07 | 2.00E-07 |
| SIGLEC12 | cluster_2 | 1 | 1 |
| SIGLEC15 | cluster_1 | 5.84E-05 | 7.46E-05 |
| SIX3 | cluster_1 | 8.44E-11 | 1.34E-10 |
| SIX3-AS1 | cluster_1 | 0.003100651 | 0.003632192 |
| SLA | cluster_2 | 4.96E-11 | 7.93E-11 |
| SLAMF1 | cluster_2 | 5.17E-24 | 1.05E-23 |
| SLAMF7 | cluster_2 | 0.000726839 | 0.000878327 |
| SLAMF9 | cluster_2 | 0.217300347 | 0.228583505 |
| SLC11A1 | cluster_2 | 1.53E-08 | 2.26E-08 |
| SLC16A10 | cluster_2 | 1.01E-20 | 1.97E-20 |
| SLC16A3 | cluster_1 | 1.32E-233 | 1.22E-232 |
| SLC16A4 | cluster_3 | 1.12E-159 | 7.41E-159 |
| SLC19A2 | cluster_3 | 1.46E-222 | 1.28E-221 |
| SLC1A3 | cluster_2 | 1.95E-18 | 3.63E-18 |
| SLC1A7 | cluster_3 | 3.88E-27 | 8.30E-27 |
| SLC20A1 | cluster_1 | 7.57E-05 | 9.62E-05 |
| SLC29A1 | cluster_1 | 7.74E-17 | 1.40E-16 |
| SLC2A12 | cluster_1 | 3.86E-09 | 5.82E-09 |
| SLC2A3 | cluster_3 | 7.23E-153 | 4.55E-152 |
| SLC2A5 | cluster_1 | 8.57E-76 | 3.19E-75 |
| SLC2A6 | cluster_2 | 2.22E-55 | 6.87E-55 |
| SLC38A11 | cluster_3 | 0.075544697 | 0.081764907 |
| SLC38A5 | cluster_1 | 4.33E-80 | 1.69E-79 |
| SLC39A14 | cluster_1 | 3.54E-150 | 2.17E-149 |
| SLC39A8 | cluster_2 | 5.70E-14 | 9.69E-14 |
| SLC40A1 | cluster_1 | 6.02E-27 | 1.28E-26 |
| SLC43A2 | cluster_2 | 1.13E-09 | 1.74E-09 |
| SLC43A3 | cluster_3 | 9.67E-17 | 1.74E-16 |
| SLC51A | cluster_2 | 3.37E-31 | 7.71E-31 |
| SLC5A3 | cluster_2 | 1.45E-27 | 3.12E-27 |
| SLC5A5 | cluster_2 | 1.79E-42 | 4.76E-42 |
| SLC7A2 | cluster_1 | 1.95E-37 | 4.86E-37 |
| SLC9A3R1 | cluster_1 | 0.033417563 | 0.036958923 |
| SLC9B2 | cluster_1 | 1.65E-05 | 2.19E-05 |
| SLCO2A1 | cluster_1 | 4.33E-18 | 7.98E-18 |
| SLCO2B1 | cluster_3 | 0.061101311 | 0.066424532 |
| SLCO4A1 | cluster_1 | 0.001860338 | 0.002198697 |
| SLFN5 | cluster_3 | 7.23E-16 | 1.28E-15 |
| SLIT3 | cluster_3 | 1.40E-89 | 5.92E-89 |
| SLITRK6 | cluster_3 | 1.62E-08 | 2.38E-08 |
| SLN | cluster_1 | 1.87E-17 | 3.41E-17 |
| SLPI | cluster_3 | 3.37721606271915e-315 | 4.37260606015217e-314 |
| SLURP1 | cluster_1 | 0.46858992 | 0.480304668 |
| SLURP2 | cluster_1 | 0.686856453 | 0.696154686 |
| SMIM14 | cluster_1 | 2.16E-49 | 6.27E-49 |
| SMOC1 | cluster_1 | 0.926687686 | 0.930470085 |
| SMOC2 | cluster_3 | 4.55184333601993e-311 | 5.76343692987229e-310 |
| SNAI2 | cluster_3 | 3.08E-19 | 5.84E-19 |
| SNCA | cluster_1 | 0.373337052 | 0.386814924 |
| SNCG | cluster_1 | 5.66E-11 | 9.02E-11 |
| SNTG1 | cluster_3 | 3.90E-06 | 5.29E-06 |
| SNX10 | cluster_3 | 2.54E-17 | 4.62E-17 |
| SOCS3 | cluster_3 | 2.30E-275 | 2.54E-274 |
| SOD2 | cluster_3 | 9.30E-76 | 3.46E-75 |
| SOD3 | cluster_1 | 0.106180568 | 0.113779053 |
| SOX4 | cluster_3 | 0.24575657 | 0.258044398 |
| SOX5 | cluster_1 | 4.92E-28 | 1.07E-27 |
| SOX9 | cluster_1 | 4.01E-68 | 1.39E-67 |
| SP6 | cluster_2 | 2.14E-18 | 3.98E-18 |
| SPARCL1 | cluster_1 | 0 | 0 |
| SPHK1 | cluster_2 | 1.52E-110 | 7.57E-110 |
| SPI1 | cluster_2 | 5.32E-23 | 1.07E-22 |
| SPIB | cluster_2 | 0.073067426 | 0.079183202 |
| SPINK1 | cluster_2 | 1 | 1 |
| SPINK13 | cluster_3 | 1.40E-12 | 2.33E-12 |
| SPINK6 | cluster_3 | 0.130790162 | 0.139196947 |
| SPINT2 | cluster_3 | 0.423541239 | 0.436206946 |
| SPN | cluster_2 | 0.025797455 | 0.028771514 |
| SPOCD1 | cluster_2 | 7.87E-75 | 2.89E-74 |
| SPOCK1 | cluster_1 | 4.13E-92 | 1.77E-91 |
| SPOCK2 | cluster_2 | 2.02E-06 | 2.79E-06 |
| SPON1 | cluster_3 | 0 | 0 |
| SPON2 | cluster_1 | 9.94E-136 | 5.63E-135 |
| SPP1 | cluster_1 | 0 | 0 |
| SPRY1 | cluster_3 | 2.78E-53 | 8.45E-53 |
| SPRY2 | cluster_3 | 6.95E-93 | 3.02E-92 |
| SRGN | cluster_3 | 4.67E-141 | 2.75E-140 |
| SRPX | cluster_3 | 1.72E-119 | 8.97E-119 |
| SSTR2 | cluster_2 | 4.32E-09 | 6.49E-09 |
| ST3GAL5 | cluster_3 | 8.55E-71 | 3.03E-70 |
| ST8SIA2 | cluster_2 | 0.968468729 | 0.970723604 |
| ST8SIA4 | cluster_3 | 7.40E-17 | 1.34E-16 |
| STAB1 | cluster_2 | 5.06E-07 | 7.07E-07 |
| STAR | cluster_2 | 2.79E-06 | 3.81E-06 |
| STAT1 | cluster_3 | 0.000838885 | 0.001010889 |
| STC1 | cluster_3 | 2.23E-08 | 3.25E-08 |
| STC2 | cluster_2 | 9.64E-14 | 1.63E-13 |
| STEAP2 | cluster_3 | 2.67E-115 | 1.35E-114 |
| STEAP4 | cluster_3 | 0 | 0 |
| STMN1 | cluster_2 | 3.46E-34 | 8.33E-34 |
| STMN2 | cluster_3 | 3.26E-106 | 1.57E-105 |
| STOM | cluster_3 | 5.13E-205 | 4.21E-204 |
| STON2 | cluster_2 | 1.10E-07 | 1.56E-07 |
| STRA6 | cluster_1 | 6.35E-61 | 2.07E-60 |
| STXBP2 | cluster_2 | 3.21E-16 | 5.72E-16 |
| SUCNR1 | cluster_3 | 0.098429934 | 0.105935217 |
| SUGCT | cluster_1 | 1.02E-12 | 1.70E-12 |
| SULF1 | cluster_2 | 2.51E-43 | 6.72E-43 |
| SULF2 | cluster_3 | 1.73E-98 | 7.83E-98 |
| SVEP1 | cluster_3 | 3.64E-105 | 1.74E-104 |
| SYNE2 | cluster_3 | 7.99E-76 | 2.98E-75 |
| SYNPO2 | cluster_3 | 3.04E-94 | 1.34E-93 |
| SYT1 | cluster_2 | 0.387210447 | 0.400225924 |
| SYT4 | cluster_3 | 7.19E-47 | 2.01E-46 |
| SYT6 | cluster_3 | 0.000304097 | 0.000374039 |
| TAC1 | cluster_3 | 4.24E-63 | 1.41E-62 |
| TACC1 | cluster_3 | 4.12E-257 | 4.17E-256 |
| TACSTD2 | cluster_3 | 4.59E-05 | 5.90E-05 |
| TAGLN | cluster_2 | 0 | 0 |
| TBC1D3D | cluster_2 | 0.001350787 | 0.001610842 |
| TBCE | cluster_3 | 7.48E-12 | 1.23E-11 |
| TBX2 | cluster_2 | 7.11E-21 | 1.39E-20 |
| TBX3 | cluster_3 | 4.66E-05 | 5.98E-05 |
| TCEAL2 | cluster_3 | 0.139818743 | 0.148256081 |
| TCF23 | cluster_3 | 1.92E-09 | 2.92E-09 |
| TCIM | cluster_3 | 4.45E-239 | 4.28E-238 |
| TCIRG1 | cluster_1 | 1.15E-34 | 2.78E-34 |
| TCL1A | cluster_1 | 7.68E-06 | 1.03E-05 |
| TDO2 | cluster_2 | 0 | 0 |
| TENM2 | cluster_2 | 7.61E-05 | 9.67E-05 |
| TENM4 | cluster_2 | 6.35E-23 | 1.28E-22 |
| TEX9 | cluster_1 | 2.27E-05 | 2.98E-05 |
| TFEC | cluster_2 | 2.70E-05 | 3.51E-05 |
| TFF3 | cluster_3 | 8.70E-10 | 1.34E-09 |
| TFPI | cluster_3 | 2.31E-58 | 7.38E-58 |
| TFPI2 | cluster_3 | 6.82E-13 | 1.14E-12 |
| TFRC | cluster_2 | 1.55E-73 | 5.66E-73 |
| TGFB2 | cluster_3 | 6.48E-07 | 9.03E-07 |
| TGFBI | cluster_1 | 0 | 0 |
| TGFBR2 | cluster_3 | 2.61E-137 | 1.49E-136 |
| TGFBR3 | cluster_3 | 6.85E-184 | 5.17E-183 |
| TGFBR3L | cluster_1 | 0.060825269 | 0.066166212 |
| TGM2 | cluster_3 | 2.32E-72 | 8.37E-72 |
| TGM5 | cluster_1 | 0.031811169 | 0.035204905 |
| TGM7 | cluster_1 | 1 | 1 |
| THBD | cluster_1 | 1.56E-08 | 2.29E-08 |
| THBS1 | cluster_3 | 3.88E-271 | 4.15E-270 |
| THBS2 | cluster_2 | 1.08E-160 | 7.30E-160 |
| THBS4 | cluster_3 | 0 | 0 |
| THY1 | cluster_2 | 2.52E-15 | 4.41E-15 |
| TIMP1 | cluster_1 | 0 | 0 |
| TIMP3 | cluster_1 | 5.40E-58 | 1.71E-57 |
| TIMP4 | cluster_1 | 8.37E-05 | 0.0001061 |
| TINAGL1 | cluster_3 | 0.000692535 | 0.000837462 |
| TIPARP | cluster_3 | 0 | 0 |
| TK1 | cluster_1 | 1.92E-64 | 6.49E-64 |
| TLL1 | cluster_3 | 0.639532942 | 0.648572277 |
| TLNRD1 | cluster_3 | 2.52E-63 | 8.44E-63 |
| TM4SF1 | cluster_3 | 1.43E-151 | 8.84E-151 |
| TM4SF18 | cluster_3 | 0.228587424 | 0.240309856 |
| TM4SF19 | cluster_1 | 0.544673667 | 0.555644582 |
| TMEM100 | cluster_1 | 5.32E-69 | 1.85E-68 |
| TMEM119 | cluster_2 | 2.37E-177 | 1.74E-176 |
| TMEM132A | cluster_2 | 2.83E-55 | 8.71E-55 |
| TMEM150C | cluster_3 | 2.44E-07 | 3.44E-07 |
| TMEM158 | cluster_2 | 1.32E-223 | 1.17E-222 |
| TMEM176A | cluster_3 | 1.82E-223 | 1.61E-222 |
| TMEM176B | cluster_3 | 1.05E-134 | 5.89E-134 |
| TMEM196 | cluster_1 | 1.97E-09 | 3.00E-09 |
| TMEM233 | cluster_3 | 2.27E-05 | 2.98E-05 |
| TMEM255B | cluster_1 | 0.000362542 | 0.000444339 |
| TMOD1 | cluster_1 | 1.36E-16 | 2.45E-16 |
| TNC | cluster_1 | 4.55E-186 | 3.50E-185 |
| TNF | cluster_2 | 2.19E-08 | 3.19E-08 |
| TNFAIP2 | cluster_3 | 2.15E-93 | 9.36E-93 |
| TNFAIP3 | cluster_3 | 1.31E-30 | 2.96E-30 |
| TNFAIP6 | cluster_1 | 5.95E-152 | 3.69E-151 |
| TNFRSF11B | cluster_1 | 4.09E-32 | 9.59E-32 |
| TNFRSF12A | cluster_1 | 2.31E-36 | 5.69E-36 |
| TNFRSF13C | cluster_3 | 0.000141885 | 0.000177305 |
| TNFRSF18 | cluster_1 | 0.030198529 | 0.033484783 |
| TNFRSF1B | cluster_2 | 1.05E-08 | 1.55E-08 |
| TNFRSF21 | cluster_2 | 4.39E-24 | 8.95E-24 |
| TNFRSF4 | cluster_1 | 0.00077878 | 0.000940435 |
| TNFRSF6B | cluster_1 | 3.43E-159 | 2.26E-158 |
| TNFSF10 | cluster_2 | 0.012456797 | 0.014149476 |
| TNFSF11 | cluster_1 | 4.81E-133 | 2.69E-132 |
| TNFSF13B | cluster_3 | 1.82E-31 | 4.20E-31 |
| TNFSF14 | cluster_3 | 1.54E-24 | 3.17E-24 |
| TNFSF15 | cluster_1 | 1.45E-27 | 3.12E-27 |
| TNIK | cluster_2 | 7.87E-36 | 1.93E-35 |
| TNIP3 | cluster_2 | 8.79E-37 | 2.18E-36 |
| TNMD | cluster_3 | 9.62E-85 | 3.89E-84 |
| TNNT1 | cluster_3 | 0.771732008 | 0.779426697 |
| TNNT3 | cluster_3 | 4.94E-160 | 3.28E-159 |
| TNRC6C | cluster_3 | 1.64E-102 | 7.66E-102 |
| TNXB | cluster_1 | 0 | 0 |
| TPD52L1 | cluster_1 | 0.013045977 | 0.014789449 |
| TPM1 | cluster_2 | 0 | 0 |
| TPM2 | cluster_2 | 0 | 0 |
| TPM4 | cluster_2 | 1.01E-150 | 6.22E-150 |
| TPPP3 | cluster_1 | 3.10E-14 | 5.31E-14 |
| TPSAB1 | cluster_3 | 9.64E-10 | 1.48E-09 |
| TPSB2 | cluster_3 | 2.30E-26 | 4.85E-26 |
| TPX2 | cluster_3 | 0.006207838 | 0.007155219 |
| TRABD2A | cluster_3 | 3.40E-10 | 5.31E-10 |
| TRAC | cluster_1 | 2.25E-11 | 3.64E-11 |
| TRAF3IP3 | cluster_2 | 0.00327784 | 0.003831935 |
| TRBC1 | cluster_2 | 4.24E-11 | 6.81E-11 |
| TRBC2 | cluster_2 | 1.78E-11 | 2.89E-11 |
| TREM1 | cluster_1 | 0 | 0 |
| TREM2 | cluster_2 | 7.51E-05 | 9.56E-05 |
| TRGV10 | cluster_2 | 0.439954981 | 0.452570178 |
| TRHDE | cluster_3 | 1.42E-25 | 2.97E-25 |
| TRIB1 | cluster_3 | 2.56E-223 | 2.26E-222 |
| TRIB3 | cluster_2 | 0.030070229 | 0.033364005 |
| TRPA1 | cluster_2 | 1.23E-13 | 2.08E-13 |
| TSC22D1 | cluster_3 | 9.29E-183 | 6.96E-182 |
| TSC22D2 | cluster_3 | 8.16E-132 | 4.53E-131 |
| TSHZ2 | cluster_3 | 1.17E-245 | 1.15E-244 |
| TSLP | cluster_1 | 0.385228699 | 0.398416709 |
| TSPAN18 | cluster_2 | 1.49E-32 | 3.52E-32 |
| TSPAN2 | cluster_1 | 9.06E-62 | 2.97E-61 |
| TSPYL2 | cluster_3 | 3.84E-89 | 1.61E-88 |
| TSTD1 | cluster_1 | 0.010709819 | 0.01221345 |
| TTN | cluster_3 | 2.89E-230 | 2.63E-229 |
| TTTY14 | cluster_2 | 2.41E-25 | 5.01E-25 |
| TUBA1A | cluster_1 | 3.46E-69 | 1.21E-68 |
| TUBA1B | cluster_1 | 1.38E-190 | 1.07E-189 |
| TUBA4A | cluster_1 | 0.780492525 | 0.787812501 |
| TUBB2B | cluster_3 | 3.44E-201 | 2.77E-200 |
| TUBB3 | cluster_1 | 3.40E-32 | 7.97E-32 |
| TWIST1 | cluster_2 | 0.000229156 | 0.000283686 |
| TWISTNB | cluster_1 | 0.000280951 | 0.000346064 |
| TXLNG | cluster_3 | 7.19E-51 | 2.13E-50 |
| TXNIP | cluster_3 | 0 | 0 |
| TXNL4B | cluster_3 | 1.12E-08 | 1.66E-08 |
| TYMS | cluster_1 | 2.76E-23 | 5.57E-23 |
| TYROBP | cluster_2 | 2.42E-95 | 1.08E-94 |
| UACA | cluster_2 | 4.27E-48 | 1.21E-47 |
| UAP1 | cluster_1 | 5.35E-183 | 4.03E-182 |
| UBD | cluster_2 | 6.00E-07 | 8.36E-07 |
| UBE2C | cluster_1 | 8.92E-09 | 1.32E-08 |
| UCHL1 | cluster_2 | 2.07E-63 | 6.94E-63 |
| UCN | cluster_1 | 0.074196622 | 0.080356342 |
| UCP2 | cluster_1 | 1.71E-19 | 3.25E-19 |
| UGDH | cluster_1 | 4.70E-32 | 1.10E-31 |
| UGP2 | cluster_1 | 3.91E-39 | 9.90E-39 |
| UNC5B-AS1 | cluster_1 | 2.76E-06 | 3.78E-06 |
| UPP1 | cluster_2 | 1.53E-28 | 3.35E-28 |
| USP53 | cluster_1 | 2.22E-85 | 9.04E-85 |
| VAMP8 | cluster_1 | 1.77E-44 | 4.82E-44 |
| VASH2 | cluster_3 | 4.08E-19 | 7.72E-19 |
| VASP | cluster_2 | 2.41E-06 | 3.31E-06 |
| VAT1L | cluster_1 | 4.85E-20 | 9.35E-20 |
| VCAM1 | cluster_1 | 7.66E-12 | 1.26E-11 |
| VCAN | cluster_2 | 4.11E-235 | 3.82E-234 |
| VDR | cluster_2 | 1.59E-16 | 2.85E-16 |
| VEGFA | cluster_3 | 2.61E-27 | 5.60E-27 |
| VEGFC | cluster_1 | 2.69E-33 | 6.40E-33 |
| VEGFD | cluster_3 | 8.03E-20 | 1.54E-19 |
| VGLL3 | cluster_3 | 2.40E-19 | 4.56E-19 |
| VIPR2 | cluster_3 | 3.52E-62 | 1.16E-61 |
| VIT | cluster_3 | 6.00E-104 | 2.83E-103 |
| VKORC1 | cluster_1 | 1.04E-149 | 6.30E-149 |
| VMO1 | cluster_3 | 1.05E-134 | 5.89E-134 |
| VMP1 | cluster_3 | 1.30E-16 | 2.34E-16 |
| VNN2 | cluster_2 | 0.177536949 | 0.18732759 |
| VSIG4 | cluster_2 | 1.15E-20 | 2.23E-20 |
| VSIR | cluster_1 | 1.57E-30 | 3.55E-30 |
| VWF | cluster_3 | 0.00209727 | 0.002472578 |
| WARS | cluster_2 | 1.85E-18 | 3.45E-18 |
| WDR86 | cluster_2 | 1.40E-40 | 3.62E-40 |
| WEE1 | cluster_3 | 8.15E-82 | 3.22E-81 |
| WFDC1 | cluster_1 | 0.001142179 | 0.001368708 |
| WFDC2 | cluster_2 | 3.10E-50 | 9.13E-50 |
| WNT2 | cluster_2 | 5.73E-109 | 2.83E-108 |
| WNT4 | cluster_2 | 1.48E-28 | 3.24E-28 |
| WNT5A | cluster_2 | 1.78E-141 | 1.05E-140 |
| WNT5B | cluster_1 | 1.37E-06 | 1.90E-06 |
| WNT7B | cluster_2 | 3.77E-46 | 1.04E-45 |
| WT1 | cluster_3 | 8.46E-05 | 0.000107231 |
| WWC1 | cluster_2 | 3.18E-36 | 7.84E-36 |
| XAF1 | cluster_2 | 3.78E-49 | 1.09E-48 |
| XCL2 | cluster_2 | 6.22E-08 | 8.89E-08 |
| XIRP1 | cluster_3 | 2.69E-17 | 4.88E-17 |
| XIST | cluster_3 | 1.73E-40 | 4.47E-40 |
| ZBTB16 | cluster_3 | 3.72E-96 | 1.67E-95 |
| ZC3H12C | cluster_3 | 3.91E-09 | 5.88E-09 |
| ZCCHC12 | cluster_3 | 0.042291949 | 0.046475263 |
| ZDHHC14 | cluster_3 | 1.12E-110 | 5.62E-110 |
| ZFAND5 | cluster_3 | 0 | 0 |
| ZFHX4 | cluster_3 | 2.62E-116 | 1.34E-115 |
| ZFP36 | cluster_3 | 0 | 0 |
| ZFP36L1 | cluster_3 | 0 | 0 |
| ZFP36L2 | cluster_3 | 1.39E-219 | 1.19E-218 |
| ZIC1 | cluster_1 | 4.83E-14 | 8.24E-14 |
| ZNF331 | cluster_3 | 2.41E-272 | 2.62E-271 |
| ZNF367 | cluster_1 | 0.466247591 | 0.478473392 |
| ZNF385B | cluster_1 | 6.24E-08 | 8.91E-08 |
| ZNF503 | cluster_3 | 0.833219626 | 0.839557751 |
