# Supplementary material for: Single-cell transcriptome reveals Staphylococcus aureus modulating fibroblast differentiation in the bone-implant interface
Source: Mol Med. 2023 Mar 16;29:35. doi: 10.1186/s10020-023-00632-7 (PMC10021980; doi:10.1186/s10020-023-00632-7)
Supplement: Supplementary file 9 — Additional file 9: Table S3. Differentially expressed genes in fibroblast. [file 10020_2023_632_MOESM9_ESM.doc]

**Additional file 9: Table S3: Differentially expressed genes in fibroblast**

| **Gene symbol** | **p_value** | **avgerage log2FC** | **pct.1^#^** | **pct.2^*^** | **ajusted_p_value** |
| --- | --- | --- | --- | --- | --- |
| PRG4 | 0 | -5.225342884 | 0.102 | 0.721 | 0 |
| MGP | 0 | -3.967582802 | 0.478 | 0.992 | 0 |
| PLA2G2A | 0 | -3.9232982 | 0.299 | 0.804 | 0 |
| CXCL14 | 9.90E-116 | -3.898030854 | 0.03 | 0.295 | 2.57E-111 |
| CD9 | 0 | -3.316420581 | 0.233 | 0.881 | 0 |
| PRELP | 0 | -3.309584994 | 0.151 | 0.866 | 0 |
| RGCC | 0 | -3.237559947 | 0.296 | 0.883 | 0 |
| CRTAC1 | 4.32E-235 | -2.993705934 | 0.208 | 0.62 | 1.12E-230 |
| CLU | 2.96E-194 | -2.821541873 | 0.565 | 0.918 | 7.69E-190 |
| CRIP1 | 0 | -2.815010766 | 0.752 | 0.92 | 0 |
| AMTN | 2.64E-101 | -2.643686135 | 0 | 0.229 | 6.86E-97 |
| TIMP3 | 0 | -2.613572841 | 0.441 | 0.88 | 0 |
| CFD | 3.33E-49 | -2.482731668 | 0.478 | 0.585 | 8.64E-45 |
| THBS4 | 8.76E-276 | -2.397224929 | 0.019 | 0.521 | 2.27E-271 |
| CRLF1 | 0 | -2.390040852 | 0.062 | 0.659 | 0 |
| S100A4 | 0 | -2.334941037 | 0.916 | 0.977 | 0 |
| ADIRF | 0 | -2.164427923 | 0.226 | 0.794 | 0 |
| IGF1 | 2.36E-75 | -2.151153247 | 0.225 | 0.455 | 6.12E-71 |
| MT1G | 5.93E-38 | -2.051163592 | 0.176 | 0.32 | 1.54E-33 |
| MT1X | 8.71E-181 | -2.011320451 | 0.625 | 0.827 | 2.26E-176 |
| S100A10 | 0 | -1.962111402 | 0.952 | 0.991 | 0 |
| COMP | 0 | -1.940974712 | 0.197 | 0.782 | 0 |
| HTRA1 | 3.43E-182 | -1.902453358 | 0.919 | 0.981 | 8.89E-178 |
| ASPN | 8.29E-215 | -1.849154698 | 0.51 | 0.836 | 2.15E-210 |
| MT2A | 8.14E-237 | -1.802577981 | 0.932 | 0.983 | 2.11E-232 |
| ANXA1 | 0 | -1.789012882 | 0.898 | 0.989 | 0 |
| AQP1 | 0 | -1.766786524 | 0.143 | 0.727 | 0 |
| CD55 | 1.95E-254 | -1.764260758 | 0.313 | 0.704 | 5.05E-250 |
| CST3 | 0 | -1.7541586 | 0.966 | 0.998 | 0 |
| RARRES1 | 3.73E-42 | -1.751556269 | 0.363 | 0.462 | 9.69E-38 |
| ACKR3 | 6.98E-288 | -1.722697341 | 0.271 | 0.718 | 1.81E-283 |
| CILP | 3.98E-283 | -1.681479763 | 0.016 | 0.526 | 1.03E-278 |
| SPARCL1 | 1.15E-192 | -1.667622321 | 0.347 | 0.701 | 2.97E-188 |
| TNXB | 0 | -1.649076427 | 0.062 | 0.659 | 0 |
| DEFB1 | 1.59E-99 | -1.643362229 | 0.005 | 0.233 | 4.11E-95 |
| PROCR | 8.48E-124 | -1.641375499 | 0.35 | 0.572 | 2.20E-119 |
| IGFBP5 | 7.69E-126 | -1.564337665 | 0.288 | 0.597 | 2.00E-121 |
| CDO1 | 2.44E-304 | -1.497370739 | 0.114 | 0.631 | 6.32E-300 |
| NDUFA4L2 | 1.33E-85 | -1.491861022 | 0.388 | 0.592 | 3.45E-81 |
| FMOD | 0 | -1.479372571 | 0.441 | 0.826 | 0 |
| GSN | 0 | -1.477151571 | 0.758 | 0.962 | 0 |
| TNC | 1.26E-196 | -1.472571908 | 0.481 | 0.769 | 3.27E-192 |
| DKK3 | 2.47E-290 | -1.453325003 | 0.337 | 0.733 | 6.41E-286 |
| SFRP1 | 2.44E-74 | -1.451685362 | 0.145 | 0.372 | 6.33E-70 |
| TPPP3 | 3.40E-148 | -1.413419972 | 0.365 | 0.643 | 8.83E-144 |
| ACTA2 | 2.68E-123 | 1.403913762 | 0.565 | 0.285 | 6.95E-119 |
| ADRA2A | 1.12E-124 | 1.430498861 | 0.266 | 0.072 | 2.91E-120 |
| IGHG3 | 0 | 1.433920184 | 0.322 | 0.017 | 0 |
| COL5A3 | 0 | 1.488721608 | 0.728 | 0.258 | 0 |
| LBP | 1.11E-166 | 1.579102855 | 0.304 | 0.066 | 2.89E-162 |
| RPS2 | 0 | 1.582551826 | 0.985 | 0.989 | 0 |
| TPM2 | 2.76E-293 | 1.658051491 | 0.92 | 0.769 | 7.16E-289 |
| IL7R | 0 | 1.71415319 | 0.368 | 0.024 | 0 |
| HLA-B | 0 | 1.738989539 | 0.985 | 0.964 | 0 |
| TAGLN | 3.85E-137 | 1.779353744 | 0.732 | 0.458 | 9.99E-133 |
| IGHG1 | 0 | 1.806569648 | 0.294 | 0.019 | 0 |
| IGHG4 | 0 | 1.812800214 | 0.323 | 0.01 | 0 |
| TDO2 | 1.08E-302 | 1.873526571 | 0.319 | 0.03 | 2.81E-298 |
| IGLC2 | 7.66E-168 | 1.925320569 | 0.333 | 0.088 | 1.99E-163 |
| MMP1 | 1.72E-94 | 1.928822247 | 0.221 | 0.058 | 4.46E-90 |
| RPS4Y1 | 0 | 1.949654082 | 0.538 | 0 | 0 |
| HLA-A | 0 | 2.046238077 | 0.98 | 0.947 | 0 |
| MMP13 | 1.28E-123 | 2.179060077 | 0.404 | 0.158 | 3.33E-119 |
| EGFL6 | 7.42E-237 | 2.405760647 | 0.39 | 0.088 | 1.92E-232 |
| CHI3L2 | 4.90E-154 | 2.532898678 | 0.546 | 0.274 | 1.27E-149 |
| IGLC3 | 0 | 2.555546217 | 0.293 | 0.016 | 0 |
| APOE | 0 | 3.1111999 | 0.848 | 0.257 | 0 |
| CXCL13 | 0 | 3.159889604 | 0.279 | 0.014 | 0 |
| IGKC | 7.94E-236 | 3.177615498 | 0.35 | 0.066 | 2.06E-231 |

#pct.1: percent of positive cells in fibroblast from PJI

*pct.2: percent of positive cells in fibroblast from AL
