# Supplementary material for: Single-cell transcriptome reveals Staphylococcus aureus modulating fibroblast differentiation in the bone-implant interface
Source: Mol Med. 2023 Mar 16;29:35. doi: 10.1186/s10020-023-00632-7 (PMC10021980; doi:10.1186/s10020-023-00632-7)
Supplement: Supplementary file 10 — Additional file 10: Table S4. Detailed information for scRNA-seq samples for integration analysis. [file 10020_2023_632_MOESM10_ESM.docx]

**Additional file 10: Table S4. Detailed information for scRNA-seq samples for integration analysis**

| **Tissue** | **Diseases** | **GSE number** | **Replicates** |
| --- | --- | --- | --- |
| Synovium | Osteoarthritis | GSE152805 | 3 |
| Synovium | Osteoarthritis | GSE152815 | 2 |
| Synovium | Osteoarthritis | GSE176308 | 3 |
| Synovium | Rheumatoid arthritis | GSE181082 | 2 |
| Cartilage | Osteoarthritis | GSE152805 | 6 |
| Cartilage | Osteoarthritis | GSE169454 | 4 |
| Cartilage | Normal | GSE169454 | 3 |
| Skin | Normal | GSE186476 | 14 |
| Skin | Lupus | GSE186476 | 14 |
| Colon mucosa | Normal | GSE182270 | 3 |
| Colon mucosa | Colitis | GSE182270 | 6 |
| Lung | Idiopathic Pulmonary Fibrosis， IPF | GSE135893 | 34 |
| Periodontal tissue | periodontitis | GSE181688 | 3 |
| Peri-prosthetic tissue | Peri-prosthetic Joint Infection | This study | 3 |
| Peri-prosthetic tissue | Aseptic Loosening | This study | 2 |
